# Identifying opportunities for late-stage C-H alkylation with high-throughput experimentation and in silico reaction screening

**DOI:** 10.1038/s42004-023-01047-5

**Published:** 2023-11-20

**Authors:** David F. Nippa, Kenneth Atz, Alex T. Müller, Jens Wolfard, Clemens Isert, Martin Binder, Oliver Scheidegger, David B. Konrad, Uwe Grether, Rainer E. Martin, Gisbert Schneider

**Affiliations:** 1 Roche Pharma Research and Early Development (pRED), Roche Innovation Center Basel, F. Hoffmann-La Roche Ltd., Grenzacherstrasse 124, 4070 Basel, Switzerland; 2 Department of Pharmacy, Ludwig-Maximilians-Universität München, Butenandtstrasse 5, 81377 Munich, Germany; 3 Department of Chemistry and Applied Biosciences, ETH Zurich, Vladimir-Prelog-Weg 4, 8093 Zurich, Switzerland

**Keywords:** Cheminformatics, Lead optimization, Computational chemistry, Synthetic chemistry methodology

## Abstract

Enhancing the properties of advanced drug candidates is aided by the direct incorporation of specific chemical groups, avoiding the need to construct the entire compound from the ground up. Nevertheless, their chemical intricacy often poses challenges in predicting reactivity for C-H activation reactions and planning their synthesis. We adopted a reaction screening approach that combines high-throughput experimentation (HTE) at a nanomolar scale with computational graph neural networks (GNNs). This approach aims to identify suitable substrates for late-stage C-H alkylation using Minisci-type chemistry. GNNs were trained using experimentally generated reactions derived from in-house HTE and literature data. These trained models were then used to predict, in a forward-looking manner, the coupling of 3180 advanced heterocyclic building blocks with a diverse set of sp^3^-rich carboxylic acids. This predictive approach aimed to explore the substrate landscape for Minisci-type alkylations. Promising candidates were chosen, their production was scaled up, and they were subsequently isolated and characterized. This process led to the creation of 30 novel, functionally modified molecules that hold potential for further refinement. These results positively advocate the application of HTE-based machine learning to virtual reaction screening.

## Abstract (web summary)

Late-stage functionalization of drug molecules can tune their properties without the need for entirely new syntheses, however, predicting reactivity and planning synthesis for late-stage C-H activation remains challenging. Here, the authors develop a reaction screening approach combining high-throughput experimentation with computational graph neural networks to identify suitable substrates that can be used for late-stage C-H alkylation *via* Minisci-type chemistry.

## Introduction

The synthesis of novel compounds represents the bottleneck in terms of time and effort for numerous small molecule drug discovery projects^1^. Late-stage functionalization (LSF) is a strategy that adds extra functional groups to drug molecules, bypassing the necessity for entirely new synthesis or the requirement for specific functional handles^2^. These subtle structural alterations simplify the process of understanding the relationships between the chemical structure and the biological activity (structure–activity relationships, SARs). Additionally, they allow for the enhancement of pharmacokinetic properties, including absorption, distribution, metabolism, and excretion, in lead compounds and drug candidates^3^. Importantly, these modifications can be achieved with lower synthetic costs^4^. Nonetheless, it is worth noting that not all molecules readily lend themselves to the desired functionalizations, making LSF a challenging process in experimental terms. In response to this challenge, we present a computational machine-learning framework designed for predicting the reactivity of drug molecules. This framework offers a more rational approach to LSF, potentially reducing the time and experimental costs typically associated with this endeavor.

An increasing number of experimental LSF methods have recently been published that allow medicinal chemists to fluorinate, aminate, arylate, methylate, trifluoromethylate, borylate, acylate, or oxidize structurally intricate molecules^5,6^. Alkylation reactions have gained interest as they allow the introduction of small cyclic and acyclic alkyl groups through carbon–carbon, carbon–oxygen, or carbon–nitrogen bond formation^7^. In particular, Minisci-type alkylations^8,9^ are considered a valuable LSF methodology for incorporating alkyl building blocks into heterocyclic systems, which often form the core of drug molecules^10^.

Originally described in the mid-20th century, Minisci reactions have become a versatile tool in medicinal chemistry for the formation of C–C bonds^11^. Using ammonium persulfate as the oxidant and silver nitrate as the catalyst, alkyl radicals are generated from the corresponding carboxylic acids at elevated temperatures. Upon radical addition to the heteroarene, the reaction product is formed through aromaticity-driven oxidation of the radical intermediate^11^. The scope of both, electron-deficient heteroarenes and alkyl-donating coupling partners, has steadily been expanded^12,13^. Various radical sources have been documented in the literature. These include alkyl carboxylic acids capable of transferring alkyl groups, boronic acids suitable for the incorporation of aryl groups, or sulfinates that were used to transfer trifluoromethyl or tert-butyl fragments^14,15^. Employing readily accessible and cost-effective carboxylic acids, without the prerequisite for prefunctionalization, considerably broadens the applicability of this transformation for drug discovery purposes^16^. The growing emphasis on integrating sp^3^-rich building blocks into pharmaceuticals^17^, coupled with the ready availability of stable cyclic alkyl carboxylic acids, renders this approach particularly appealing for expanding hits into lead compounds and optimizing drugs through LSF.

It has become apparent that by decreasing the count of aromatic rings within a drug candidate, the chances of achieving clinical success can be heightened^18^. A higher proportion of sp^3^ centers allows for exploration of novel chemical territory, which can potentially improve drug selectivity^19^. This shift can also positively influence essential physicochemical properties, such as solubility and metabolic stability^20–22^. While guidelines exist for predicting reactivity in Minisci-type transformations, the challenge lies in the limited range of functional groups that can be accommodated, along with the diverse array of C–H bonds and electronic effects within complex molecules. These complexities make the prediction of alkylation reactions a challenging task^4,23^. Conducting individual reactions at the typical scale used in medicinal chemistry (milligram scale) to enrich the reaction database with pertinent transformation examples would be a laborious and resource-intensive undertaking, yielding limited value relative to the effort invested.

High-throughput experimentation (HTE) has emerged as a valuable tool for systematically exploring and optimizing new chemical transformations in a semi-automated manner^24,25^. To effectively accomplish the miniaturization of reactions at the nanomolar scale, it is essential to engineer the system with precision to handle extremely small quantities of materials and ensure consistent and thorough mixing of the reaction components^26^. Advanced technologies like ultra-high-performance liquid chromatography-mass spectrometry enable the analysis and the separation of minute quantities from screening plates^27,28^. Another crucial aspect of HTE involves the careful curation of all collected reaction data, including unsuccessful transformations, in accordance with the FAIR principles (findable, accessible, interoperable, and reusable)^29^. This approach ensures the creation of high-quality datasets suitable for machine learning applications^30–32^.

Graph neural networks (GNNs) that enable efficient learning on three-dimensional (3D) molecular models have found various applications in drug discovery and development^33–35^. In addition to their prominent applications in quantum chemistry^36,37^, GNN methods have been developed for the prediction of forward reactions, starting from small substrates and leading to the synthesis of complex drug molecules^38–40^. Moreover, GNNs have recently found application in LSF to predict reaction yield, binary reaction outcome, and regioselectivity for borylation reactions^41^. A similar methodology has been introduced for predicting late-stage alkylation, with a primary emphasis on Baran-type diversinate chemistry that employs alkyl sodium sulfinate salts^42^. Additionally, a recent investigation has demonstrated that hybrid machine learning models, enriched with quantum chemical details about transition states, can achieve accurate predictions of regioselectivity for iridium-catalyzed borylation reactions, even when operating with limited data^43^.

**Fig. 1:**
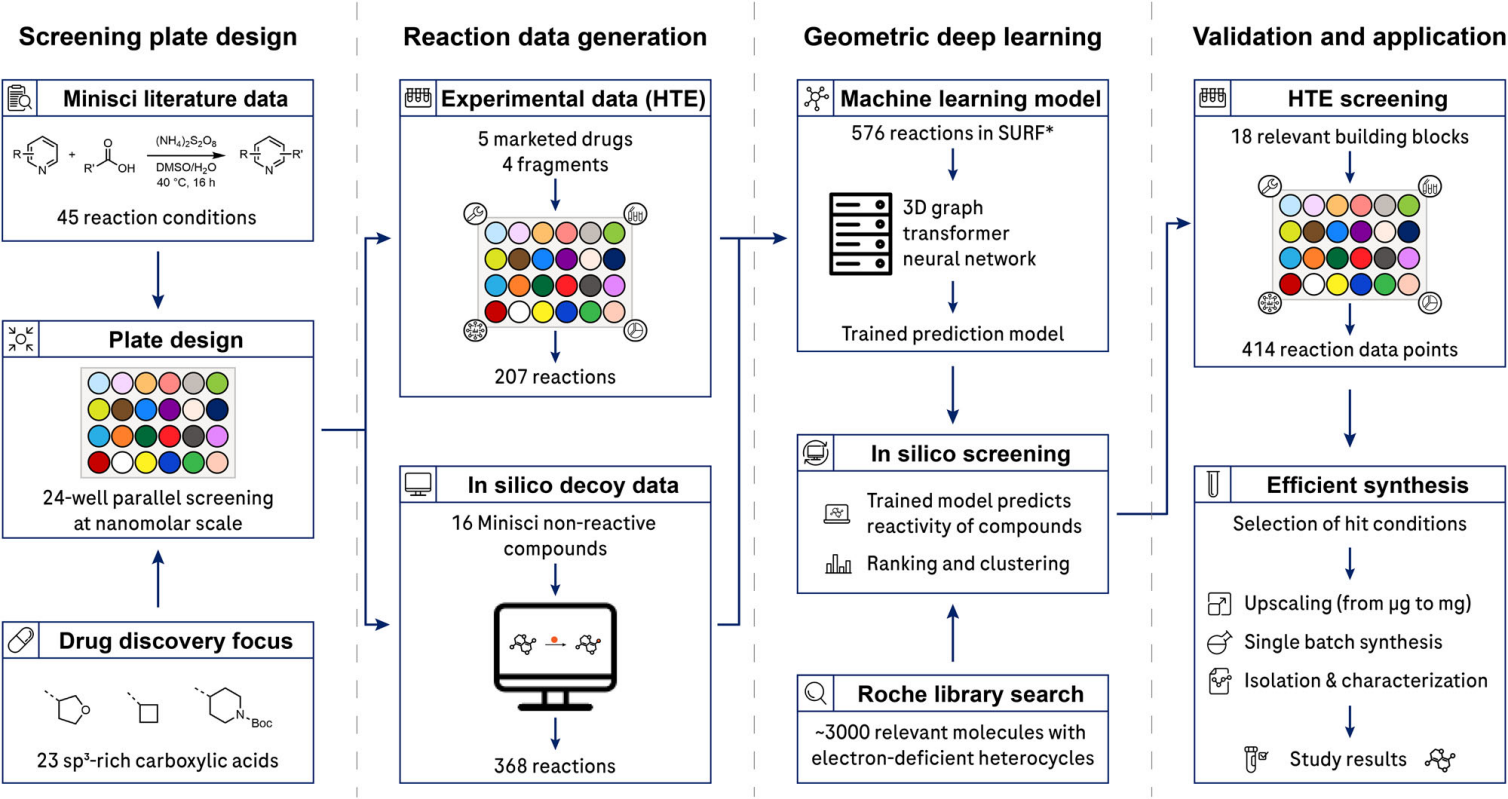
Overview of the research study. *Screening plate design*: Minisci literature data containing metal-free reactions were extracted and analyzed to determine suitable reaction conditions. For parallel reaction screening, 23 sp^3^-rich carboxylic acids with relevance for drug discovery were included. *Reaction data generation*: Using the reaction plate design, physical experiments in high-throughput experimentation (HTE) fashion were conducted with marketed drugs and fragments from an informer library (184 reactions^41^) covering relevant chemical space. In addition, 16 distinctly non-reactive substrates were screened for in silico decoy data generation (368 reactions). *Geometric deep learning*: The obtained reaction data (SURF, Simple User-friendly Reaction Format)^41^ were subjected to geometric deep learning, incorporating 3D structural information of the chemicals. The trained model was applied to 3000 building blocks from the Roche library, with a particular focus on electron-deficient heterocycles. This in silico screening predicted the reactivity of the compounds for substrate ranking and clustering. *Validation and application*: The prediction models were experimentally validated for a diverse set of 18 building blocks. Selected scale-up reactions led to fully characterized compounds.

In this study, we showcase the application of GNNs trained on a limited set of reaction data for machine-learning-based virtual reaction screening. When combined with laboratory automation, this approach has facilitated the discovery of 276 promising alkylation possibilities with high precision (Fig. 1). This effort has resulted in the synthesis of a diverse range of novel compounds characterized by an enhanced sp^3^ fraction.

## Results

### HTE reaction screening

**Fig. 2:**
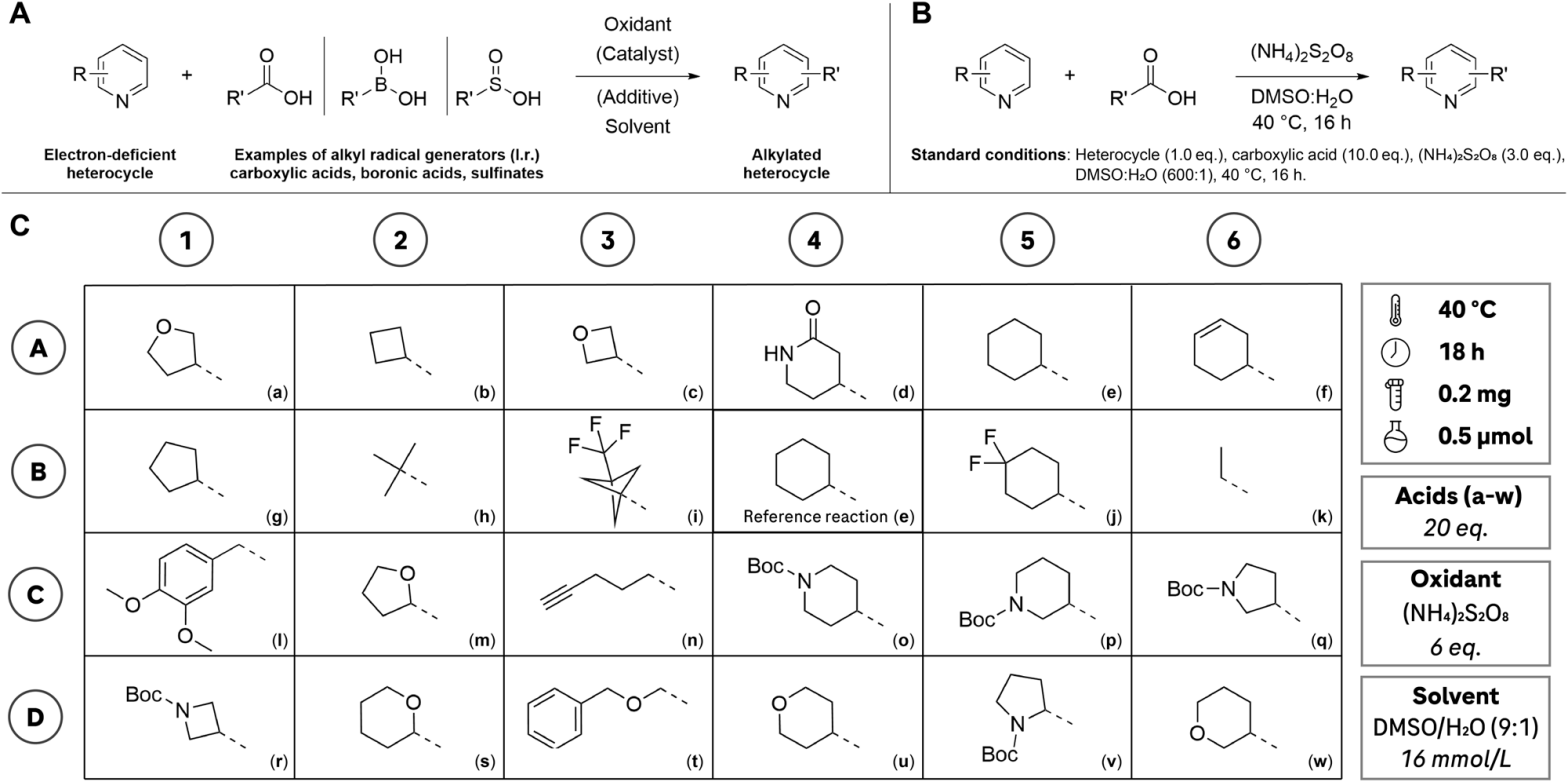
Overview of Minisci-type reactions and screening plate. **A** General reaction scheme of a Minisci-type alkylation reaction. An alkyl substituent obtained from a radical generator, e.g., through decarboxylation of the carboxylic acid, is introduced to an electron-deficient heterocycle, often a pyridine. Depending on the development scope and applied technology, a variety of oxidants, catalysts, additives and solvents are used. **B** Schematic overview of the Minisci-type reaction reported by Sutherland et al.^16^, including the equivalents of the components. **C** Reaction screening plate used in this study. This setup allows to assess the coupling performance of a molecule of interest with 23 different alkyl carboxylic acids (**a**–**w**) that are relevant to medicinal chemistry applications. This configuration enables the evaluation of how well a molecule of interest couples with 23 distinct alkyl carboxylic acids (labeled as **a**–**w**), which are pertinent to medicinal chemistry applications. Condition B4 served as a reference reaction, ensuring consistent performance under the applied conditions. On all screening plates, B4 comprised starting material **1** and carboxylic acid **e**, providing a quality control mechanism for the generated data. If B4 had not yielded the expected outcome, the entire plate would have been reprocessed. The reaction conditions were adjusted to allow miniaturized parallel reaction screening on a nanomolar scale (0.5 μmol). Boc *tert*-Butyloxycarbonyl, DMSO dimethylsulfoxide.

The Minisci-type reactions described by Sutherland et al.^16^ were effectively downscaled from a micromolar (150 μmol) to a nanomolar (500 nmol) level in a parallel configuration using a 24-well plate, achieving a reduction factor of 300 (Fig. 2A, B). Throughout the optimization process, it became evident that the reaction yields substantially improved when performed inside a glovebox. Conducting the reaction with 23 distinct carboxylic acids labeled as **a-w** (Fig. 2C) at various temperatures revealed that the highest conversions were achieved at 40 °C. Elevating the temperature beyond this point primarily resulted in the formation of di-alkylation products. To attain increased conversions, we doubled the amounts of alkyl carboxylic acids (20 equivalents instead of 10) and oxidants (6 equivalents instead of 3). This adjustment led to higher conversions, with an average improvement factor of 1.2–1.5. We included a reference reaction involving Quinoline **1** and carboxylic acid **e** in position B4 (Fig. 2C) to monitor potential performance variations and to ensure the reproducibility of the screening results. Since this reaction is anticipated to consistently yield the desired outcome under the specified conditions, any unexpected outcome in this well would serve as a warning sign, indicating the potential influence of external factors or mishandling of the plates. Such deviations would prompt concerns regarding data reliability. Therefore, in the final configuration, we assessed the integration of 23 diverse alkyl groups, with a primary emphasis on compact sp^3^ ring systems, into electron-deficient heterocycles.

Binary reaction outcomes were labeled as “successful” when the chosen substrate, under the specified reaction conditions, produced a mono- or di-alkylation product that could be confirmed by liquid chromatography-mass spectrometry (LCMS) with a threshold of 5%. Conversely, outcomes were classified as “unsuccessful” when the intended transformation could not be detected through LCMS. In cases of di-alkylation, we consistently observed three distinct products: mono-alkylation on the two distinct carbons and di-alkylation on both. To facilitate the training of machine learning models, the yields of all three reaction products were combined together. Four fragments (**1**–**4**, Supplementary Note 5, Fig. S2) and five drug molecules (**5**–**9**, Supplementary Note 5, Fig. S2) from a chemically diverse LSF informer library^41^, and 18 fragments (**26**–**43**) from the Roche compound library were screened under these reaction conditions. The collected data resulted in a balanced experimental data set comprising 691 reactions, with 379 classified as successful and 312 as unsuccessful.

### Machine learning-based in silico reaction screening

**Fig. 3:**
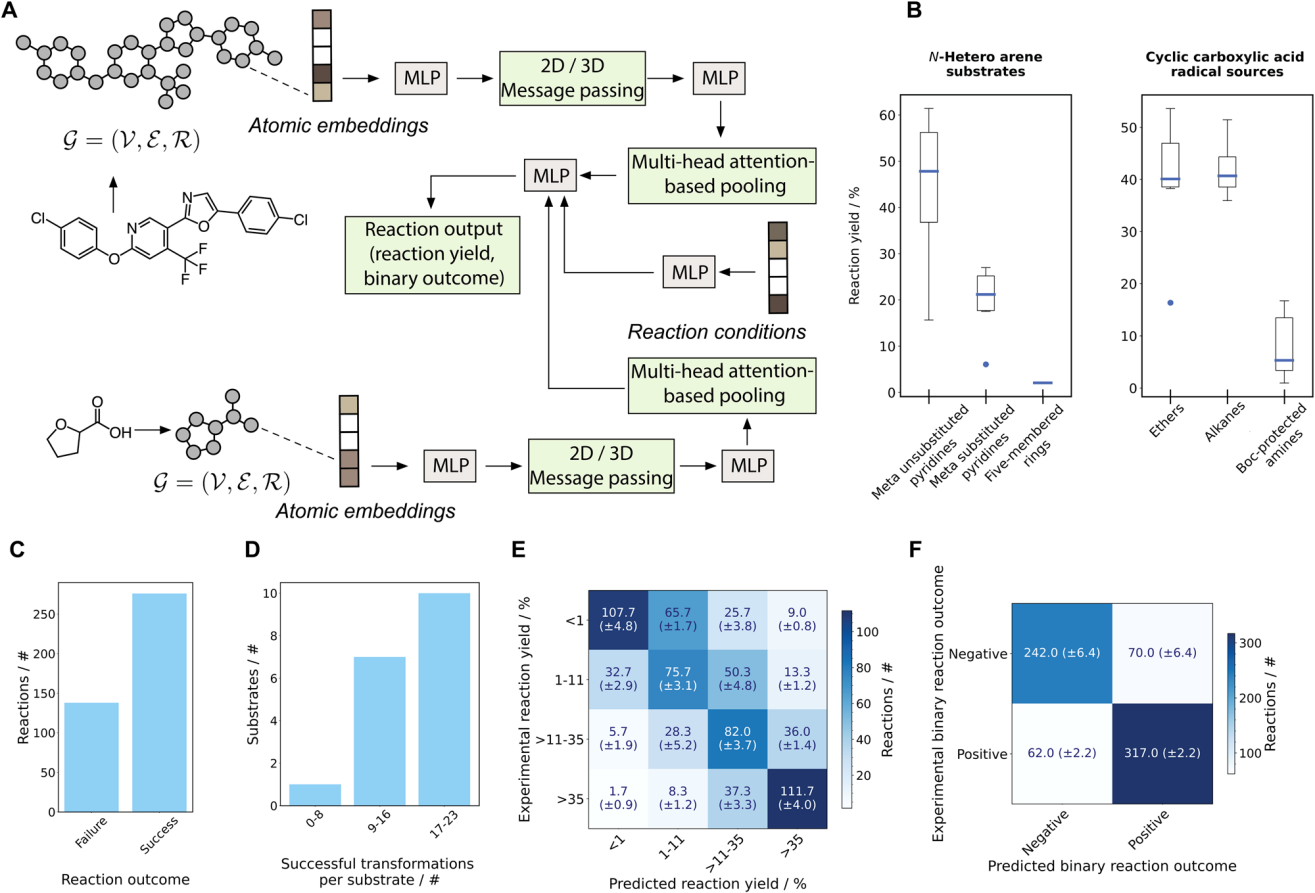
Machine learning and in silico reaction screening results. **A** Schematic of the graph neural networks (GNNs) implemented within the geometric deep learning platform. Multi-layer Perceptron (MLP) modules are highlighted in gray, and the variable modules (2D/3D convolution), pooling, and outputs are highlighted in green. **B** Box plot illustrating trends observed for *N*-hereto arene (left) and carboxylic acids (right). *N*-hetero arenes: Meta-unsubstituted pyridines are observed with a reaction yield of 44 ± 15%, meta-substituted pyridines with 20 ± 6% (including **27** as an outlier observed at 6%), and five-membered *N*-heterocyclic ring systems with 2 ± 1%. Carboxylic acids: Cyclic ethers are observed with a reaction yield of 40 ± 12%, (including **c** as an outlier observed at 16%), cyclic alkanes with 42 ± 6%, and Boc-protected amines with 8 ± 6%. The error bars on both box plots represent 95% confidence intervals, the bottom and top of the box are the 25th and 75th percentiles, the line inside the box is the 50th percentile (median), and any outliers are shown as open circles. **C** Bar plot illustrating the number of successful and failed reactions from HTE. The substrates selected by the model resulted in 276 successful reaction outcomes. **D** Bar plot illustrating the number of unique alkylation opportunities identified per substrate. The majority of *N*-hetero arenes (10/17) allowed for successful transformation with 17–23 carboxylic acids. **E** Confusion matrix for reaction yield prediction. Reaction yields are divided into four bins, namely, no reaction (≤1%), poor (>1–11%), medium (>11–35%), and high reaction yield (>35%). The model accurately predicts 54.6 (±0.9)% of the reactions into the accurate bin, achieves a mean absolute error (MAE) of 18.7 (±0.2)% and a Pearson correlation coefficient (*r*) of 0.687 (±0.006). **F** Confusion matrix for binary reaction outcome prediction achieving an absolute accuracy of 80.8 (±1.2) and an *F*-score of 82.7 (±0.6)%.

GNN models (Fig. 3A) were trained using an initial dataset of 621 Minisci reactions, comprising 368 generated as decoys, 45 from the literature, and 207 from the LSF informer library. These models enabled in silico reaction screening of a Roche in-house library of 3180 advanced heterocyclic building blocks. Each substrate was assigned an ensemble score, which was determined by aggregating the predictions from six independent models. Specifically, this ensemble score incorporated inputs from three models for binary reaction outcome prediction and three models for reaction yield prediction (“Graph neural network architecture”). Subsequently, the molecules were grouped into eight clusters using agglomerative compound clustering (Supplementary Note 2). Two compound clusters were excluded from consideration due to the prevalence of unsuitable structures, namely heterocycles lacking free C-H bonds, for the studied reaction. From the six remaining clusters, three molecules were chosen from each, based on their computed reactivity score, resulting in a total of 18 *N*-heteroarenes.

**Fig. 4:**
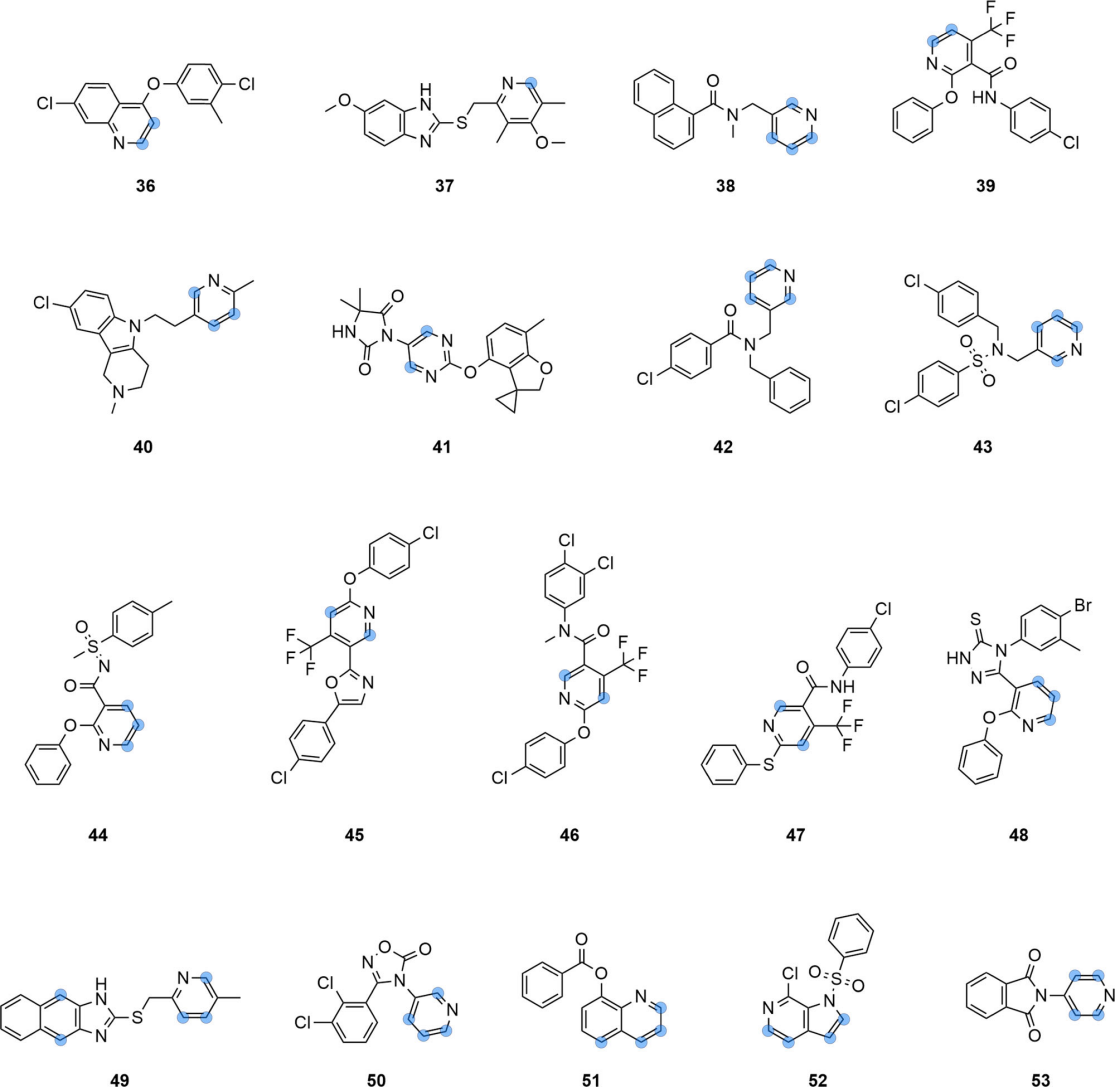
Overview of selected substrates suggested by the in silico prediction model. Structures of the 18 selected substrates **36**–**53** that were suggested by the graph neural networks as suitable for Minisci-type alkylation and underwent subsequent screening to identify novel starting points. Potential, not confirmed, carbon reaction centers are marked with a blue dot.

The selected 18 *N*-hetero arenes were subjected to automated HTE screening, generating an experimental data set of 414 reaction points. For each of the selected substrates, Minisci-type alkylation products could be identified, resulting in a total of 276 successful reactions (Fig. 3C). Among the screened *N*-heteroarenes, 10 of them facilitated between 17 and 23 successful transformations across the chosen carboxylic acids. (Fig. 3D). 7 *N*-hetero arenes allowed 10-17 successful transformations. For one substrate, specifically the meta-substituted pyridine **42** (Fig. 4), fewer than ten successful reactions were observed (Fig. 3D). Hence, for 17 out of the 18 chosen *N*-heteroarenes, a wide variety of successful Minisci-type alkylation products were identified, resulting in a 94% success rate for substrate selection. However, it is worth noting that there were three five-membered *N*-heterocyclic ring systems (**2,** **4,** **9**) in the LSF informer library, for which very low reaction yields (≤4%, averaged over 23 carboxylic acids) were observed.

To evaluate the overall performance of the GNN models that were trained on the complete experimental data set comprising 691 Minisci reactions obtained via high-throughput experimentation (207 from the LSF informer library and 414 from the virtual reaction screening), these models underwent validation for predicting reaction yield and binary reaction outcomes. This validation was conducted using a random data set split. The reaction yields were predicted with a mean absolute error (MAE) of 18.7 (±0.2)% and a Pearson correlation coefficient (*r*) of 0.687 (±0.006) (Fig. 3E). Reaction yields were categorized into four ranges: no reaction (<1% yield), poor (>1–11%), medium (>11–35%), and high reaction yield (>35–100%). The model predicted the correct category in 55.7 (±0.7)% of the cases. Binary reaction outcomes were predicted with an absolute accuracy of 81 (±1), and an *F*-score of 82.7 (±0.6)% (Fig. 3F). The failed machine learning predictions with an MAE ≥ 70% (i.e., outliers) are illustrated and discussed in Supplementary Note 11 and Table S3.

### Scale-up

**Fig. 5:**
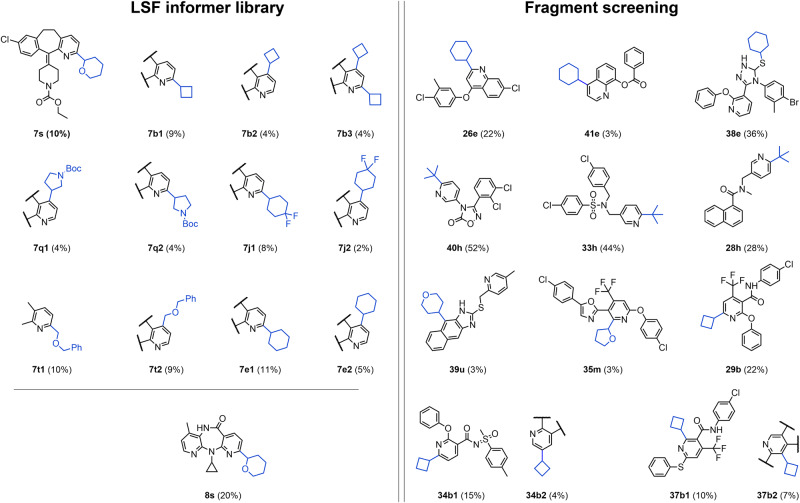
Selected examples of characterized Minisci reaction products. The left panel shows examples from the LSF drug informer library and the right panel from the fragment screening. The added alkyl groups are highlighted in blue. Late-stage drug alkylation examples include derivates of the drugs Loratadine (**7s**, **7b1**, **7b2**, **7b3**, **7q1**, **7q2**, **7j1**, **7j2**, **7t1**, **7t2**, **7e1**, **7e2**) and Nevirapine (**8s**). Fragment screening highlights the diverse range of introduced substituents, covering cyclohexanes (**26e**, **41e**, **38e**), cyclobutanes in different positions (**29b**, **34b1**, **34b2**, **37b1**, **37b2**), heterocyclic alkanes (**39u**, **35m**) and *tert*-butyl (**40h**, **33h**, **28h**). Boc tert-Butyloxycarbony, Ph Phenyl.

Selected screening conditions were used for upscaling to the milligram range. LSF alkylation was carried out for the drug molecules Loratadine (**7**) and Nevirapine (**8**), and structurally complex molecular fragments. In total, 30 novel molecules were synthesized, isolated, and characterized by nuclear magnetic resonance (NMR) spectroscopy and high-resolution mass spectrometry (HRMS) (Fig. 5).

For Loratadine (**7**), a molecule from the LSF informer library, several analogs with different cyclic (**7b1,** **7b2,** **7b3,** **7j1,** **7j2,** **7e1,** **7e2**) and heterocyclic (**7s,** **7q1,** **7q2**) substituents were generated. Structurally complex scaffolds with high relevance for medicinal chemistry projects, which could serve as starting points for the development of SAR studies, also provided a variety of compelling alkylation products. Different alkyl groups, covering alkyl chains (e.g., **40h,** **33h,** **28h**), cyclic alkyls (e.g., **26e,** **41e,** **38e**) and cyclic ethers (e.g., **39u,** **35m**) could be introduced. In general, the observed regiochemistry was consistent with Minisci guidelines, with the alkyl groups being introduced in either the ortho- or para-position on the pyridine core^23^. For molecule **38**, different reactivity was observed with the cyclohexyl radical reacting exclusively with the thiocarbonyl functionality affording thioether **38e**. No reaction at the pyridine core was observed.

### Reactivity trends

Examination of the produced data unveiled a diverse range of observed reaction yields for both the carboxylic acids and the *N*-heteroarenes. Cyclic ethers (e.g., **u,** **s,** **a**) and alkanes (e.g., **b,** **e,** **g**) were reliably converted to the desired alkylation product, whereas cyclic boc-protected amines (e.g., **o,** **p,** **q,** **r**) and amides (**d**) resulted in low yields of the respective desired reaction products (Fig. 3B). Similarly, substituted pyridines (e.g., **30,** **31,** **36,** **39**; see Fig. 4) had lower yields compared to compounds lacking a meta-substituent (e.g., **26,** **32,** **38,** **41**; see Fig. 4). Electron-rich meta-substituted pyridines, such as **3** and **27**, had a comparably low average reaction yield compared to their less electron-rich analogs. Overall, compared to their six-membered *N*-hetero analogs, five-membered *N*-heterocyclic ring systems (e.g., **2,** **4,** **9**; see Supplementary Note 5, Fig. S2) did not show meaningful conversion to the desired alkylation product.

## Discussion

The Minisci reaction conditions, utilizing ammonium persulfate ((NH_4_)_2_S_2_O_8_) as the oxidizing agent and dimethyl sulfoxide (DMSO) as the solvent at a temperature of 40  °C, were effectively downsized and adapted into a parallel screening format. This format allowed for the efficient and resourceful execution of the reaction with a diverse range of alkyl carboxylic acids. The refined reaction protocol facilitates rapid, metal-free, and resource-efficient assessment of reaction conditions in an HTE-compatible format, aiding in informed choices for subsequent synthesis steps. Importantly, it eliminates the need for time-consuming individual reactions conducted on a milligram scale. Nonetheless, this setup has inherent limitations that merit attention in future research:

(i) The current plate design focuses on a single set of reaction conditions for the sake of simplicity. However, examining additional oxidants or solvents, along with adjusting the equivalents of reaction components, holds the potential to deliver further enhancements in reaction yields. Moreover, Minisci-type reactions typically involve metal catalysis, such as with silver or iron^10^. A systematic HTE exploration of various metal salts could lead to the discovery of even more optimized conditions.
(ii) Instead of relying exclusively on carboxylic acids as the source of alkyl radicals, alternative radical precursors like boronic acids or sulfinates could be investigated^13^. This exploration might broaden the range of alkyl groups accessible for medicinal chemistry.
(iii) Several photochemical Minisci-type transformations have been reported^13^. These reactions offer alternative mechanisms for radical generation that could further expand the possibilities for late-stage functionalization (LSF).

Addressing these points in future research could enhance the utility and scope of the Minisci reaction protocol.

The adoption of the user-friendly reaction data format (SURF^41^), facilitated the collection of reaction data from literature sources and enabled standardized reporting of results from HTE and virtual reaction screening. Sharing reaction data in a standardized format plays a pivotal role in the effective utilization of machine learning models for predicting chemical reactivity^44,45^. By using SURF, the initial reaction data from three distinct sources (45 from literature, 207 from experiments, and 368 decoy reactions) became readily available for machine learning, obviating the need for manual data curation. Since both the experimental and, particularly, the literature data are predominantly comprised of positive results, incorporating decoy data from unsuccessful transformations played a crucial role in constructing a dependable prediction model.

A detailed look at the experimental data revealed that cyclic Boc-protected amines (**o,** **p,** **q,** **r,** **v**), as well as amides (e.g., **d**) mainly afforded low yields (5–20%) of the desired reaction products (Supplementary Note 10, Fig. S10). This observation reflects the half-lives of the generated radical intermediates^46^, e.g., with tertiary carbon radicals (e.g., **h**) having higher stability than primary carbon radicals (e.g., **k**) and the latter thus resulting in lower product yields. Another experimental trend relates to the substitution pattern of *N*-heteroarenes. Meta-unsubstituted pyridines (e.g., **26,** **32,** **41**) consistently provided higher yields than substituted analogs, (e.g., **35,** **36,** **37**) as residues on the meta-position sterically hinder the reaction in ortho- and para-positions to the pyridine (Supplementary Note 10, Fig. S11). Finally, electron-rich meta-substituted pyridines, such as **3** and **27**, had a very low (5–10%) average reaction yield on the screening plate when compared to their less electron-rich analogs (Supplementary Note 10, Fig. S10). This low reactivity is owed to the electron-rich amine- and methoxy-substituents, respectively^23^.

In contrast to a prior study^41^ where GNNs processed a single graph input, the GNN model outlined in this research accommodates two distinct molecular inputs, corresponding to the two reactants (*N*-heteroarenes and carboxylic acids). The network architecture was tailored to the Minisci-type alkylation transformation in such a way that trained GNNs can be applied to novel *N*-hetero arenes as well as carboxylic acids. Therefore, the model can be used for in silico molecular library screening for both types of reaction inputs. It could be shown that in silico reaction screening using GNN models trained on a comparably small preliminary data set consisting of 576 Minisci reactions (i.e., 368 from decoy generation, 45 from literature, and 207 LSF from an informer library) led to the identification of 17 substrates (i.e., 94% of the 18 selected molecules). All newly identified substrates were successfully alkylated with a broad range of at least 10 different carboxylic acids. Furthermore, in total 276 successful reactions (i.e., producing alkylation products with a median yield of 26%) were identified. The low reaction yields observed for three five-membered *N*-heterocyclic ring systems (**2,** **4,** **9**) indicate that the GNN models learned to de-prioritize five-membered *N*-hetero arenes during in silico reaction screening. It was shown how a clustering approach can be combined with in silico reaction screening to assess structural diversity as well as reactivity. As previously reported^41^, the inclusion of partial charges did not yield improved model performance (Supplementary Note 3). This observation, in particular, led to the decision to prospectively apply GTNN models that are trained on 3D molecular graphs without electronic features. Further investigations involving more specific electronic features, such as transition state energies, could offer deeper insights into the relevance of quantum chemical attributes in machine learning for reaction prediction, as demonstrated in a recent study^43^. Moreover, the introduced GNNs could be further advanced to facilitate regioselectivity prediction or the prediction of multiple output properties. For instance, this could encompass predicting the proportions of mono- and di-alkylation.

With the overall goal of synthesizing novel scaffolds that are relevant to medicinal chemistry, the visualized screening data served to identify appropriate reaction conditions for upscaling to the milligram scale. Again, the SURF data format was instrumental for the laboratory chemist to set up experiments efficiently by providing the CAS number, SMILES string, equivalents, and overall reaction conditions in a comprehensive and easily accessible format. The reaction conditions were reproducible at a higher scale, underscoring the applicability of this approach to drug discovery. With the exception of compound **38e**, all reactions yielded C-C coupling products. In general, the observed regioselectivity was in agreement with the expected reaction products according to the rules reported in the literature^23^.

However, when moving to more densely functionalized pyridines, these reported literature guidelines do not appear to apply. While the reaction of **34b** and **37b** primarily generated the expected ortho-substituted reaction products **34b1** and **37b1**, also meta-substituted reaction products **34b2** and **37b2** were obtained, albeit in lower amounts (Fig. 5). In the literature, amides are described as ortho-para directing groups due to their electron-withdrawing effect, and aryl ethers as ortho-activating moieties due to their electron-donating nature^23^. The formation of regioisomer **34b2** might have been sterically hindered by the amidyl side chain, favouring the meta- over the para position. For **37b2**, an explanation of the formation could lie in the several different functional groups that are attached to the pyridine ring, which only leave the meta position available for substitution, despite this position being sterically hindered by the proximity of the aryl sulfide and the CF_3_ group. Lastly, **38e** showed different reactivity despite bearing a pyridine moiety. This observed reaction product can be rationalized by the greater reactivity of the lone pairs of the sulfur as compared to the C-H bonds of the pyridine side-chain. These results of the scale-up reactions underscore the importance of generating high-quality, single-batch LSF reaction data.

For the continued development of this method further exploration of Minisci-type reaction conditions is warranted, including the variation of oxidation reagents, solvents, and the incorporation of techniques like photoredox catalysis and electrochemistry^47^. Also, the source of the alkyl radical precursor could be diversified, leading to an expansion of the scope for alkyl groups. Additionally, the substrate scope could be expanded to include other electron-deficient heterocyclic systems, particularly five-membered heterocycles, as they are commonly found motifs in drug-like molecules. With these possibilities in mind, the results of this study emphasize the feasibility and benefits of combining laboratory automation, parallel miniaturized screening, and machine learning to enhance the efficiency and cost-effectiveness of synthesis in drug discovery. This integrated approach is currently being effectively employed at Roche. The predictive capabilities of the computational model will be continuously enhanced by supplying the algorithm with a growing data set of newly generated LSF reaction data points that encompass the pertinent medicinal chemistry landscape.

## Methods

### Literature analysis

A systematic analysis of chemical transformations was carried out to determine the most feasible conditions for reaction miniaturization and parallel screening. Initially, 45 publications covering different Minisci-type alkylation reactions were selected. Most of those methods rely on photo- or electrochemistry. Although it has been demonstrated that these approaches are amenable to HTE^48,49^, carrying out these reaction processes requires specialized equipment that is not readily available in every laboratory. Therefore, with the goal of enabling widespread use in medicinal chemistry, publications were scrutinized for a rapid, resilient, and easily customizable procedure. Sutherland et al.^16^ reported a Minisci methodology that fulfilled those criteria. This transformation can be executed without the necessity for additional metals and catalysts, and it can accommodate a variety of alkyl carboxylic acids that do not demand pre-functionalization. This adaptability allows for the creation of customized templates tailored to specific project requirements. Consequently, the reaction data were manually curated and standardized in a simple user-friendly reaction format (SURF, for details, refer to Supplementary Note 9). These SURF data were used as literature data set herein. All details of the literature analysis (Supplementary Note 4) and the resulting data set in SURF are available as supplementary information (Supplementary Note 4).

### Screening plate design and testing

The screening plate was designed around the literature data obtained from Sutherland et al.^16^, which showed good yields on average (60%) for a variety of carboxylic acid coupling partners. Aiming at assessing the reactivity of a substrate with a variety of different alkyl groups (rings and chains), a screening plate with 23 different alkyl carboxylic acids was assembled. The carboxylic acids scope from the original publication^16^ covering n-alkyl (e.g., **h,** **k**, depicted in Fig. 2), cyclic alkanes (e.g., **e,** **g**) and *O*-heterocyclic fragments (e.g., **m,** **u**) was complemented by sp^3^-rich *N*-heterocyclic carboxylic acids with relevance to drug discovery projects (**o,** **p,** **q,** **r**). The reactions were miniaturized to 0.5 μmol scale, downsizing by a factor of 300 compared to the literature procedure^16^. To achieve this small reaction scale, stock solutions of all components in the reaction solvent (DMSO) were produced. Consequently, the designed screening plate only requires 4.2 mg of starting material (molar mass: 350 Da) to assess 23 different transformations. In comparison, single reactions in reference^16^ were carried out with 52.5 mg of starting material. Using a substrate from reference^16^ (Molecule **1**, structure depicted in Fig. S2 in Supplementary Note 5), different oxidant to carboxylic acid ratios (3:10, 6:10, 3:20, 6:20) were tested to identify the more favorable screening condition (higher conversion). Further, the impact of other parameters, such as the atmosphere (under air, under nitrogen in a glovebox), and the reaction concentration (2, 16 mmol/L) was investigated. Upon determining the highest-yielding reaction parameters, the best-performing condition on the plate (B4, **1** with **e**, under nitrogen, 16 mmol/L) was used as the reference reaction to monitor reproducibility across different plates. Incorporating the control experiment in position B4, which consistently remained unchanged, served the purpose of swiftly detecting potential handling errors with the plate and confirming the reliability of the generated data. The plate layout including all reaction parameters is shown in Fig. 2. Additional information on the plate testing is provided as supporting information (Supplementary Note 6, Figs. S7–S9).

### LSF informer library

For the generation of the experimental reaction dataset, the previously published informer library was used as a starting point (see ref. ^41^ for details). From this collection, three fragments (**2**–**4**, Fig. S2 in Supplementary Note 5 for structures) and five drug molecules (**5**-**9**, Figure S2 in Supplementary Note 5) were screened. The drug molecule library in ref. ^41^ was assembled based on clustering of 1174 approved small molecule drugs into eight structurally diverse subsets. As three clusters did not contain any reactive functional groups required for Minisci-type reactions (e.g., electron-deficient heterocycle), only five drug molecules (**5**–**9**) were subjected to HTE alkylation screening (see “HTE alkylation screening” for details). The screening of the drugs was extended by three fragments (**2**-**4**) from ref. ^41^. Furthermore, a decoy data set containing 368 unsuccessful reaction examples was generated. The chemical structures of the eight *N*-hetero-arene substrates (**2**-**9**, Fig. S2) as well as the 16 decoy substrates (**10**-**25**, Fig. S3) used to train the machine learning are provided as supporting information (Supplementary Note 5).

To assess the performance, i.e., the prediction accuracy, of the developed machine learning model on relevant fragments for applications in medicinal chemistry, a substructure search for heteroaromatic ring systems containing at least one nitrogen atom was carried out in the Roche corporate compound collection. The resulting compounds were retained if (i) there was at least 1 g of powder stock available, and (ii) the structures were not used in any internal project or subject to legal restrictions. This pool of candidates was then clustered using sphere exclusion clustering^50^ on ECFP4 fingerprints^51^ with a Tanimoto cutoff ^52^ of 0.6. Based on the clustering results, we manually selected 18 structurally diverse fragments (**26**-**43**, Fig. 4, Supplementary Note 2, Fig. S1).

### HTE alkylation screening

Using the 24-well plate design (Fig. 2, Supplementary Note 6), selected drug molecules and fragments from the LSF informer library (**2**-**9**, Supplementary Note 5, Fig. S2), a set of relevant building blocks (**26**-**43**, Fig. 4, for detailed information: Supplementary Note 5, Figs. S4, S5) and substrates from Sutherland et al.^16^ (**44**-**48**, Supplementary Note 5, Fig. S6) were screened. The reaction setup (stock solution, liquid handling) and execution (heating, stirring) in glass vials on a parallel screening plate were conducted in a glovebox under nitrogen. Upon completion of the reactions, the residues were diluted in MeCN/H_2_O to a defined concentration suitable for LCMS analysis, using a liquid handler. The resulting mixtures were analyzed by LCMS, and the results were subjected to automated reaction data analysis (Supplementary Note 8) for the determination of the molecular components. Standardized data output (Supplementary Note 9) allowed for direct visualization of the information in TIBCO Spotfire (Somerville, USA). The general screening procedure, including detailed information on the hardware and software utilized, is provided as Supporting Information (Supplementary Note 7).

### Scale up reactions

Analysis of the screening results revealed that the drugs Loratadine (**7**), Nevirapine (**8**), and 11 fragments (**26,** **28,** **29,** **33**-**35,** **37**-**41**) were alkylated with different types of alkyl fragments. From this subset, conditions showing reasonable conversion (>40%, based on UV trace) were subjected to upscaling. Reactions were conducted under nitrogen in a glovebox, in glass reaction vessels with pressure release caps and standard stirring bars. Purification was performed by flash chromatography or reversed-phase high-pressure liquid chromatography (RP-HPLC). Structural elucidation was performed with NMR spectroscopy and HRMS. All comprehensive experimental details for the scale-up processes, including analytical outcomes and spectra of the purified and fully characterized compounds, can be found in the Supporting Information (Supplementary Note 12 and Supplementary Data 1, Figs. S12–S29).

### Graph neural network architecture

A graph transformer neural network (GTNN) architecture was employed based on the E(3) equivariant graph neural network architecture^53^, which has seen use in several related applications^54,55^. The GTNN was designed using the same training procedure as in reference^41^ and a slightly adapted architecture that allows for two distinct and variable molecular graphs in its input, i.e., *N*-hetero arenes and carboxylic acids (Supplementary Note 1). Furthermore, the initial machine learning framework was extended to allow for prospective screening of individual substrates, carboxylic acids or single reactions. For both molecular graphs, their 3D conformers were calculated using the universal force field method^56^, and the graph was constructed using nodes represented by atoms and edges defined by all neighboring atoms within a radius of 4 Å of each atom.

Atoms were featured using embeddings of four atom-level features:

- 12 atom types (H, C, N, O, F, P, S, Cl, Br, I, Si, Se);
- 2 ring types (True, False);
- 2 aromaticity types (True, False);
- 4 hybridization types (sp^3^, sp^2^, sp, s).

First, the individual atomic embedding was concatenated and transformed into an initial atomic representation ${{{{{{{{\bf{h}}}}}}}}}_{i}^{0}$ via a multi-layer perceptron (MLP). Atomic representations ${{{{{{{{\bf{h}}}}}}}}}_{i}^{0}$ were subsequently transformed via three message-passing layers. In each message-passing layer, the atomic representations were transformed via Eq. (1)

1 $${{{{{{{{\bf{h}}}}}}}}}_{i}^{l+1}=\phi \left({{{{{{{{\bf{h}}}}}}}}}_{i}^{l},\mathop{\sum}\limits_{j\in {{{{{{{\mathcal{N}}}}}}}}(i)}\psi ({{{{{{{{\bf{h}}}}}}}}}_{i}^{l},{{{{{{{{\bf{h}}}}}}}}}_{j}^{l},{{{{{{{{\bf{r}}}}}}}}}_{i,j},)\right),$$

where ${{{{{{{{\bf{h}}}}}}}}}_{i}^{l}$ is the atomic representation of the *i*-th atom at the *l*-th layer; $j\in {{{{{{{\mathcal{N}}}}}}}}(i)$ is the set of neighboring nodes connected via edges; **r**_*i*,*j*_ the inter-atomic distance represented in terms of Fourier features, using a sine- and cosine-based encoding; *ψ* is an MLP transforming node features into message features **m**_*ij*_: ${{{{{{{{\bf{m}}}}}}}}}_{ij}=\psi ({{{{{{{{\bf{h}}}}}}}}}_{i}^{l},{{{{{{{{\bf{h}}}}}}}}}_{j}^{l},{{{{{{{{\bf{r}}}}}}}}}_{i,j})$ for 3D graphs, and ${{{{{{{{\bf{m}}}}}}}}}_{ij}=\psi ({{{{{{{{\bf{h}}}}}}}}}_{i}^{l},{{{{{{{{\bf{h}}}}}}}}}_{j}^{l})$ for 2D graphs; ∑ denotes the permutation-invariant pooling Operator (i.e., sum) transforming **m**_*ij*_ into **m**_*i*_: ${{{{{{{{\bf{m}}}}}}}}}_{i}={\sum }_{j\in {{{{{{{\mathcal{N}}}}}}}}(i)}{{{{{{{{\bf{m}}}}}}}}}_{ij}$; and *ϕ* is an MLP transforming ${{{{{{{{\bf{h}}}}}}}}}_{i}^{l}$ and **m**_*i*_ into ${{{{{{{{\bf{h}}}}}}}}}_{i}^{l+1}$. The resulting atomic features from all layers $[{{{{{{{{\bf{h}}}}}}}}}_{i}^{l = 1},{{{{{{{{\bf{h}}}}}}}}}_{i}^{l = 2},{{{{{{{{\bf{h}}}}}}}}}_{i}^{l = 3}]$ were concatenated and transformed via an MLP, resulting in final atomic features. Atomic features were then pooled via a graph multiset transformer (GMT)^57^ with four attention heads yielding an overall molecular feature vector.

This procedure was conducted for both input molecular graphs, where no weights were shared between the two GNN modules except for the initial embedding layers of atom-level representations. The pooled molecular representations were then concatenated to a learned representation of the reaction conditions (Fig. 3B). This subsequent reaction representation was further transformed via a final MLP converting the latent space to the desired reaction output. Both of the examined problems, namely, reaction yield prediction and binary reaction outcome prediction, were addressed as regression tasks. The output for reaction yield was defined within the range of floating values from 0 to 1, whereas for binary reaction outcomes, it was defined as either 0 or 1.

Consistent with the results outlined in ref. ^41^, the performance of the models was validated for GNNs trained on molecular graphs that included atomic partial charges^58–60^. This evaluation revealed that there was no substantial improvement or decline in model performance. Consequently, for all the applications described, 3D graphs without electronic features were employed (Supplementary Note 3, Tables S1, S2).

### Reaction condition representation

Reaction conditions were represented by one-hot-encoding for molecular entities, i.e., reagents, solvents, catalysts, additives and atmosphere, and by real numbers for scalars, i.e., equivalents for starting materials, reagents, carboxylic acids, catalysts, and additives, fractions for the solvents, temperature (°C), reaction time (h), and scale (mmol/L). The individual conditions were concatenated with each other and transformed via an MLP. This reaction condition representation was then concatenated to the learned representations of the two substrates, i.e., N-hereto arene and carboxylic acid.

### Number of hyperparameters

The feature dimension for the internal representation of GTNN was established at 128, with the exception of the embedding dimension for the reaction and atomic properties, which was set to 64. Additionally, the first MLP layer following the graph multiset transformer-based pooling was configured to have 256 dimensions. The graph multiset transformer employed two attention heads for pooling. These parameter settings translated into neural network sizes with ~2.0 million trainable parameters for GTNN.

### Metric for model validation

For model validation and optimization, mean absolute error was used for reaction yield prediction. For predicting binary reaction outcomes the models were validated using absolute accuracy and the *F*-score metric. The *F*-score (*F*_1_) is used as a measure for unbalanced data sets and is calculated by the mean of precision and recall (Eq. (2)):

2 $${F}_{1}=\frac{2tp}{(2tp+fp+fn)}$$

where *tp* represents true positives, *fp* false positives, and *fn* false negatives.

### Decoy data set

The decoy data set comprised 308 instances of unsuccessful reactions, derived from 16 substrates that lack reactivity under Minisci-type conditions due to the absence of an aromatic or heteroaromatic component in their starting materials. These selected molecules underwent thorough scrutiny by experts and were subsequently incorporated into the data set as instances of negative or unsuccessful reaction outcomes. This inclusion serves to furnish the model with knowledge about molecules that do not exhibit reactivity when subjected to Minisci conditions (Supplementary Note 5, Fig. S3).

### Substrate selection

The selection of a diverse and reactive set of *N*-hetero arenes was based on a Roche-internal library of 3180 advanced heterocyclic building blocks with a molecular weight between 200 and 1000 g/mol. Aiming to check these compounds for potential reactivity in the alkylation reaction, this library was virtually screened with preliminarily trained GNN models. Each of the *N* = 3180 molecules was assigned with an average score value calculated with six independent GNNs (“Machine learning-based in silico reaction screening” for details). Subsequently, agglomerative compound clustering was performed^61^. The molecules were encoded as an *N* × *N* similarity matrix containing pairwise Jaccard similarity values based on ECFP4 molecular fingerprint descriptors^51^. Clustering resulted in eight clusters of which six were used for substrate selection. Three top-scoring compounds were selected for HTE reaction screening for each of the six clusters. This clustering approach was chosen to allow for the selection of chemically diverse reactive substrates.

### In silico reaction screening

For model application, a total of six GNNs were trained. Three models were trained for predicting reaction yield, and three models were trained for binary reaction outcome prediction. These models were then utilized to predict the reaction outcomes and reaction yields for each combination of the 3180 advanced heterocyclic building blocks and the 23 carboxylic acids. The predictions yielded values for both binary reaction outcomes and reaction yields, each ranging from 0 to 1. Given that three models were employed for each of the two prediction values, mean and standard deviations were computed to provide an understanding of the model’s uncertainty. The final score was then determined as the mean of the two predictions. Subsequently, each of the six molecule clusters was ranked based on the calculated score, and molecules from the upper echelons of the list were chosen for further consideration or selection.

### Supplementary information

Supplementary Information

Description of Additional Supplementary Files

Supplementary Data 1

Supplementary Data 2

Supplementary Data 3

Supplementary Data 4

Supplementary Data 5

Supplementary Data 6

Supplementary Data 7

Supplementary Data 8

## Data Availability

The SURF-formatted literature, experimental and decoy data sets containing 45, 691 and 368 reactions, respectively, are enclosed as TSV files as Supplementary Data 2–8. Description of Supplementary Data: Supplementary Data 1: PDF file containing NMR spectra. Supplementary Data 2: TSV file containing all reactions (i.e., literature, decoy and experimental data). Supplementary Data 3: TSV file containing reactions from literature. Supplementary Data 4: TSV file containing experimental reaction data. Supplementary Data 5: TSV file containing reactions conducted to validate the literature data. These reactions were excluded in machine learning model training. Supplementary Data 6: TSV file containing decoy reactions. Supplementary Data 7: TSV file containing all investigated carboxylic acids. Supplementary Data 8: TSV file containing all investigated *N*-hetero arenes.

## Code availability

A reference implementation of the geometric machine learning platform based on PyTorch^62^ and PyTorch Geometric^63^ is available at https://github.com/ETHmodlab/minisci (rep. DOI: 10.5281/zenodo.8344587, https://zenodo.org/record/8344587).
