# Supplementary material for: Identifying opportunities for late-stage C-H alkylation with high-throughput experimentation and in silico reaction screening
Source: Commun Chem. 2023 Nov 20;6:256. doi: 10.1038/s42004-023-01047-5 (PMC10661846; doi:10.1038/s42004-023-01047-5)
Supplement: Supplementary file 1 — Supplementary Information [file 42004_2023_1047_MOESM1_ESM.pdf]

Supplementary Information: Identifying opportunities for late-stage C-H alkylation with *in silico* reaction screening and high-throughput experimentation

David F. Nippa[1,2,†], Kenneth Atz[1,†], Alex T. Müller[1], Jens Wolfard[1], Clemens Isert[3], Martin Binder[1], Oliver Scheidegger[1], David B. Konrad[2,*], Uwe Grether[1,*], Rainer E. Martin[1,*] & Gisbert Schneider[3,*]

[1]Roche Pharma Research and Early Development (pRED), Roche Innovation Center Basel, F. Hoffmann-La Roche Ltd., Grenzacherstrasse 124, 4070 Basel, Switzerland.
[2]Department of Pharmacy, Ludwig-Maximilians-Universität München, Butenandtstrasse 5, 81377 Munich, Germany.
[3]ETH Zurich, Department of Chemistry and Applied Biosciences, Vladimir-Prelog-Weg 4, 8093 Zurich, Switzerland.
[4]ETH Singapore SEC Ltd, 1 CREATE Way, #06-01 CREATE Tower, Singapore, Singapore.
† These authors contributed equally to this work.
∗ To whom correspondence should be addressed.
E-mail: david.konrad@cup.lmu.de, uwe.grether@roche.com, rainer_e.martin@roche.com, gisbert@ethz.ch

Supplementary Note 1 Training Details

PyTorch Geometric (2.0.2) [1] and PyTorch (1.10.1+cu102) [2] functionalities were used for neural network training. Training was performed on a graphical processing unit GPU (Nvidia GeForce GTX 1080 Ti) for four hours, using a batch size of 16 samples. The Adam stochastic gradient descent optimizer was employed [3], with a learning rate of 10^{-4}, mean squared error (MSE) loss on the training set, a decay factor of 0.5 applied after 100 epochs, and an exponential smoothing factor of 0.9. The final model was stored after 1000 epochs. All the models considered in this study were trained on the Euler computing cluster at ETH Zurich, Switzerland.

Supplementary Note 2 Clustering

Figure S1 illustrates the clustered chemical space of 3180 advanced heterocyclic building blocks within principal component analysis (PCA). Exemplary chemical structures at the corners of the scatter plot are highlighted.

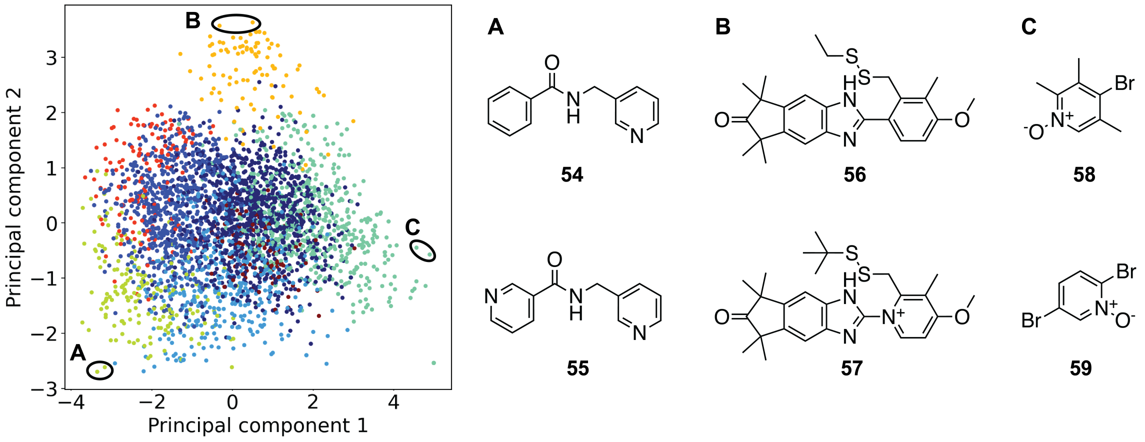

Figure S1: Compound clustering. Principal component analysis (PCA) of the 3180 advanced heterocyclic building blocks, based on ECFP4 molecular fingerprint descriptors. [4] Colors indicate the eight compound groups (clusters) obtained in the clustering process. The explainable variance for the investigated data set of the first two principal components is 22.2% and 9.6%, respectively. **A-C**: Chemical structures from three selected regions of the investigated chemical space. **A**: Amid derivatives (**54**, **55)** populating the bottom left of the PCA plot. **B**: Benzimidazole derivatives **56** and **57** populating the upper center of the PCA plot. **C**: Pyridine oxide derivatives **58** and **59** populating the center right of the PCA plot.

Supplementary Note 3 Steric and electronic graph-features

To validate the influence of electronic features in the input molecular graph on model performance as conducted in a previous study [5], two different GNNs have been trained for reaction yield and binary reaction outcome prediction (Table S1 and S2). The QM features were calculated on-the-fly using the DelFTa software package trained on the QMugs data collection [6–9]. Reaction yields were predicted with an error margin of 18 − 19 % and binary reaction outcome could be learned with an area under the receiver operating characteristic curve (AUC) of 82-83 % (Table S1). Electronic effects have shown significant improvements for the investigated tasks.

Table S1: Neural network performance for reaction yields prediction.

	**PCC**	**MAE / %**
**GNN3D**	0.686 (±0.006)	18.7 (±0.2)
**GNN3DQM**	0.67 (±0.01)	18.6 (±0.6)

Table S2: Neural network performance for binary reaction outcome prediction.

	**Absolute accuracy / %**	***F*-score**
**GNN3D**	80.8 (±1.2)	82.7 (±0.6)
**GNN3DQM**	80.5 (±0.7)	82.5 (±0.2)

Supplementary Note 4 Systematic literature analysis

Following the previously reported systematic analysis of chemical transformations (SACT) concept [5], suitable Minisci-type alkylation reactions were identified. SACT comprises of (1) literature search, (2) literature data curation and evaluation, (3) methodology extraction, (4) reaction data curation and analysis.

The literature search (1) was conducted using three different, renowned tools, Scopus (Elsevier, Amsterdam, Netherlands), Web of Science (Clarivate Analytics, Philadelphia, USA) and SciFinder-n (Chemical Abstracts Service, Columbus, USA) on the 30th of August 2022. On all databases a keyword search for "Minisci reaction" was carried out and the results download.

These files were subjected to a custom-built Alteryx Designer (Irvine, US) data curation (2) workflow that removed duplicates, added information from other databases, *e.g.*, journal impact factor, and carried out further filtering as well as calculations before splitting the publications into four quadrants based on journal impact factor and citations per year. After the removal of duplicates, 114 unique publication records were identified. With the available data, various different clustering approaches could have been carried out using a selection of the following dimensions, *e.g.*, journal and affiliation, citations, journal impact factor, technologies, catalysts, starting materials, and publication year. For this work, clustering by citations per year over journal impact factor to determine the most relevant Minisci methodology publications (high citations/year, high journal impact factor and high citations/year, low journal impact factor) was chosen. Removal of review papers delivered 45 remaining records [10–54], which underwent manual analysis to guarantee that the papers are within the scope of the automated HTE system (*e.g.*, photo- and electrochemistry out of scope). To allow for a broad utilization in medicinal chemistry, the publications were specially screened for a fast, robust and easily adaptable procedure. Among those, only Sutherland and co-workers [41] delivered a Minisci methodology that fulfilled those precise criteria. The metal- and catalyst-free setup as well as the ability to work with multiple non-pre-functionalized alkyl carboxylic allows for customized screening template design depending on project needs.

Next, using the simple user-friendly reaction format (SURF, see Section SI9 for further details), the reaction data from [41] was extracted and curated manually (3). This resulted in a high-quality data set comprising 45 borylation reactions serving as an ideal starting point for the development of the screening plate (see Section SI 6) through data analysis (4). Further, as a SURF file is machine-readable, the data was directly available as input for the machine learning pipelines.

Supplementary Note 5 LSF informer library

LSF informer library (drugs and fragments)

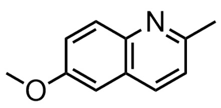

1
(1078-28-0)

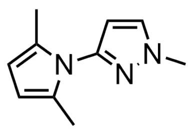

2
(34605-66-8)

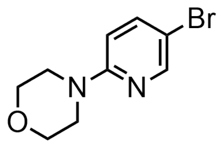

3
(200064-11-5)

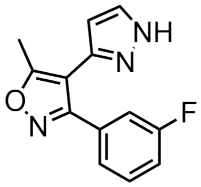

4
(n.d.)

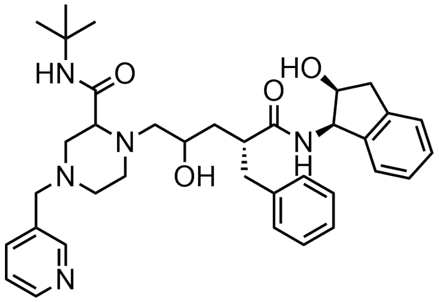

5
(150378-17-9)
Indinavir

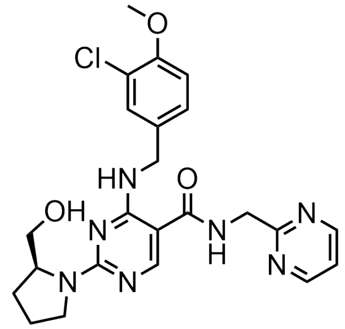

6
(330784-47-9)
Avanafil

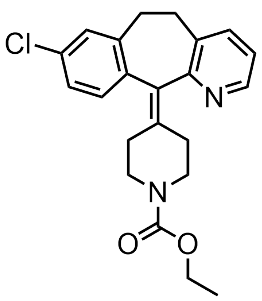

7
(79794-75-5)
Loratadine

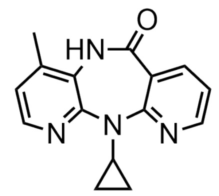

8
(129618-40-2)
Nevirapine

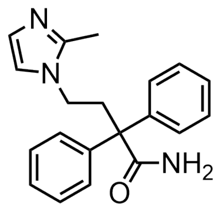

9
(170105-16-5)
Imidafenacin

Figure S2: Fragments and drugs of the LSF informer library **1-9**. For compounds that have a trading name and a CAS number, the identifiers are depicted below the molecule.

Decoy substrates

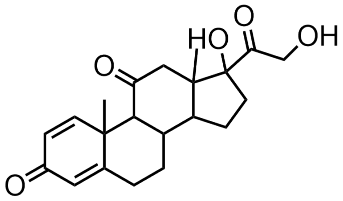

10
(53-03-2)
Prednisone

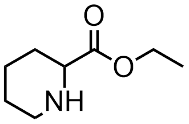

11
(15862-72-3)

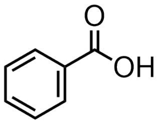

12
(65-85-0)

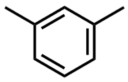

13
(108-38-3)

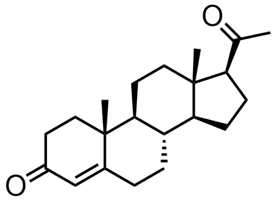

14
(57-83-0)
Progesterone

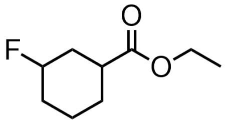

15
(888970-77-2)

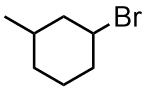

16
(13905-48-1)

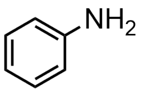

17
(62-53-3)

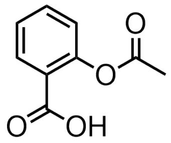

18
(50-78-2)
Aspirin

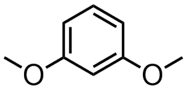

19
(151-10-0)

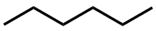

20
(110-54-3)

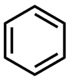

21
(71-43-2)

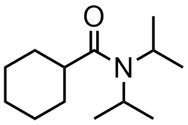

22
(15862-72-3)

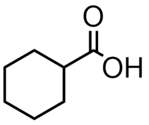

23
(98-89-5)

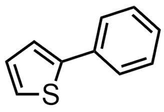

24
(825-55-8)

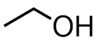

25
(64-17-5)

Figure S3: Selection of decoy substrates **10-25** used to generate unsuccessful reaction data to create a balanced training set. For compounds that have a trading name and a CAS number, the identifiers are depicted below the molecule

Fragments

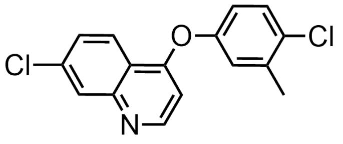

26
(124496-23-7)

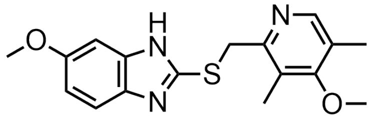

27
(73590-85-9)

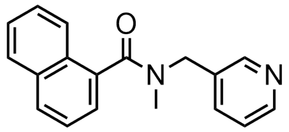

28
(1624057-23-3)

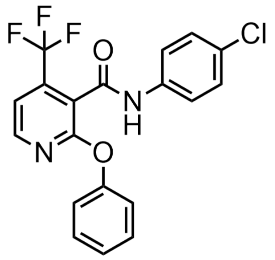

29
(685112-46-3)

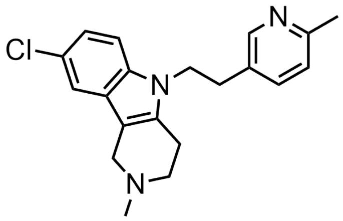

30
(21228-13-7)

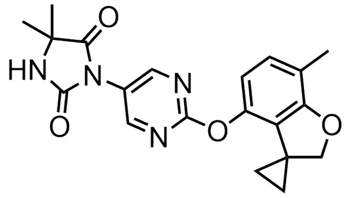

31
(1380696-64-9)

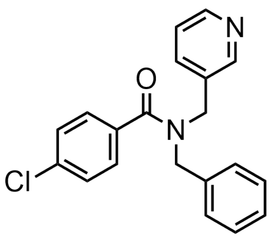

32
(287918-44-9)

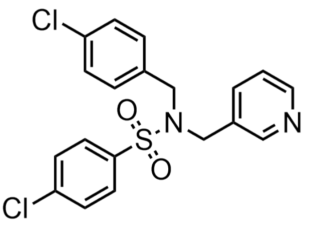

33
(685123-66-4)

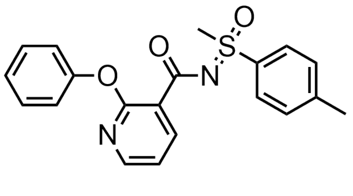

34
(882253-85-2)

Figure S4: Relevant fragments from the Roche library **26-34**. Part 1. For compounds that have a CAS number, the identifier is depicted below the molecule.

Fragments (continued)

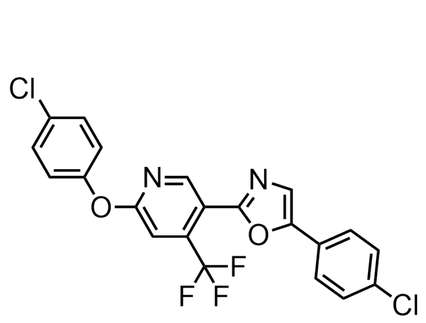

35
(883099-18-1)

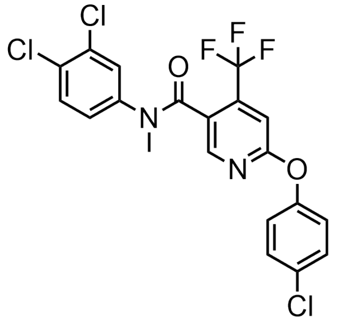

36
(287978-94-3)

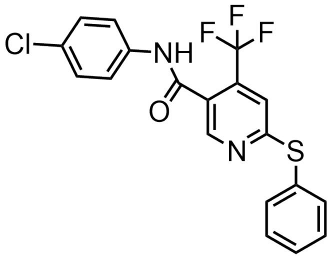

37
(685124-85-0)

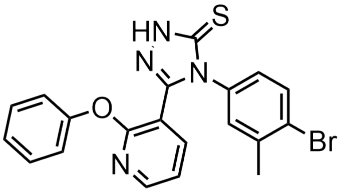

38
(218157-26-7)

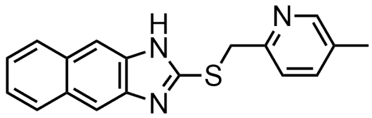

39
(71670-51-4)

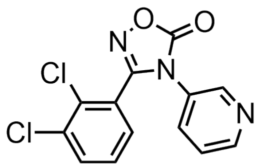

40
(288246-51-5)

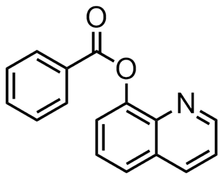

41
(86-75-9)

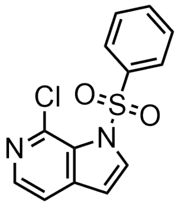

42
(1415124-76-3)

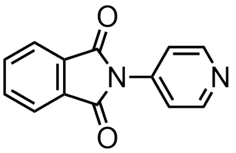

43
(69076-65-9)

Figure S5: Relevant fragments from the Roche library **35-43**. Part 2. For compounds that have a CAS number, the identifier is depicted below the molecule.

Substrates (publication)

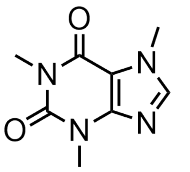

44
(58-08-2)

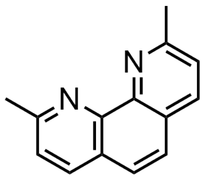

45
(484-11-7)

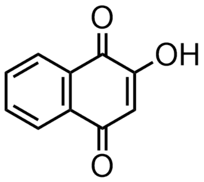

46
(83-72-7)

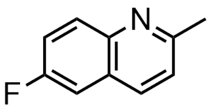

47
(1128-61-6)

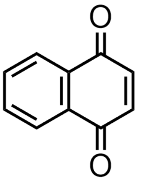

48
(130-15-4)

Figure S6: Selection of substrates from [41], which also underwent screening with the designed plate and showed similar performance. For compounds that have a CAS number, the identifier is depicted below the molecule.

Supplementary Note 6 Screening plate design and testing

As indicated in the main manuscript (Section 4.2), the screening plate was designed around the literature data obtained from *Sutherland et al.* [41], which showed good yields on average (60%) for a variety of carboxylic acid coupling partners. To assess the reactivity of a substrate with a variety of different alkyl rests (rings and chains), a screening plate with 24 different alkyl carboxylic acids was assembled. The carboxylic acids scope from *Sutherland et al.* [41] was extended by sp^3-rich *N*-heterocyclic carboxylic acids with relevance to drug discovery projects (**o**, **p**, **q**, **r**, Figure 2). The reactions were miniaturized to 0.5 µmol scale, downsizing by a factor of 300 compared to the literature procedure. [41] To achieve this small reaction scale, stock solutions of all components in the reaction solvent (DMSO) were produced. Using simple substrate **1**, different oxidant and carboxylic acid ratios (3:10, 6:10, 3:20, 6:20) were tested to identify the more favourable screening condition (higher conversion). Further, the influence of the atmosphere (under air, under nitrogen in a glovebox), and the concentration (2, 16 mmol/L) on the reaction outcome were assessed. The results of this optimization process, which led to the final plate design (Figure 2) are disclosed below.

Air and inert atmosphere

To understand if the reaction requires an air-free atmosphere to deliver good yields, a selection of acid combinations were tested under air and in the glovebox on starting material **1**. The experimental results revealed that carrying out the reaction in the glovebox leads to significantly higher yields compared to the corresponding reaction under air (Figure S7).

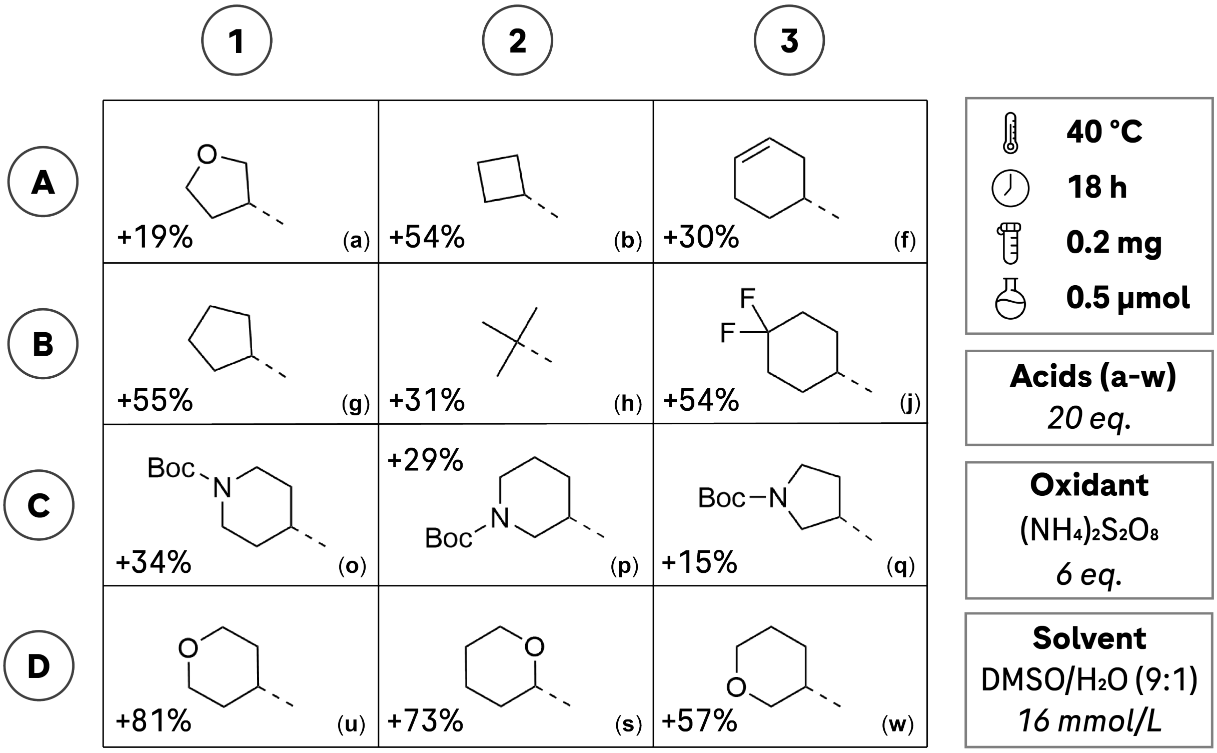

Figure S7: Results from the plate testing with different carboxylic acids (**a**, **b**, **f**, **g**, **h**, **j**, **o**, **p**, **q**, **u**, **s**, **w**) under air and in the glovebox. The yield difference shown in the columns reflects the yield improvements when carrying out the reaction in a glovebox. *Reaction conditions*: Starting material (**1**, 5 umol), oxidant $(NH_4)_2S_2O_8$, solvent $DMSO/H_2O$ (600:1), c = 2 mmol/L, 18 h, 40 °C.

Based on these outcomes, all further screening experiments took place in the glovebox.

Carboxylic acid and oxidant ratio

To identify the influence of the oxidant and carboxylic acid equivalents on the reaction success, four different combinations (3:10, 3:20, 6:10, 6:20) were experimentally tested. For these experiments, three different carboxylic acids (**e**, **j**, **o**) were reacted with starting material **1**. All other parameters were held constant (c = 2 mmol/L, t = 18 h, T = 40 °C, glovebox).

		1	2	3
	Carboxylic Acid / Oxidant : Acid (X : Y eq.)	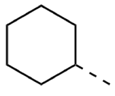 (e)	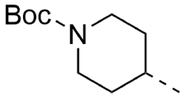 (j)	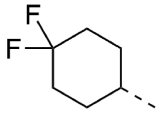 (o)
A	3:10	15%	12%	13%
B	6:10	39%	11%	**38%**
C	3:20	27%	12%	16%
D	6:20	**59%**	**19%**	31%

40 °C
18 h
0.2 mg
0.5 µmol
N_2

Oxidant
$(NH_4)_2S_2O_8$

Solvent
DMSO/H_2O (600:1)
2 mmol/L

Figure S8: Results from plate testing with different oxidant and carboxylic acid equivalents on model substrate **1**. The influence on three different carboxylic acids (**e**, **j**, **o**) was tested. *Reaction conditions*: Starting material (**1**, 5 umol), oxidant $(NH_4)_2S_2O_8$, solvent DMSO/H_2O (600:1), c = 2 mmol/L, 18 h, 40 °C, glovebox.

The results in Figure S8 show that for two of the three alkyl carboxylic acids (**e**, **j**), the 6:20 oxidant to carboxylic acid ratio delivered the best results. Even though for **o**, the 6:10 ratio showed the best results, the performance of 6:20 was in a similar range. Thus, the 6:20 ratio was incorporated into the final plate layout.

Reaction concentration

To avoid requiring additional solvent addition, *i.e.*, only dosing the stock solutions and using their solvent volume, a final comparison test was carried out comparing the influence of the solvent concentration. In the initial experiments, a reaction concentration of 0.002 mol/L was used. This baseline was compared to an increased concentration (0.016 mol/L, factor 8).

Experimental results (Figure S9) revealed that an increased reaction condition yields a higher amount of alkylated products. This experiment concluded the plate testing as all reactions nearly reached full starting material conversion. The final plate layout used for the screening experiments is depicted in Figure 2C of the main manuscript.

Application of reaction conditions to literature substrates

To verify that we did not optimize our conditions for a specific substrate, i.e., compound **1**, five other starting materials (**44-48**, Section SI5, Figure S6) from Sutherland et al. [41] were screened with the optimized conditions on the whole plate (Figure 2). Similar performance as described in the paper was observed for the coupling with carboxylic acid **e**, confirming that the miniaturized plate can be used for a broader range of substrates and carboxylic acids. The detailed screening data is attached as a SURF file as one of the supplementary files of this manuscript.

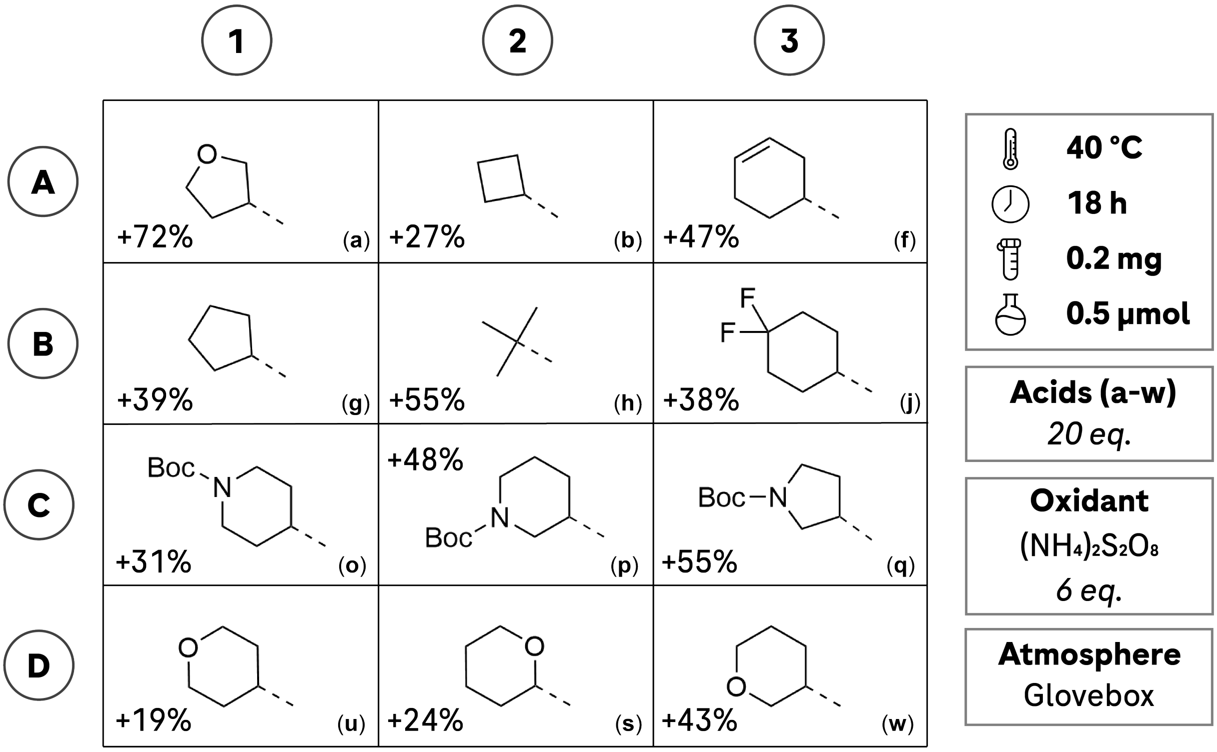

Figure S9: Results from plate testing with two different reaction concentrations on model substrate **1**. The influence of using a slightly higher reaction concentration (0.016 compared to 0.002 mol/L) with different carboxylic acids (**a**, **b**, **f**, **g**, **h**, **j**, **o**, **p**, **q**, **u**, **s**, **w**) was tested The yield difference shown in the columns reflects the yield improvements when carrying out the reaction with higher concentration. *Reaction conditions*: Starting material (**1**, 5 umol), oxidant $(NH_4)_2S_2O_8$, solvent DMSO/H_2O (600:1), c = varied, 18 h, 40 °C, glovebox.

Supplementary Note 7 HTE screening protocol

All generated screening data used the plate design depicted in the paper (Figure 2) and the procedure below, only the starting materials (**1-9** and **26-43**) were varied. In a nitrogen-filled glovebox from mbraun (Garching, DE) that does not contain any liquids, all solid reaction components were dosed into 4 mL glass vials from Analytical Sales (Flanders, US) using a CHRONECT Quantos from Axel Semrau GmbH & Co. KG (Spockhövel, DE) coupled with an XPE206 balance from Mettler Toledo (Greifensee, CH). The vials were sealed and discharged from the glovebox before being transferred to another glovebox from LC Technologies (Salisbury, US), where solvents were added to the vials to generate the stock solutions. The remaining stock solutions with liquid components were prepared in a similar manner. Then according to the plate design, the stock solutions were transferred into 1 mL glass vials from Analytical Sales (Flanders, US) on a 24- or 96-well plate from Analytical Sales (Flanders, US) using multichannel pipettes from Eppendorf (Hamburg, DE). The plate was heated within the glovebox (LC Technologies) on a Junior benchtop solution from Unchained Labs (Pleasanton, US) and VP 721F-1 Parylene Encapsulated Stainless Steel Stir Discs from V&P Scientific Inc. (San Diego, US) were used to stir the reaction mixture. Only one internal process control (IPC) was taken after the overnight reaction by diluting the reaction mixture using a Freedom EVO 100 liquid handler from Tecan (Männedorf, CH) with MeCN/H_2O (4:1) to a defined concentration (1 mmol/L). After shaking on a Teleshake 95 from Inheco (Martinsried, DE), the samples were transferred onto a 96-deep-well plate (1 mL) from Eppendorf (Hamburg, DE). The plates were analyzed on a Waters (Milford, US) UPLC-MS system equipped with a Waters Acquity sample manager with a flow-through needle, a Waters Acquity sample organizer and a Waters QDa single quadrupole mass spectrometer. The separation was achieved on a ZORBAX RRHT Eclipse Plus C18, 95 Å, 2.1 x 30 mm, 1.8 µm column (P/N 959731-902, LOT: USUXY02479) from Agilent (Santa Clara, USA) at 50 °C. A 2-minute gradient was used and the injection volume accounted for 2 µL. 2 min gradient: A: 0.1% HCOOH in H_2O; B: 0.07% HCOOH in MeCN at flow 1 mL/min. Gradient: 0 min, 3% B; 0.2 min, 3% B; 1.5 min, 97% B; 0.3 min, 97% B; 0.1 min 3% B. The raw data were processed with MassLynx V4.2 and the obtained .rpt file underwent parsing with a customized script, before being subjected to the automated reaction data analysis pipeline (Section SI8).

Supplementary Note 8 Automated reaction data analysis pipeline

In general, the same automated reaction data analysis pipeline as described in the SI (Section SI6) in a previous manuscript [5] was used. In this case, the framework was applied to rapidly identify if drugs or fragments were alkylated or not. Therefore, the MS searched for the sum formulas of the desired products (mono-, di- and trialkylated products), which were generated hands-free using a customized script based on the substrate chemical formula.

In addition to the reaction mixtures, all starting materials and, if available, reference products using the same solvent mixture (MeCN:H_2O, 4:1) are measured on the LCMS to obtain the retention time (LC) and mass pattern (MS). This data is stored in a database and needed for the initial two steps of the matching process. More relevant for LSF though, are the desired/potential products of the reaction. Those masses and chemical formulas are calculated based on the starting material information and the transformation. This Alteryx workflow allows hands-free generation of the potential products including molecular weight, mono-isotopic mass and chemical formula (Hill notation). In addition to being used for the reaction data analysis, this data is also the foundation for generating the LCMS input file.

Once the reaction data has passed through the cleaning process, it is compared to the LCMS information from the above-mentioned data sets, starting off with the identification of the starting material. If a trace from the reaction mixture matches the retention time ($\pm$ 0.02 min) and the mass pattern (chemical formula detected, mass channel match with database reference), it receives the starting material tag. The remaining data is then compared to the products that could potentially be formed and are desired (mono-, di- or trialkylated species). Since the exact position of the new functional groups is not known, no reference compounds are available. Therefore, only the five most abundant masses per peak are used for tagging and compared to information from the potential product database. Based on the abundance of the mass and if the chemical formula was found by the LCMS, the tag is complemented by an MS reliability score. The score is higher if the chemical formula was found and the correct mass of the desired product ($\pm$ 0.5 Da) appears in a more abundant channel. For this study, only high MS reliability scores were subjected to the machine learning platform. Last, the unmatched data is classified as unidentified products, and the mass differences between the peak and parent material are calculated to avoid manual calculating of mass differences.

After the tagging is completed, the data streams are recombined and subjected to calculations in order to quantify the reaction components from starting material through reagents to products. To do so, the sum of all LC peaks (integral) is calculated and each peak is then divided by this value. This gives a quantitative measure of the product distribution within the sample, an LCMS conversion. While there are numerous approaches to using internal standards or assays, due to the nature of LSF they have not been applied. LSF reactions tap into new, unexplored chemical space and generally, multiple different components are formed. Therefore, selecting an internal standard that does not overlap with one of these unknown components, is highly difficult.

Upon completion of the calculations, using the identifiers mentioned earlier, reaction information, such as conditions and components, are added to the components that have been identified and quantified. This follows the FAIR data principle and generates a curated, high-quality LSF screening data set that can be stored and shared in the SURF convention (Section SI9). This allows rapid subjecting of the data to machine learning algorithms as done in this research. It also enables direct visualization of the data in known interfaces, such as TIBCO Spotfire (Somerville, USA) or Tableau (Seattle, USA). Using this workflow, the data curation of one plate usually takes less than one minute.

Supplementary Note 9 SURF convention

The simple user-friendly reaction format (SURF) aims at standardizing reaction data reporting through a simple, yet comprehensive and structured format that is usable with a basic understanding of a spreadsheet. SURF does not require any coding experience, advanced IT skills or a web interface. It enables every chemist within or outside the lab to document chemical synthesis in a machine-readable and shareable format. SURF allowed extraction and documentation of the alkylation reactions from literature faster. The generated reaction screening data were also transformed into SURF before being directly subjected to the machine learning pipelines. Reaction documentation following SURF can be implemented in every spreadsheet as the only requirement is the existence of rows and columns.

Each row of the spreadsheet represents the information and data for one single reaction. The SURF convention contains constant (CC) and flexible (FC) categories. CCs never change and are always present, independent of the number of reaction components. They capture the origin and ids of the reaction as well as basic characteristics (reaction type, named reaction, reaction technology) and conditions (temperature, time, atmosphere, scale, concentration, stirring/shaking). Add-ons, such as the procedure or comments, belong to the CCs, too. The FCs describe the more variable part of a reaction, the starting material(s), solvent(s), reagent(s) and product(s). Two identifier options (CAS and SMILES) are available for each component. While the SMILES string is available for every compound and serves as structural input for machine learning models, the CAS number, even though not always available, can be handy for chemists in the lab to order, itemize and find chemicals. For the starting material(s) and reagent(s), *e.g.*, catalyst, ligand, additive, the number and type of columns remain the same (CAS, Smiles, equivalents). If multiple starting materials or reagents are used, additional columns are required. In that case, the three information columns are duplicated and the X is replaced by a number, starting from 1 for the first component, 2 for the second, etc. The same accounts for multiple solvents or products, however, due to their role, they possess more and partly different columns. While the CAS number and/or the SMILES string remain as an identifier, the solvent fraction (in decimals) instead of equivalents is recorded. This allows exact determination of the ratio between solvents. The product category withholds the largest amount of headers as SURF records the yield (in percent), but also the yield type (*e.g.*, isolated, lcms, gcms) as well as the detected mass by MS and the ^{1}H NMR sequence in addition to the common identifiers CAS and Smiles. This not only allows rapid comparison when experiments are reproduced but can also deliver important increments for machine learning models by differentiating between yield types. As most electronic lab journals already record the above-mentioned parameters, by enforcing of documentation compliance combined with simple automated data extraction and cleaning pipelines, numerous reaction data could be accessible in the SURF convention, and readily available for machine learning applications. We spent thoughts on how to further reduce complexity by introducing specific SURFs without FCs for chemical transformations where the reaction components are generally the same. An excellent example would be Suzuki-Miyaura couplings that utilize a set of six to seven components (organoboron species, halide, catalyst, ligand, base, solvents). [55, 56] However, generating different tailored templates would ultimately end up in various different formats and mismatching headers falling short of the main SURF goal to standardize reaction documentation.

The results of this paper would have not been achieved without FAIR data handling using SURF. The manually extracted reaction data (45 reactions from one publication), which were used in this manuscript for data analysis and selectivity prediction, reported in SURF are attached to the SI as a tab-delimited text file. Further, the reaction data of the scale-up experiments in SURF is also added to the SI of this manuscript and significantly increased the efficiency when compiling the experimental part (Section SI12). Moreover, two empty SURF templates are attached as tab-delimited text files: The first file contains the general SURF template, which can be adjusted by introducing additional columns depending on the reaction specifics. The second file is a customized SURF template that should accommodate the vast of chemical transformations: It contains columns for two starting materials, two reagents, one catalyst, one ligand, one additive, two solvents and two products.

Supplementary Note 10 Screening results of acids and fragments

For the investigated carboxylic acids (Figure S10) as well as for the *N*-hetero arenes (Figure S11) different average reaction yields were observed. Figure S10 illustrates the observed reaction yields for 22 carboxylic acids (**a-w**). Carboxylic acids substituted with cyclic ethers (*e.g.*, **u**, **s**, **a**) and alkanes (*e.g.*, **b**, **e**, **g**) were observed with high reaction yields, whereas cyclic boc-protected amines (*e.g.*, **o**, **p**, **q**, **r**) and amides (**d**) resulted in low yields of the respective desired reaction products. Figure S11 illustrates the observed reaction yields for 27 *N*-hetero arenes (**1-9** and **26-43**). Substituted pyridines (*e.g.*, **30**, **31**, **36**, **39**; see Section SI5) were observed with lower yields compared to compounds lacking meta-substituents (*e.g.*, **26**, **32**, **38**, **41**).

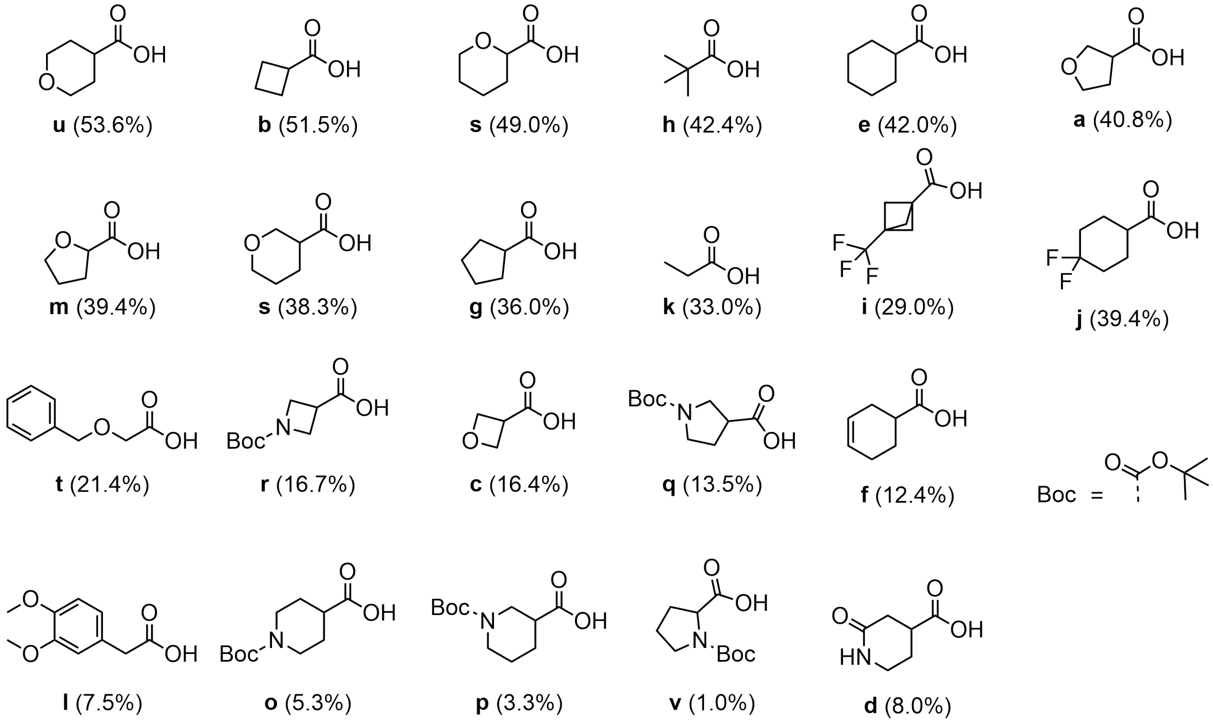

Figure S10: Analysis of screening results for acids.

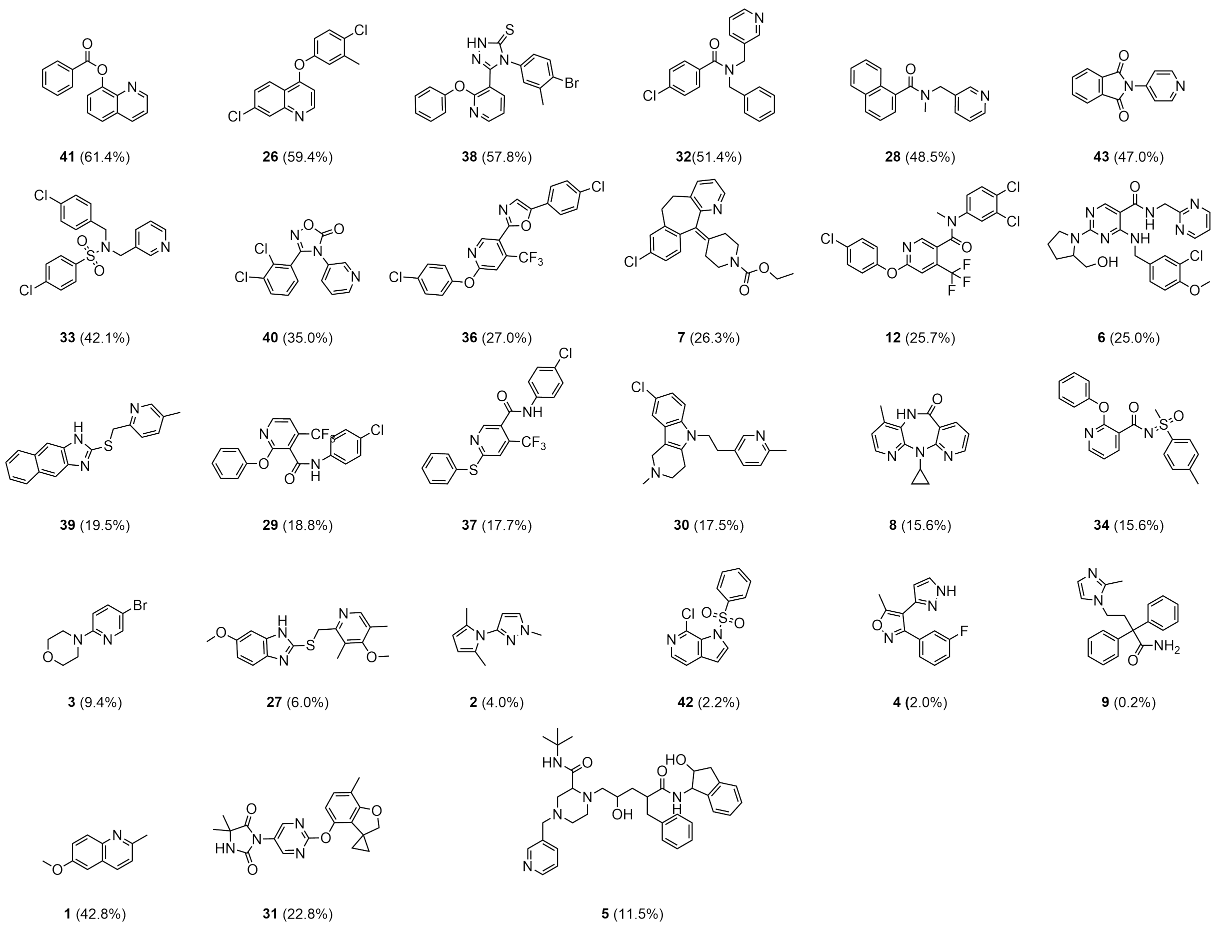

Figure S11: Analysis of screening results for substrates.

Supplementary Note 11 Machine learning outliers

The observed outliers (17/1148) for reaction yield prediction with a mean absolute error (MAE) ≥ 70% are illustrated in Table S3.

Table S3: Machine learning outliers for reaction yield prediction.

**Reaction ID**	MAE (%)	Ground truth (%)
eln044720-053-1-1-b1	82.1	0.0
eln044720-053-1-1-b2	100.0	0.0
eln036496-183-15-1-b6	81.9	3.9
eln036496-188-2-1-d3	73.0	75.9
eln046486-037-1-1-a2	70.8	0.0
eln044720-053-1-1-a2	82.3	0.0
eln036496-183-15-1-b3	71.2	99.0
eln044720-053-1-1-a5	70.3	0.0
eln044720-045-1-1-d2	71.9	87.0
eln036496-191-1-1-d2	73.7	75.0
eln046486-035-1-1-a1	70.5	12.9
eln046486-037-1-1-a1	80.5	88.9
eln044720-053-1-1-c2	75.5	95.9
eln046486-035-1-1-a2	78.1	0.0
eln044720-041-1-1-d2	91.7	0.0
eln044720-053-1-1-a1	72.7	97.0
eln044720-041-1-1-c2	97.6	0.0

The outlier data does not indicate any clear trends, where the model did not perform well. However, selected outliers are discussed below. The two most occurring acids (3x) are A1 (**a**) and D2 (**s**). For **s**, the oxygen in proximity to the reactive site could have led to increased difficulties when predicting the outcome of the transformation as the results for this acid vary broadly across the data set, which is already visible based on the three data points in the table. While the coupling of **s** worked well with **1** and **5**, it was not reactive at all with **38**. For **a**, a conversion could always be observed experimentally, however, the values for the three substrates (**35**, **45**, **46**) differ largely as well. Discrepancies of **45** and **46** might originate due to the new chemical classes (quinones), which are not broadly represented in the data set. **35** generated difficulties for the model (six appearances in the table) as it showed varying experimental behaviour depending on the paired acid. The complexity of the structure including various functional groups with different demanding electronic and steric effects around the pyridine might have led to the observed high MAEs.

Supplementary Note 12 Scale-up reactions

Supplementary Note 12.1 Reagent and purification information

Reactions were set up and conducted in nitrogen-filled gloveboxes from mbraun (Garching, DE) and LC Technologies (Salisbury, US). All chemicals were purchased from Sigma Aldrich (St. Louis, US), AstaTech (Bristol, US), Combi-Blocks (San Diego, US), TRC (Toronto, CA), Thermo Scientific (Waltham, US) or obtained from the Roche compound library and used as received. All solids were dosed using a CHRONECT Quantos from Axel Semrau GmbH & Co. KG (Spockhövel, DE) coupled with an XPE206 balance from Mettler Toledo (Greifensee, CH). Anhydrous solvents were purchased from Sigma Aldrich, stored in the glovebox and added to the reaction vials using pipettes from Eppendorf (Hamburg, DE). The vials were heated on a Junior benchtop solution from Unchained Labs (Pleasanton, US) and the reaction mixture was stirred by VP 721F-1 Parylene Encapsulated Stainless Steel Stir Discs from V&P Scientific Inc. (San Diego, US). Purification by flash column chromatography was performed using SiliaSep Premium Flash Cartridges from Silicycle (Quebec, CA) on a Combi Flash Rf from Teledyne ISCO (Nebraska, US) or by reversed-phase high-pressure liquid chromatography (RP-HPLC) on a Gilson (Middleton, USA) GX-281 liquid handler equipped with a Shimadzu (Kyoto, JP) LC-20AP dual pump, a Thermo Fisher Scientific (Waltham, US) UV/VIS-Thermo Ultimate 300 Detector, a VWR (Radnor, US) ELSD90 ELSD detector and a Thermo Fisher Scientific (Waltham, US) Thermo MSQ Plus MS Single Quadrupole using a Phenomenex (Torrance, US) Gemini NX C18 column (12 nm, 5 um silica, 30 mm diameter, 100 mm length, flow rate of 40 mL/min) or YMC (Kyoto, JP) Triart C18 (12 nm, 5 um, 100x30 mm) column. The used eluent solvents, gradients and cartridge sizes for flash chromatography and RP-HPLC are described individually for each experiment.

Supplementary Note 12.2 Analytical information

All compounds were characterized by nuclear magnetic resonance (NMR) spectroscopy and (flow injection analysis (FIA)) high-resolution mass spectrometry (HRMS) or gas-chromatography mass spectrometry (GCMS). NMR spectra were recorded on a Bruker Avance III, 600 MHz spectrometer equipped with a 5 mm TCI, Z-gradient CryoProbe, a Bruker Avance Neo, 400 MHz spectrometer equipped with a 5 mm Z-gradient iProbe or a Bruker Avance III HD, 300 MHz spectrometer equipped with a 5 mm BBI-Probe. NMR data are reported as follows: chemical shift in reference to the residual solvent peak (δ ppm), multiplicity (s = singlet, d = doublet, dd = doublet of doublet, t = triplet, dt = doublet of triplet, td = triplet of doublet, q = quintet, m = multiplet), coupling constant (Hz), and integration. ^{1}H NMR residual solvent peaks in respective deuterated solvents for $CHCl_3$ at 7.26 ppm and DMSO at 2.50 ppm. ^{13}C NMR residual solvent peaks in respective deuterated solvents for $CHCl_3$ at 77.16 ppm and DMSO at 39.52 ppm.

LC-MS high-resolution spectra were recorded with an Agilent LC system consisting of Agilent 1290 high-pressure gradient system, and an Agilent 6545 QTOF. The separation was achieved on a Zorbax Eclipse Plus C18 1.7 µm 2.1 x 50 mm column (P/N 959731-902) at 55 °C; A: 0.01% HCOOH in H_2O; B: MeCN at flow 0.8 mL/min. Gradient: 0 min 5% B, 0.3 min 5% B, 4.5 min 99% B, 5 min 99% B. The injection volume was 2 µL. Ionization was performed in an Agilent Multimode source. The mass spectrometer was run in "2 GHz extended dynamic range" mode, resulting in a resolution of about 20 000 at m/z = 922. Mass accuracy was ensured by internal drift correction. GC-MS spectra were recorded on an Agilent 5975B single quadrupole mass spectrometer. Separation was achieved on an Agilent 7890A using a HP-1ms column (15 m ID: 250 µm and 0.25 µm film) with He as carrier gas. Sample introduction was done via a Split injector at 270°C. After 0.5 min at a constant temperature, the temperature was ramped from 100 °C or 45 °C to 320 °C with 35 °C/min. The mass spectrometer was operated in EI (electron ionization) mode at 70 eV. FIA-HRMS spectra were recorded with an Agilent LC system consisting of an Agilent 1290 high-pressure gradient system, and an Agilent 6540 QTOF. No separation was intended and the injected sample was flushed directly into the Agilent Jetstream source. The mass spectrometer was run in "2 GHz extended dynamic range" mode, resulting in a resolution of about 20 000 at m/z 922. Mass accuracy was ensured by internal drift correction.

Supplementary Note 12.3 Experimental procedures and analytical data

4-(13-chloro-5-cyclobutyl-4-azatricyclo[9.4.0.03,8]pentadeca-1(15),3,5,7,11,13-hexaen-2-ylidene)piperidine-1-carboxylic acid ethyl ester (7b1),
4-(13-chloro-7-cyclobutyl-4-azatricyclo[9.4.0.03,8]pentadeca-1(15),3,5,7,11,13-hexaen-2-ylidene)piperidine-1-carboxylic acid ethyl ester (7b2),
4-[13-chloro-5,7-di(cyclobutyl)-4-azatricyclo[9.4.0.03,8]pentadeca-1(15),3,5,7,11,13-hexaen-2-ylidene]-piperidine-1-carboxylic acid ethyl ester (7b3):

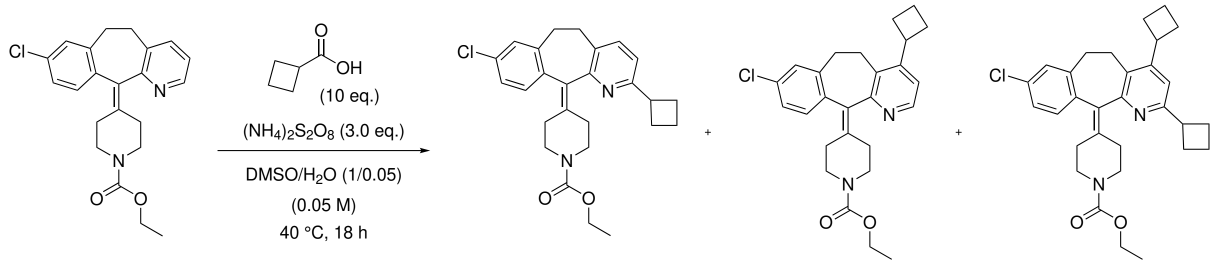

7 **7b1** (9%) **7b2** (4%) **7b3** (4%)

Figure S12: Alkylation of Loratadine (**7**).

To a solution of 4-(13-chloro-4-azatricyclo[9.4.0.03,8]pentadeca-1(11),3(8),4,6,12,14-hexaen-2-ylidene)piperidine-1-carboxylic acid ethyl ester (**7**, 57.4 mg, 0.15 mmol, 1.00 eq.) in 3 mL degassed DMSO and 5 uL H_2O, cyclobutanecarboxylic acid (**b**, 150.2 mg, 1.50 mmol, 10.0 eq.) and ammonium persulfate (102.7 mg, 450 umol, 3.00 eq.) was added. The reaction mixture was degassed while bubbling nitrogen through it. The reaction mixture was stirred at 40 °C for 18 hr. The reaction mixture was quenched with $NaHCO_3$ solution and extracted with DCM. The combined organic layers were washed with water, and brine, dried over Na_2SO_4, filtered and concentrated to dryness. The crude material was purified by reversed-phase HPLC (Gemini NX, 12 nm, 5 um, 100 x 30 mm) using a MeCN gradient (20-40-55%) in H_2O + 0.1% HCOOH. The solvent was removed from product containing fractions. Evaporation of solvents gave the title compounds 4-(13-chloro-5-cyclobutyl-4-azatricyclo[9.4.0.03,8]pentadeca-1(15),3,5,7,11,13-hexaen-2-ylidene)piperidine-1-carboxylic acid ethyl ester (**7b1**, 6.3 mg, 9%) as an off-white powder, 4-(13-chloro-7-cyclobutyl-4-azatricyclo[9.4.0.03,8]pentadeca-1(15),3,5,7,11,13-hexaen-2-ylidene)piperidine-1-carboxylic acid ethyl ester (**7b2**, 2.7 mg, 4%) as an off-white powder and 4-[13-chloro-5,7-di-(cyclobutyl)-4-azatricyclo[9.4.0.03,8]pentadeca-1(15),3,5,7,11,13-hexaen-2-ylidene]piperidine-1-carboxylic acid ethyl ester (**7b3**, 3.8 mg, 4%) as an off-white powder.

7b1:
^{1}H NMR (600 MHz, $CDCl_3$) δ (ppm) 7.34 (d, J = 7.9 Hz, 1H), 7.12 - 7.18 (m, 3H), 7.01 (d, J = 7.9 Hz, 1H), 4.15 (q, J = 7.1 Hz, 2H), 3.76 - 3.81 (m, 1H), 3.60 - 3.66 (m, 1H), 3.35 - 3.40 (m, 1H), 3.26 - 3.31 (m, 1H), 3.19 - 3.24 (m, 2H), 2.75 - 2.85 (m, 2H), 2.53 - 2.56 (m, 1H), 2.30 - 2.38 (m, 6H), 2.20 - 2.30 (m, 2H), 2.00 - 2.06 (m, 1H), 1.85 - 1.89 (m, 1H), 1.27 (t, J = 7.1 Hz, 3H). **^{13}C NMR (151 MHz, $CDCl_3$)** δ (ppm) 161.96, 155.75, 140.18, 138.04, 137.48, 134.69, 132.91, 130.76, 130.28, 128.92, 126.19, 119.22, 61.51, 45.17, 42.16, 31.97, 31.68, 31.15, 30.87, 29.04, 28.73, 18.48, 14.92, 14.89. **HRMS** $C_{26}H_{29}ClN_2O_2$; calc. for (M+H$^+$): 437.1918, found: 437.2.

7b2:
^{1}H NMR (600 MHz, $CDCl_3$) δ (ppm) 8.36 (d, J = 5.1 Hz, 1H), 7.12 (s, 3H), 7.08 (d, J = 4.9 Hz, 1H), 4.15 (q, J = 7.2 Hz, 2H), 3.75 - 3.85 (m, 2H), 3.61 - 3.68 (m, 1H), 3.34 - 3.40 (m, 1H), 3.11 - 3.19 (m, 3H), 2.77 - 2.87 (m, 2H), 2.35 - 2.44 (m, 5H), 2.21 - 2.24 (m, 2H), 2.07 - 2.11 (m, 2H), 1.85 - 1.90 (m, 1H), 1.26 (t, J = 7.1 Hz, 3H). **^{13}C NMR (151 MHz, $CDCl_3$** δ (ppm) 158.25, 155.71, 152.61, 146.89, 139.42, 136.99, 136.81, 134.78,

133.01, 131.34, 131.15, 129.53, 126.19, 120.12, 61.52, 44.99, 44.88, 37.91, 31.91, 30.80, 30.80, 29.45, 28.30, 26.95, 18.57, 14.89. **HRMS** $C_{26}H_{29}ClN_2O_2$; calc. for $(M+H^+)$: 437.1918, found: 437.2.

7b3:

^{1}H NMR (600 MHz, $CDCl_3$) δ (ppm) 7.17 (d, J = 8.1 Hz, 1H), 7.10 - 7.12 (m, 2H), 6.98 (s, 1H), 4.15 (q, J = 7.2 Hz, 2H), 3.75 - 3.86 (m, 2H), 3.58 - 3.66 (m, 2H), 3.33 - 3.38 (m, 1H), 3.08 - 3.21 (m, 3H), 2.74 - 2.82 (m, 2H), 2.32 - 2.38 (m, 6H), 2.24 - 2.26 (m, 3H), 1.99 - 2.12 (m, 3H), 1.85 - 1.89 (m, 2H), 1.26 (t, J = 7.1 Hz, 3H). **HRMS** $C_{30}H_{35}ClN_2O_2$; calc. for $(M+H^+)$: 491.2387, found: 491.2.

4-[5-(benzoxymethyl)-13-chloro-4-azatricyclo[9.4.0.03,8]pentadeca-1(15),3,5,7,11,13-hexaen-2-ylidene]-piperidine-1-carboxylic acid ethyl ester (7t1),
4-[7-(benzoxymethyl)-13-chloro-4-azatricyclo[9.4.0.03,8]pentadeca-1(15),3,5,7,11,13-hexaen-2-ylidene]-piperidine-1-carboxylic acid ethyl ester (7t2)

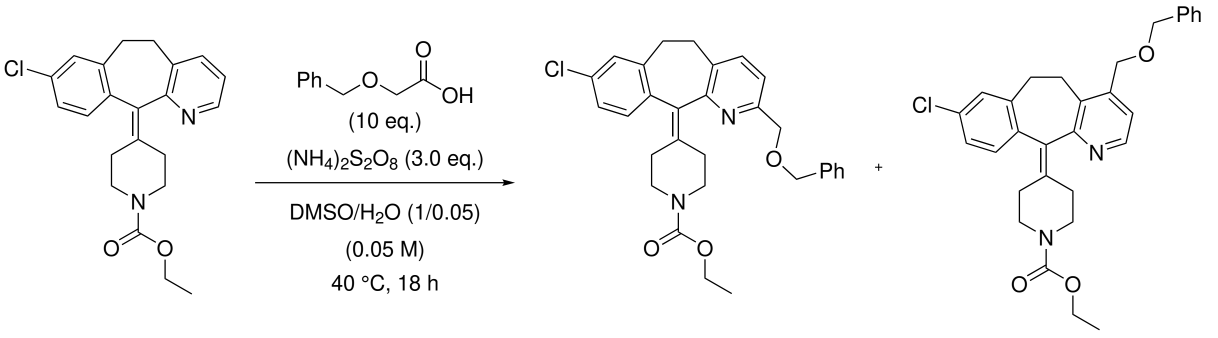

7 **7t1** (10%) **7t2** (9%)

Figure S13: Alkylation of Loratadine (**7**).

To a solution of 4-(13-chloro-4-azatricyclo[9.4.0.03,8]pentadeca-1(11),3(8),4,6,12,14-hexaen-2-ylidene)piperidine-1-carboxylic acid ethyl ester (**7**, 57.4 mg, 0.15 mmol, 1.00 eq.) in 3 mL degassed DMSO and 5 uL H_2O, 2-benzoxyacetic acid (**t**, 249.3 mg, 1.50 mmol, 10.0 eq.) and ammonium persulfate (102.7 mg, 450 umol, 3.00 eq.) was added. The reaction mixture was degassed while bubbling nitrogen through it. The reaction mixture was stirred at 40 °C for 18 hr. The reaction mixture was quenched with $NaHCO_3$ solution and extracted with DCM. The combined organic layers were washed with water, and brine, dried over Na_2SO_4, filtered and concentrated to dryness. The crude material was purified by reversed-phase HPLC (Gemini NX, 12 nm, 5 um, 100 x 30 mm) using a MeCN gradient (55-75-90-100%) in H_2O + 0.1% HCOOH. The solvent was removed from product containing fractions. Evaporation of solvents gave the title compounds 4-[5-(benzoxymethyl)-13-chloro-4-azatricyclo[9.4.0.03,8]pentadeca-1(15),3,5,7,11,13-hexaen-2-ylidene]piperidine-1-carboxylic acid ethyl ester (**7t1**, 8.1 mg, 10%) as an off-white powder and 4-[7-(benzoxymethyl)-13-chloro-4-azatricyclo[9.4.0.03,8]pentadeca-1(15)-,3,5,7,11,13-hexaen-2-ylidene]piperidine-1-carboxylic acid ethyl ester (**7t2**, 6.6 mg, 9%) as an off-white powder.

7t1:
^{1}H NMR (600 MHz, $CDCl_3$) δ (ppm) 7.47 (d, J = 8.0 Hz, 1H), 7.35 - 7.38 (m, 4H), 7.33 - 7.34 (m, 1H), 7.29 - 7.31 (m, 1H), 7.16 - 7.17 (m, 1H), 7.14 - 7.15 (m, 2H), 4.66 (d, J = 2.4 Hz, 2H), 4.63 (s, 2H), 4.15 (q, J = 7.1 Hz, 2H), 3.84 - 3.86 (m, 2H), 3.37 - 3.41 (m, 1H), 3.31 - 3.36 (m, 1H), 3.03 - 3.10 (m, 2H), 2.78 - 2.87 (m, 2H), 2.42 - 2.47 (m, 1H), 2.31 - 2.36 (m, 3H), 1.26 (t, J = 7.1 Hz, 3H). **HRMS** $C_{30}H_{31}ClN_2O_3$; calc. for (M+H$^+$): 503.2023, found: 503.2.

7t2:
^{1}H NMR (600 MHz, $CDCl_3$) δ (ppm) 8.42 (d, J = 5.0 Hz, 1H), 7.38 - 7.40 (m, 1H), 7.35 - 7.38 (m, 3H), 7.32 - 7.35 (m, 1H), 7.30 (d, J = 5.0 Hz, 1H), 7.11 - 7.15 (m, 3H), 4.62 (s, 2H), 4.52 (s, 2H), 4.15 (q, J = 7.1 Hz, 2H), 3.81 - 3.82 (m, 2H), 3.36 - 3.31 (m, 1H), 3.12 - 3.19 (m, 3H), 2.78 - 2.86 (m, 2H), 2.44 - 2.46 (m, 1H), 2.33 - 2.41 (m, 2H), 2.26 - 2.29 (m, 1H), 1.26 (t, J = 7.1 Hz, 3 H). **HRMS** $C_{30}H_{31}ClN_2O_3$; calc. for (M+H$^+$): 503.2023, found: 503.2.

4-(13-chloro-7-cyclohexyl-4-azatricyclo[9.4.0.03,8]pentadeca-1(15),3,5,7,11,13-hexaen-2-ylidene)piperidine-1-carboxylic acid ethyl ester (7e1),
4-(13-chloro-5-cyclohexyl-4-azatricyclo[9.4.0.03,8]pentadeca-1(15),3,5,7,11,13-hexaen-2-ylidene)piperidine-1-carboxylic acid ethyl ester (7e2)

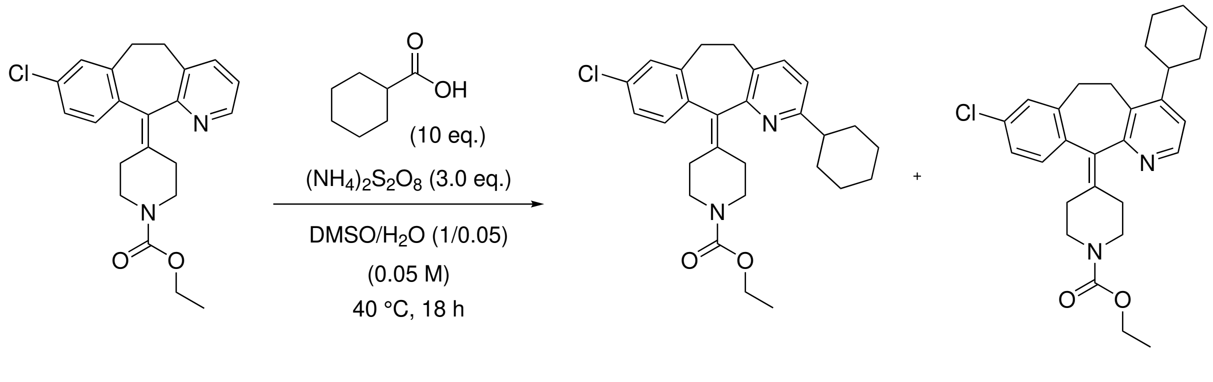

7 **7e1** (11%) **7e2** (5%)

Figure S14: Alkylation of Loratadine (**7**).

To a solution of 4-(13-chloro-4-azatricyclo[9.4.0.03,8]pentadeca-1(11),3(8),4,6,12,14-hexaen-2-ylidene)piperidine-1-carboxylic acid ethyl ester (**7**, 57.4 mg, 0.15 mmol, 1.00 eq.) in 3 mL degassed DMSO and 5 uL H_2O, cyclohexanecarboxylic acid (**e**, 192.3 mg, 186.7 uL, 1.50 mmol, 10.0 eq.) and ammonium persulfate (102.7 mg, 450 umol, 3.00 eq.) was added. The reaction mixture was degassed while bubbling nitrogen through it. The reaction mixture was stirred at 40 °C for 18 hr. The reaction mixture was quenched with $NaHCO_3$ solution and extracted with DCM. The combined organic layers were washed with water, and brine, dried over Na_2SO_4, filtered and concentrated to dryness. The crude material was purified by reversed-phase HPLC (YMC-Triart C18, 12 nm, 5 um, 100 x 30 mm) using a MeCN gradient (20-98%) in H_2O + 0.1% HCOOH. The solvent was removed from product containing fractions. Evaporation of solvents gave the title compounds 4-(13-chloro-7-cyclohexyl-4-azatricyclo[9.4.0.03,8]pentadeca-1(15),3,5,7,11,13-hexaen-2-ylidene)piperidine-1-carboxylic acid ethyl ester (**7e1**, 8.1 mg, 11%) as an off-white powder and 4-(13-chloro-5-cyclohexyl-4-azatricyclo[9.4.0.03,8]pentadeca-1(15),3,5,7,11,13-hexaen-2-ylidene)piperidine-1-carboxylic acid ethyl ester (**7t2**, 5.4 mg, 5%) as an off-white powder.

7e1:
^{1}H NMR (600 MHz, $CDCl_3$) δ (ppm) 7.34 - 7.35 (m, 1H), 7.17 - 7.18 (m, 2H), 7.14 - 7.15 (m, 1H), 6.97 (d, J = 7.8 Hz, 1H), 4.15 (q, J = 7.1 Hz, 2H), 3.82 - 3.87 (m, 2H), 3.35 - 3.40 (m, 1H), 3.27 - 3.31 (m, 1H), 3.09 - 3.13 (m, 1H), 2.68 - 2.84 (m, 1H), 2.33 - 2.36 (m, 3H), 1-94 - 1.97 (m, 1H), 1.88 - 1.90 (m, 1H), 1.73 - 1.76 (m, 1H), 1.38 - 1.49 (m, 6H), 1.27 (t, J = 7.1 Hz, 3H). **HRMS** $C_{28}H_{33}ClN_2O_2$; calc. for $(M+H^+)$: 465.2231, found: 465.2.

7e2:
^{1}H NMR (600 MHz, $CDCl_3$) δ (ppm) 8.32 (d, J = 5.2 Hz, 1H), 7.11 - 7.13 (m, 3H), 7.07 (d, J = 5.3 Hz, 1H), 4.15 (q, J = 7.1 Hz, 2H), 3.38 - 3.42 (m, 1H), 3.18 - 3.28 (m, 2H), 3.12 - 3.15 (m, 1H), 3.01 - 3.05 (m, 1H), 2.81 - 2.87 (m, 1H), 2.75 - 2.79 (m, 1H), 2.38 - 2.42 (m, 3H), 2.19 - 2.21 (m, 1H), 1.87 - 1-91 (m, 1H), 1.80 - 1.85 (m, 3H), 1.72 - 1.73 (m, 1H), 1.40 - 1.46 (m, 6H), 1.26 (t, J = 7.1 Hz, 3H). **HRMS** $C_{28}H_{33}ClN_2O_2$; calc. for $(M+H^+)$: 465.2231, found: 465.2.

4-[13-chloro-5-(4,4-difluorocyclohexyl)-4-azatricyclo[9.4.0.03,8]pentadeca-1(15),3,5,7,11,13-hexaen-2-ylidene]piperidine-1-carboxylic acid ethyl ester (7j1), 4-[13-chloro-7-(4,4-difluorocyclohexyl)-4-azatricyclo[9.4.0.03,8]pentadeca-1(15),3,5,7,11,13-hexaen-2-ylidene]piperidine-1-carboxylic acid ethyl ester (7j2)

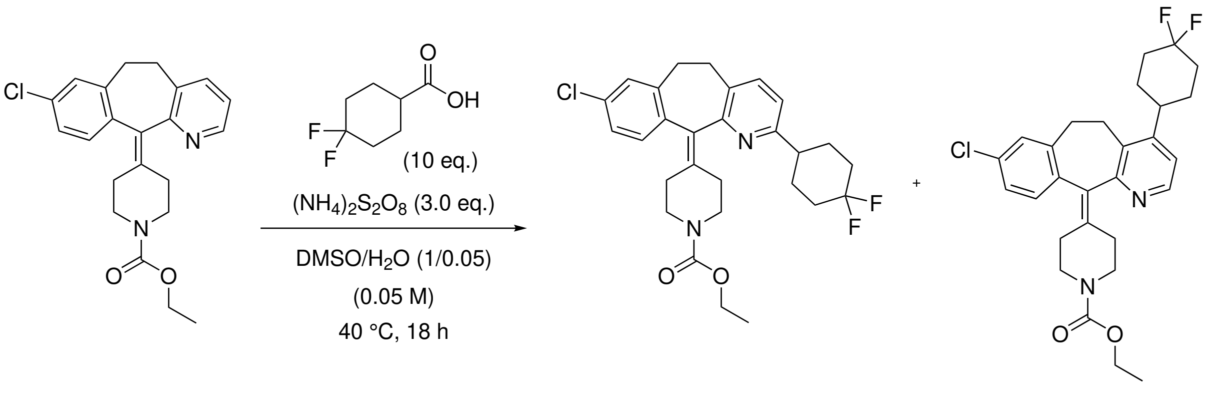

7 **7j1** (8%) **7j2** (6%)

Figure S15: Alkylation of Loratadine (**7**).

To a solution of 4-(13-chloro-4-azatricyclo[9.4.0.03,8]pentadeca-1(11),3(8),4,6,12,14-hexaen-2-ylidene)piperidine-1-carboxylic acid ethyl ester (**7**, 57.4 mg, 0.15 mmol, 1.00 eq.) in 3 mL degassed DMSO and 5 uL H_2O, 4,4-difluorocyclohexanecarboxylic acid (**j**, 246.2 mg, 1.50 mmol, 10.0 eq.) and ammonium persulfate (102.7 mg, 450 umol, 3.00 eq.) was added. The reaction mixture was degassed while bubbling nitrogen through it. The reaction mixture was stirred at 40 °C for 18 hr. The reaction mixture was quenched with $NaHCO_3$ solution and extracted with DCM. The combined organic layers were washed with water, and brine, dried over Na_2SO_4, filtered and concentrated to dryness. The crude material was purified by reversed-phase HPLC (YMC-Triart C18, 12 nm, 5 um, 100 x 30 mm) using a MeCN gradient (20-98%) in H_2O + 0.1% HCOOH. The solvent was removed from product containing fractions. Evaporation of solvents gave the title compounds 4-[13-chloro-5-(4,4-difluorocyclohexyl)-4-azatricyclo[9.4.0.03,8]pentadeca-1(15),3,5,7,11,13-hexaen-2-ylidene]piperidine-1-carboxylic acid ethyl ester (**7j1**, 7.8 mg, 8%) as an off-white powder and 4-[13-chloro-7-(4,4-difluorocyclohexyl)-4-azatricyclo[9.4.0.03,8]-pentadeca-1(15),3,5,7,11,13-hexaen-2-ylidene]piperidine-1-carboxylic acid ethyl ester (**7j2**, 6.0 mg, 6%) as an off-white powder.

7j1:
^{1}H NMR (600 MHz, CDCl$_3$) δ (ppm) 7.37 - 7.39 (m, 1H), 7.17 - 7.18 (m, 1H), 7.14 - 7.15 (m, 2H), 6.98 (d, J = 7.9 Hz, 1H), 4.15 (q, J = 7.1 Hz, 2H), 3.36 - 3.41 (m, 1H), 3.28 - 3.32 (m, 1H), 3.11 - 3.16 (m, 2H), 2.77 - 2.86 (m, 4H), 2.46 - 2.50 (m, 1H), 2.29 - 2.35 (m, 4H), 2.18 - 2.23 (m, 2H), 2.02 - 2.04 (m, 1H), 1.96 - 1.98 (m, 1H), 1.82 - 1.90 (m, 4H), 1.27 (t, J = 7.1 Hz, 3H). **HRMS** $C_{28}H_{31}ClF_2N_2O_2$; calc. for (M+H$^+$): 501.2042, found: 501.2.

7j2:
^{1}H NMR (600 MHz, CDCl$_3$) δ (ppm) 8.37 (d, J = 5.3 Hz, 1H), 7.12 - 7.14 (m, 3H), 7.09 (d, J = 5.3 Hz, 1H), 4.15 (q, J = 7.1 Hz, 2H), 3.41 - 3.45 (m, 1H), 3.28 - 3.33 (m, 1H), 3.14 - 3.25 (m, 2H), 2.98 - 3.02 (m, 1H), 2.80 - 2.90 (m, 3H), 2.38 - 2.44 (m, 3H), 2.24 - 2.31 (m, 2H), 2.16 - 2.19 (m, 1H), 1.78 - 1.93 (m, 6H), 1.26 (t, J = 7.1 Hz, 3H). **HRMS** $C_{28}H_{31}ClF_2N_2O_2$; calc. for (M+H$^+$): 501.2042, found: 501.2.

4-(13-chloro-5-tetrahydropyran-2-yl-4-azatricyclo[9.4.0.03,8]pentadeca-1(15),3,5,7,11,13-hexaen-2-ylidene)piperidine-1-carboxylic acid ethyl ester (7s)

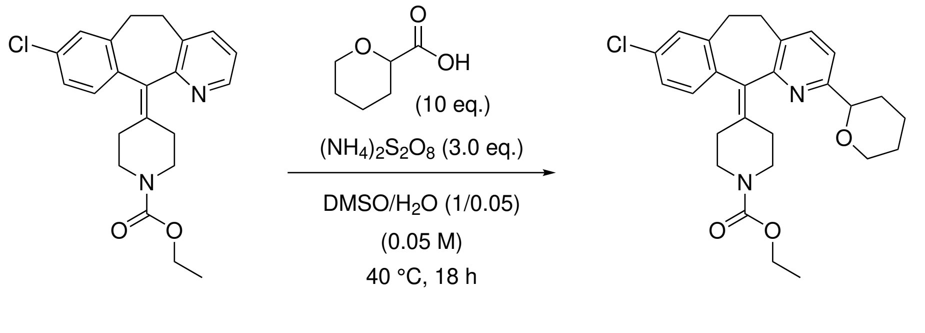

7 **7s** (10%)

Figure S16: Alkylation of Loratadine (**7**).

To a solution of 4-(13-chloro-4-azatricyclo[9.4.0.03,8]pentadeca-1(11),3(8),4,6,12,14-hexaen-2-ylidene)piperidine-1-carboxylic acid ethyl ester (**7**, 57.4 mg, 0.15 mmol, 1.00 eq.) in 3 mL degassed DMSO and 5 uL H_2O, tetrahydropyran-2-carboxylic acid (**s**, 195.2 mg, 1.50 mmol, 10.0 eq.) and ammonium persulfate (102.7 mg, 450 umol, 3.00 eq.) was added. The reaction mixture was degassed while bubbling nitrogen through it. The reaction mixture was stirred at 40 °C for 18 hr. The reaction mixture was quenched with $NaHCO_3$ solution and extracted with DCM. The combined organic layers were washed with water, and brine, dried over Na_2SO_4, filtered and concentrated to dryness. The crude material was purified by reversed-phase HPLC (Gemini NX, 12 nm, 5 um, 100 x 30 mm) using a MeCN gradient (30-65-90%) in H_2O + 0.1% HCOOH. The solvent was removed from product containing fractions. Evaporation of solvents gave the title compound 4-(13-chloro-5-tetrahydropyran-2-yl-4-azatricyclo[9.4.0.03,8]pentadeca-1(15),3,5,7,11,13-hexaen-2-ylidene)piperidine-1-carboxylic acid ethyl ester (**7s**, 7.1 mg, 10%) as an off-white powder.

^{1}H NMR (600 MHz, CDCl$_3$) δ (ppm) 7.45 (dd, J = 11.9, 7.9 Hz, 1H), 7.28 - 7.31 (m, 1H), 7.13 - 7.18 (m, 3H), 4.44 (dt, J = 11.2, 2.4 Hz, 1H), 4.12 - 4.17 (m, 3H), 3.59 - 3.66 (m, 1H), 3.29 - 3.39 (m, 2H), 2.98 - 3.12 (m, 2H), 2.78 - 2.87 (m, 2H), 2.31 - 2.46 (m, 4H), 1.88 - 1.95 (m, 1H), 1.65 - 1.73 (m, 3H), 1.57 - 1.60 (m, 1H), 1.40 - 1.47 (m, 1H), 1.26 (td, J = 7.1, 3.3 Hz, 4H). **HRMS** $C_{27}H_{31}ClN_2O_3$; calc. for (M+H$^+$): 467.2023, found: 467.2.

2-cyclopropyl-7-methyl-14-tetrahydropyran-2-yl-2,4,9,15-tetrazatricyclo[9.4.0.03,8]pentadeca-1(15)-,3(8),4,6,11,13-hexaen-10-one (8s)

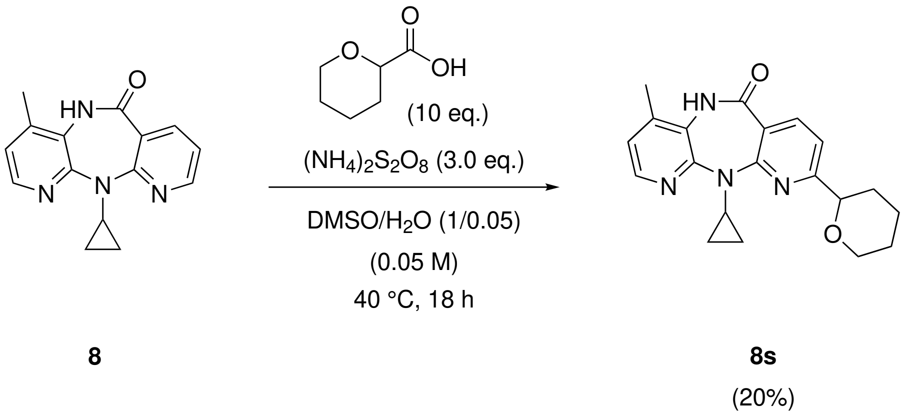

Figure S17: Alkylation of Nevirapine (**8**).

To a solution of 2-cyclopropyl-7-methyl-2,4,9,15-tetrazatricyclo[9.4.0.03,8]pentadeca-1(15),3,5,7,11,13-hexaen-10-one (**8**, 40.0 mg, 0.15 mmol, 1.00 eq.) in 3 mL degassed DMSO and 5 uL H_2O, tetrahydropyran-2-carboxylic acid (**s**, 195.2 mg, 1.50 mmol, 10.0 eq.) and ammonium persulfate (102.7 mg, 450 umol, 3.00 eq.) was added. The reaction mixture was degassed while bubbling nitrogen through it. The reaction mixture was stirred at 40 °C for 18 hr. The reaction mixture was quenched with $NaHCO_3$ solution and extracted with DCM. The combined organic layers were washed with water, and brine, dried over Na_2SO_4, filtered and concentrated to dryness. The crude material was purified by reversed-phase HPLC (Gemini NX, 12 nm, 5 um, 100 x 30 mm) using a MeCN gradient (20-45-65%) in H_2O + 0.1% HCOOH. The solvent was removed from product containing fractions. Evaporation of solvents gave the title compound 2-cyclopropyl-7-methyl-14-tetrahydropyran-2-yl-2,4,9,15-tetraza-tricyclo[9.4.0.03,8]pentadeca-1(15),3(8),4,6,11,13-hexaen-10-one (**8s**, 10.6 mg, 20%) as an off-white powder.

^{1}H NMR (600 MHz, $CDCl_3$) δ (ppm) 8.16 (d, J = 4.9 Hz, 1H), 8.12 (d, J = 7.9 Hz, 1H), 7.38 (s, 1H), 7.25 (m, 1H), 6.91 (dd, J = 4.9, 0.7 Hz, 1H), 4.39 - 4.46 (m, 1H), 4.14 - 4.16 (m, 1H), 3.71 - 3.74 (m, 1H), 3.61 - 3.66 (m, 1H), 2.35 (s, 3H), 2.16 - 2.23 (m, 1H), 1.93 - 1.96 (m, 1H), 1.58 - 1.73 (m, 4H), 1.43 - 1.50 (m, 1H), 0.90 - 0.96 (m, 2H), 0.41 - 0.48 (m, 2H). **HRMS** $C_{20}H_{22}N_4O_2$; calc. for $(M+H^+)$: 351.1743, found: 351.18.

4-[13-chloro-5-(4,4-difluorocyclohexyl)-4-azatricyclo[9.4.0.03,8]pentadeca-1(15),3,5,7,11,13-hexaen-2-ylidene]piperidine-1-carboxylic acid ethyl ester (7q1), 4-[5-(1-tert-butoxycarbonylpyrrolidin-3-yl)-13-chloro-4-azatricyclo[9.4.0.03,8]pentadeca-1(15),3,5,7,11,13-hexaen-2-ylidene]piperidine-1-carboxylic acid ethyl ester (7q2)

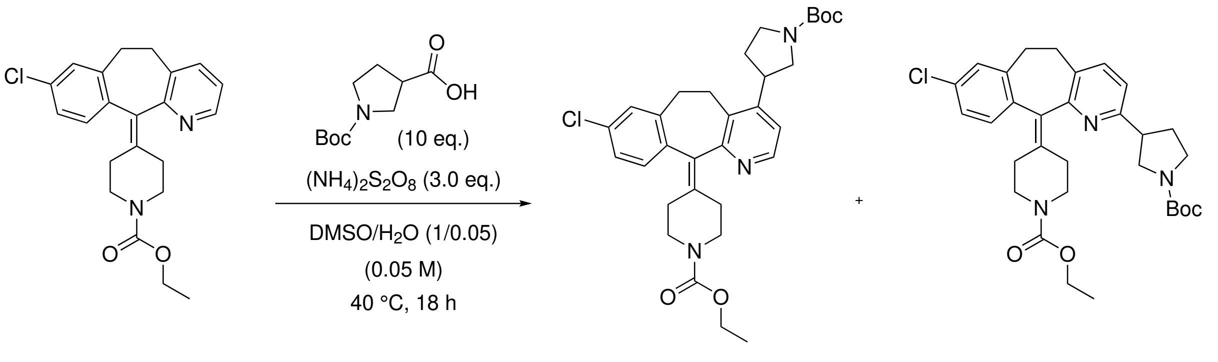

Figure S18: Alkylation of Loratadine (**7**).

To a solution of 4-(13-chloro-4-azatricyclo[9.4.0.03,8]pentadeca-1(11),3(8),4,6,12,14-hexaen-2-ylidene)piperidine-1-carboxylic acid ethyl ester (**7**, 57.4 mg, 0.15 mmol, 1.00 eq.) in 3 mL degassed DMSO and 5 uL H_2O, 1-tert-butoxycarbonylpyrrolidine-3-carboxylic acid (**q**, 322.9 mg, 1.50 mmol, 10.0 eq.) and ammonium persulfate (102.7 mg, 450 umol, 3.00 eq.) were added. The reaction mixture was degassed while bubbling nitrogen through it. The reaction mixture was stirred at 40 °C for 18 hr. The reaction mixture was quenched with $NaHCO_3$ solution and extracted with DCM. The combined organic layers were washed with water, and brine, dried over Na_2SO_4, filtered and concentrated to dryness. The crude material was purified by reversed-phase HPLC (Gemini NX, 12 nm, 5 µm, 100 x 30 mm) using a MeCN gradient (50-70-85-100%) in H_2O + 0.1% TEA. The solvent was removed from product containing fractions. Evaporation of solvents gave the title compounds 4-[5-(1-tert-butoxycarbonylpyrrolidin-3-yl)-13-chloro-4-azatricyclo[9.4.0.03,8]pentadeca-1(15),3,5,7,11,13-hexaen-2-ylidene]piperidine-1-carboxylic acid ethyl ester (**7j1**, 3.73 mg, 4%) as an off-white powder and 4-[5-(1-tert-butoxycarbonylpyrrolidin-3-yl)-13-chloro-4-azatricyclo[9.4.0.03,8]pentadeca-1(15),3,5,7,11,13-hexaen-2-ylidene]-piperidine-1-carboxylic acid ethyl ester (**7q2**, 3.69 mg, 4%) as an off-white powder.

7q1:
^{1}H NMR (600 MHz, $CDCl_3$) δ (ppm) 8.37 - 8.38 (m, 1H), 7.13 - 7.14 (m, 3H), 7.05 - 7.06 (m, 1H), 4.15 (d, J = 7.1 Hz, 2H), 3.77 - 3.81 (m, 2H), 3.56 - 3.63 (m, 2H), 3.41 - 3.46 (m, 2H), 3.25 - 3.34 (m, 2H), 3.14 - 3.20 (m, 2H), 3.00 - 3.05 (m, 1H), 2.82 - 2.86 (m, 1H), 2.41 - 2.43 (m, 3H), 2.18 - 2.26 (m, 2H), 1.50 (s, 9H), 1.26 (t, J = 7.1 Hz, 3H). **HRMS** $C_{28}H_{31}ClF_2N_2O_2$; calc. for (M+H$^+$): 501.2042, found: 501.2.

7q2:
^{1}H NMR (600 MHz, $CDCl_3$) δ (ppm) 7.33 - 7.35 (m, 1H), 7.17 - 7.18 (m, 1H), 7.12 - 7.15 (m, 2H), 6.97 - 6.99 (m, 1H), 4.15 (d, J = 7.1 Hz, 2H), 3.73 - 3.81 (m, 3H), 3.55 - 3.64 (m, 1H), 3.43 - 3.49 (m, 2H), 3.36 - 3.41 (m, 2H), 3.28 - 3.32 (m, 1H), 3.14 - 3.26 (m, 2H), 2.76 - 2.86 (m, 2H), 2.49 - 2.53 (m, 1H), 2.34 - 2.38 (m, 1H), 2.28 - 2.30 (m, 2H), 2.19 - 2.23 (m, 1H), 2.05 - 2.15 (m, 1H), 1.49 (s, 9H), 1.27 (t, J = 7.1 Hz, 3H). **HRMS** $C_{28}H_{31}ClF_2N_2O_2$; calc. for (M+H$^+$): 501.2042, found: 501.2.

N-[(6-tert-butyl-3-pyridyl)methyl]-N-methyl-1-naphthamide (**28h**)

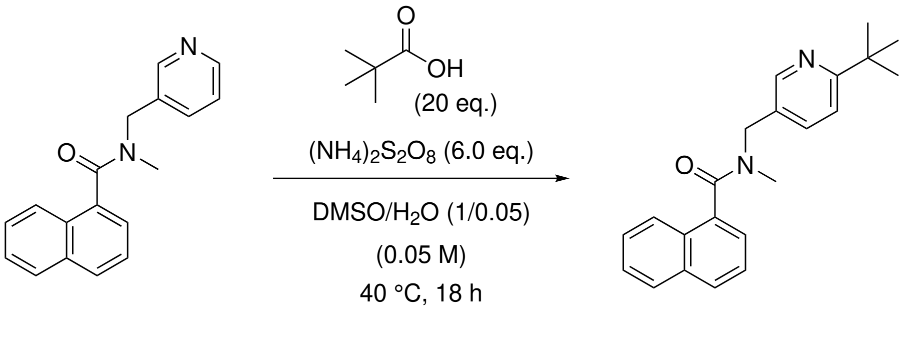

28 **28h** (28%)

Figure S19: Alkylation of Fragment **28**.

To a solution of N-methyl-N-(3-pyridylmethyl)-1-naphthamide (**28**, 41.5 mg, 0.15 mmol, 1.00 eq.) in 3 mL degassed DMSO and 5 uL H_2O, pivalic acid (**h**, 306.4 mg, 348.2 uL, 3.0 mmol, 20.0 eq.) and ammonium persulfate (205.4 mg, 0.9 mmol, 6.0 eq.) were added. The reaction mixture was degassed while bubbling nitrogen through it. The reaction mixture was stirred at 40 °C for 18 hr. The reaction mixture was quenched with $NaHCO_3$ solution and extracted with DCM. The combined organic layers were washed with water, and brine, dried over Na_2SO_4, filtered and concentrated to dryness. The crude material was purified by reversed-phase HPLC (Gemini NX, 12 nm, 5 um, 100 x 30 mm) using a MeCN gradient (30-50-65-100%) in H_2O + 0.1% TEA. The solvent was removed from product containing fractions. Evaporation of solvents gave the title compound N-[(6-tert-butyl-3-pyridyl)methyl]-N-methyl-1-naphthamide (**28h**, 14.2 mg, 28%) as an off-white powder.

^{1}H NMR (600 MHz, $CDCl_3$) δ (ppm) 8.62 - 8.63 (m, 1H), 7.88 - 7.90 (m, 4H), 7.86 - 7.91 (m, 2H), 7.42 - 7.47 (m, 3H), 3.17 (s, 2H), 2.75 (s, 3H), 1.41 (s, 10H). **HRMS** $C_{22}H_{24}N_2O$; calc. for $(M+H^+)$: 333.1889, found: 333.19.

3-[4-(4-bromo-3-methyl-phenyl)-5-(cyclohexylthio)-1,2,4-triazol-3-yl]-2-phenoxy-pyridine (**38e**)

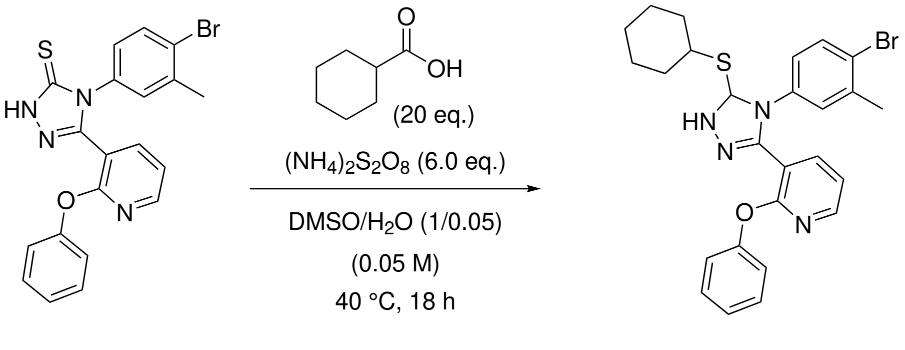

38

38e
(36%)

Figure S20: Alkylation of Fragment **38**.

To a solution of 4-(4-bromo-3-methyl-phenyl)-3-(2-phenoxy-3-pyridyl)-1H-1,2,4-triazole-5-thione (**38**, 41.5 mg, 0.15 mmol, 1.00 eq.) in 3 mL degassed DMSO and 5 uL H_2O, cyclohexanecarboxylic acid (**e**, 384.5 mg, 373.31 uL, 3.0 mmol, 20.0 eq.) and ammonium persulfate (205.4 mg, 0.9 mmol, 6.0 eq.) were added. The reaction mixture was degassed while bubbling nitrogen through it. The reaction mixture was stirred at 40 °C for 18 hr. The reaction mixture was quenched with $NaHCO_3$ solution and extracted with DCM. The combined organic layers were washed with water, and brine, dried over Na_2SO_4, filtered and concentrated to dryness. The crude material was purified by reversed-phase HPLC (YMC-Triart C18, 12 nm, 5 um, 100 x 30 mm) using a MeCN gradient (20-98%) in H_2O + 0.1% HCOOH. The solvent was removed from product containing fractions. Evaporation of solvents gave the title compound 3-[4-(4-bromo-3-methyl-phenyl)-5-(cyclohexylthio)-1,2,4-triazol-3-yl]-2-phenoxy-pyridine (**38e**, 28.5 mg, 36%) as an off-white powder.

^{1}H NMR (600 MHz, $CDCl_3$) δ (ppm) 8.18 (dd, J = 4.9, 2.0 Hz, 1H), 8.11 - 8.15 (m, 1H), 7.48 (d, J = 8.4 Hz, 1H), 7.28 - 7.29 (m, 1H), 7.26 - 7.27 (m, 1H), 7.14 - 7.16 (m, 1H), 7.11 - 7.13 (m, 1H), 7.02 (d, J = 2.6 Hz, 1H), 6.77 (d, J = 8.4 Hz, 1H), 6.47 (d, J = 7.7 Hz, 2H), 3.86 - 3.91 (m, 1H), 2.19 (s, 3H), 2.16 - 2.18 (m, 1H), 1.73 - 1.77 (m, 2H), 1.61 - 1.64 (m, 2H), 1.49 - 1.52 (m, 2H), 1.4.1 - 1.49 (m, 3H), 1.25 - 1. 30 (s, 1H). **HRMS** $C_{22}H_{24}N_2O$; calc. for ($M+H^+$): 523.1089, found: 523.09.

4-(6-tert-butyl-3-pyridyl)-3-(2,3-dichlorophenyl)-1,2,4-oxadiazol-5-one (40h)

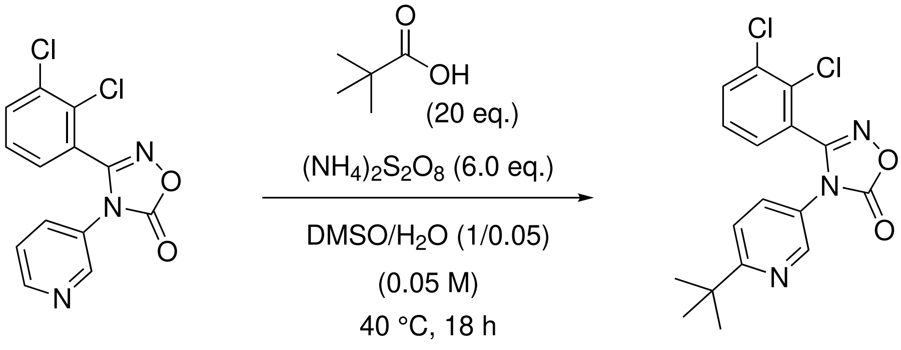

40

40h
(52%)

Figure S21: Alkylation of Fragment **40**.

To a solution of 3-(2,3-dichlorophenyl)-4-(3-pyridyl)-1,2,4-oxadiazol-5-one (**40**, 46.2 mg, 0.15 mmol, 1.00 eq.) in 3 mL degassed DMSO and 5 uL H_2O, pivalic acid (**h**, (306.4 mg, 348.2 uL, 3.0 mmol, 20.0 eq.) and ammonium persulfate (205.4 mg, 0.9 mmol, 6.0 eq.) were added. The reaction mixture was degassed while bubbling nitrogen through it. The reaction mixture was stirred at 40 °C for 18 hr. The reaction mixture was quenched with $NaHCO_3$ solution and extracted with DCM. The combined organic layers were washed with water, and brine, dried over Na_2SO_4, filtered and concentrated to dryness. The crude material was purified by reversed-phase HPLC (YMC-Triart C18, 12 nm, 5 um, 100 x 30 mm) using a MeCN gradient (20-98%) in H_2O + 0.1% HCOOH. The solvent was removed from product containing fractions. Evaporation of solvents gave the title compound 4-(6-tert-butyl-3-pyridyl)-3-(2,3-dichlorophenyl)-1,2,4-oxadiazol-5-one (**40h**, 28.6 mg, 52%) as an off-white powder.

^{1}H NMR (600 MHz, $CDCl_3$) δ (ppm) 8.24 (dd, J = 2.6, 0.7 Hz, 1H), 7.60 (dd, J = 8.7, 2.6 Hz, 1H), 7.49 (d, J = 1.6 Hz, 1H), 7.38 - 7.42 (m, 2H), 1.33 (s, 9H). **HRMS** $C_{17}H_{15}Cl_2N_3O_2$; calc. for (M+H$^+$): 364.0541, found: 364.06.

N-[(6-tert-butyl-3-pyridyl)methyl]-4-chloro-N-(4-chlorobenzyl)benzenesulfonamide (33h)

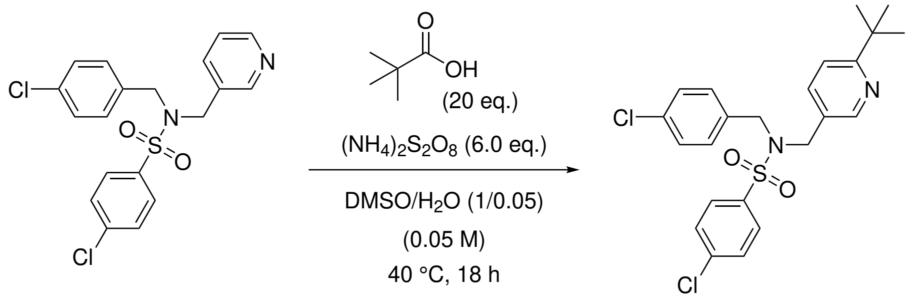

33

33h
(44%)

Figure S22: Alkylation of Fragment **33**.

To a solution of 4-chloro-N-(4-chlorobenzyl)-N-(3-pyridylmethyl)benzenesulfonamide (**33**, 61.1 mg, 0.15 mmol, 1.00 eq.) in 3 mL degassed DMSO and 5 uL H_2O, pivalic acid (**h**, (306.4 mg, 348.2 uL, 3.0 mmol, 20.0 eq.) and ammonium persulfate (205.4 mg, 0.9 mmol, 6.0 eq.) were added. The reaction mixture was degassed while bubbling nitrogen through it. The reaction mixture was stirred at 40 °C for 18 hr. The reaction mixture was quenched with $NaHCO_3$ solution and extracted with DCM. The combined organic layers were washed with water, and brine, dried over Na_2SO_4, filtered and concentrated to dryness. The crude material was purified by reversed-phase HPLC (Gemini NX, 12 nm, 5 um, 100 x 30 mm) using a MeCN gradient (20-98-100%) in H_2O + 0.1% HCOOH. The solvent was removed from product containing fractions. Evaporation of solvents gave the title compound N-[(6-tert-butyl-3-pyridyl)methyl]-4-chloro-N-(4-chlorobenzyl)benzenesulfonamide (**40h**, 30.8 mg, 44%) as an off-white powder.

^{1}H NMR (600 MHz, $CDCl_3$) δ (ppm) 8.17 - 8.19 (m, 1H), 7.77 (d, J = 8.6 Hz, 2H), 7.51 (d, J = 8.1 Hz, 2H), 7.36 - 7.38 (m, 1H), 7.16 (d, J = 8.4 Hz, 3H), 7.02 (d, J = 7.9 Hz, 2H), 4.29 (s, 2H), 4.28 (s, 2H), 1.32 (s, 9H). **HRMS** $C_{23}H_{24}Cl_2N_2O_2S$; calc. for $(M{+}H^+)$: 463.0936, found: 463.10.

N-(4-chlorophenyl)-2-cyclobutyl-6-(phenylthio)-4-(trifluoromethyl)nicotinamide (37b1), N-(4-chlorophenyl)-5-cyclobutyl-6-(phenylthio)-4-(trifluoromethyl)nicotinamide (37b2)

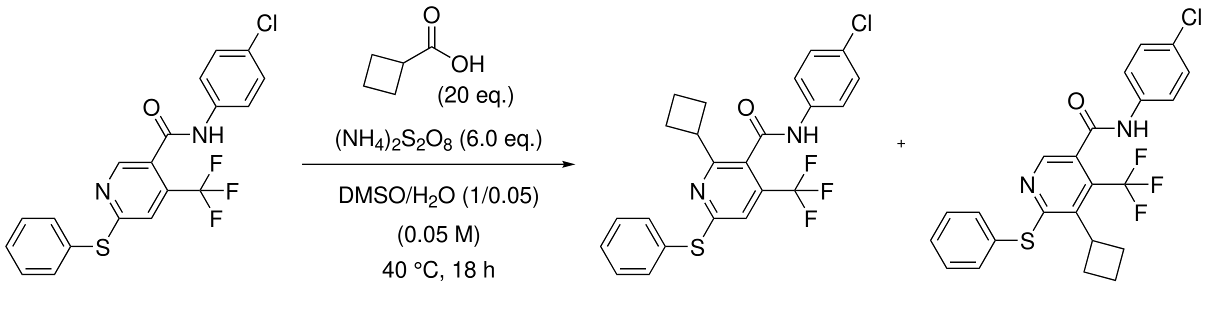

37 **37b1** (10%) **37b2** (7%)

Figure S23: Alkylation of fragment **37**.

To a solution of N-(4-chlorophenyl)-6-(phenylthio)-4-(trifluoromethyl)nicotinamide (**37**, 61.3 mg, 0.15 mmol, 1.00 eq.) in 3 mL degassed DMSO and 5 uL H_2O, cyclobutanecarboxylic acid (**b**, 300.4 mg, 3.0 mmol, 10.0 eq.) and ammonium persulfate (205.4 mg, 900 umol, 3.00 eq.) were added. The reaction mixture was degassed while bubbling nitrogen through it. The reaction mixture was stirred at 40 °C for 18 hr. The reaction mixture was quenched with $NaHCO_3$ solution and extracted with DCM. The combined organic layers were washed with water, and brine, dried over Na_2SO_4, filtered and concentrated to dryness. The crude material was purified by reversed-phase HPLC (YMC-Triart C18, 12 nm, 5 µm, 100 x 30 mm) using a MeCN gradient (50-20-98%) in H_2O + 0.1% TEA. The solvent was removed from product containing fractions. Evaporation of solvents gave the title compounds N-(4-chlorophenyl)-2-cyclobutyl-6-(phenylthio)-4-(trifluoromethyl)nicotinamide (**37b1**, 11.5 mg, 10%) and N-(4-chlorophenyl)-5-cyclobutyl-6-(phenylthio)-4-(trifluoromethyl)nicotinamide (**37b2**, 11.5 mg, 7%) as a mixture (60:40) in an off-white powder.

37b1 & **37b2**:
^{1}H NMR (600 MHz, $CDCl_3$) δ (ppm) 8.31 - 8.35 (m, 1H), 8.11 - 8.13 (m, 1H), 7.64 - 7.67 (m, 2H), 7.51 - 7.54 (m, 2H), 7.50 - 7.55 (m, 2H), 7.50 - 7.52 (m, 3H), 7.45 (d, J = 3.8 Hz, 1H), 7.35 - 7.39 (m, 3H), 7.33 (s, 1H), 7.21 - 7.26 (m, 1H), 7.01 (s, 1H), 4.03 - 4.17 (m, 1H), 3.74 - 3.86 (m, 1H), 2.77 - 2.87 (m, 1H), 2.54 - 2.65 (m, 1H), 2.31 - 2.46 (m, 3H), 2.14 - 2.25 (m, 2H), 2.08 - 2.16 (m, 1H), 1.98 - 2.04 (m, 1H), 1.92 - 1.97 (m, 1H), 1.74 - 1.84 (m, 1H). **HRMS** $C_{23}H_{18}ClF_3N_2OS$; calc. for $(M+H^+)$: 463.0780, found: 463.08.

N-(4-chlorophenyl)-6-cyclobutyl-2-phenoxy-4-(trifluoromethyl)nicotinamide (29b)

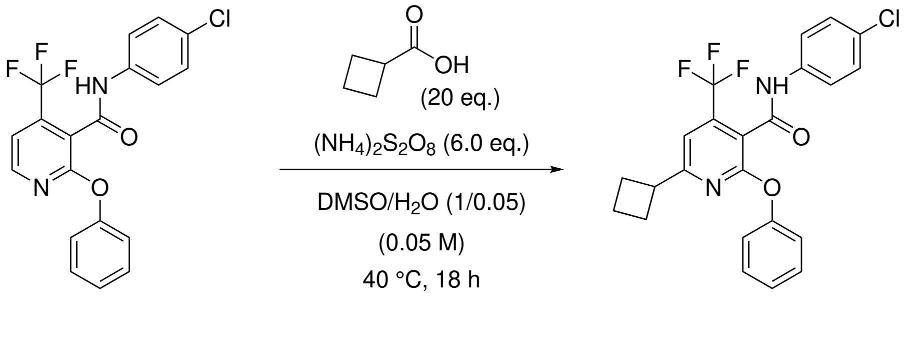

Figure S24: Alkylation of Fragment **29**.

To a solution of N-(4-chlorophenyl)-2-phenoxy-4-(trifluoromethyl)nicotinamide (**29**, 58.9 mg, 0.15 mmol, 1.00 eq.) in 3 mL degassed DMSO and 5 uL H_2O, cyclobutanecarboxylic acid (**b**, (300.4 mg, 3.0 mmol, 20.0 eq.) and ammonium persulfate (205.4 mg, 0.9 mmol, 6.0 eq.) were added. The reaction mixture was degassed while bubbling nitrogen through it. The reaction mixture was stirred at 40 °C for 18 hr. The reaction mixture was quenched with $NaHCO_3$ solution and extracted with DCM. The combined organic layers were washed with water, and brine, dried over Na_2SO_4, filtered and concentrated to dryness. The crude material was purified by reversed-phase HPLC (YMC-Triart C18, 12 nm, 5 um, 100 x 30 mm) using a MeCN gradient (20-98%) in H_2O + 0.1% HCOOH. The solvent was removed from product containing fractions. Evaporation of solvents gave the title compound N-(4-chlorophenyl)-6-cyclobutyl-2-phenoxy-4-(trifluoromethyl)nicotinamide (**29b**, 15.0 mg, 22%) as an off-white powder.

^{1}H NMR (600 MHz, $CDCl_3$) δ (ppm) 7.55 - 7.58 (m, 2H), 7.33 - 7.36 (m, 2H), 7.18 - 7.24 (m, 3H), 7.13 (s, 1H), 3.56 (t, J = 8.4 Hz, 1H), 2.22 - 2.28 (m, 2H), 2.11 - 2.16 (m, 2H), 1.92 - 2.00 (m, 1H), 1.77 - 1.82 (m, 1H).
HRMS $C_{23}H_{18}ClF_3N_2O_2$; calc. for (M+H$^+$): 447.1009, found: 447.10.

6-cyclobutyl-N-[keto-methyl-(p-tolyl)persulfuranylidene]-2-phenoxy-nicotinamide (34b1), 4-cyclobutyl-N-[keto-methyl-(p-tolyl)persulfuranylidene]-2-phenoxy-nicotinamide (34b2)

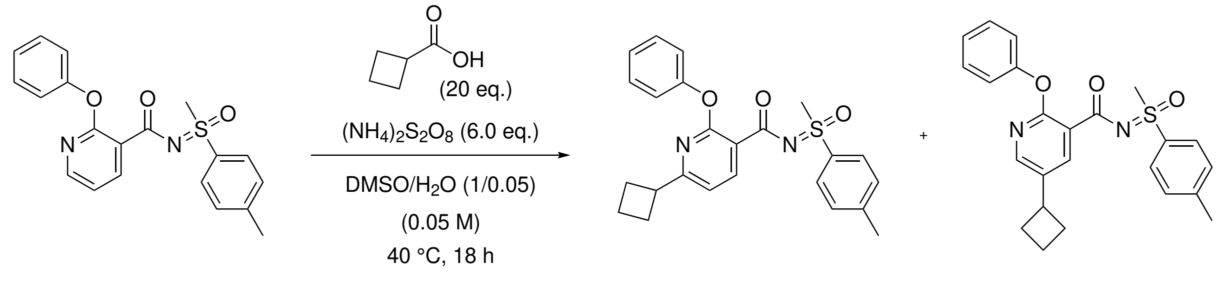

34 **34b1** (15%) **34b2** (4%)

Figure S25: Alkylation of fragment **34**.

To a solution of N-[keto-methyl-(p-tolyl)persulfuranylidene]-2-phenoxy-nicotinamide (**34**, 55.0 mg, 0.15 mmol, 1.00 eq.) in 3 mL degassed DMSO and 5 uL H_2O, cyclobutanecarboxylic acid (**b**, 300.4 mg, 3.0 mmol, 10.0 eq.) and ammonium persulfate (205.4 mg, 900 umol, 3.00 eq.) were added. The reaction mixture was degassed while bubbling nitrogen through it. The reaction mixture was stirred at 40 °C for 18 hr. The reaction mixture was quenched with $NaHCO_3$ solution and extracted with DCM. The combined organic layers were washed with water, and brine, dried over Na_2SO_4, filtered and concentrated to dryness. The crude material was purified by reversed-phase HPLC (Gemini NX, 12 nm, 5 µm, 100 x 30 mm) using a MeCN gradient (20-98-100%) in H_2O + 0.1% HCOOH. The solvent was removed from product containing fractions. Evaporation of solvents gave the title compounds 6-cyclobutyl-N-[keto-methyl-(p-tolyl)persulfuranylidene]-2-phenoxy-nicotinamide (**34b1**, 9.7 mg, 15%) as an off-white powder and 4-cyclobutyl-N-[keto-methyl-(p-tolyl)persulfuranylidene]-2-phenoxy-nicotinamide (**34b2**, 2.7 mg, 4%) as an off-white powder.

34b1:
^{1}H NMR (600 MHz, $CDCl_3$) δ (ppm) 8.25 (d, J = 7.7 Hz, 1H), 7.89 (d, J = 8.4 Hz, 1H), 7.32 - 7.37 (m, 4H), 7.13 - 7.15 (m, 3H), 6.93 (d, J = 7.7 Hz, 1H), 3.49 - 3.55 (m, 1H), 3.36 (s, 3H), 2.44 (s, 3H), 2.16 - 2.23 (m, 4H), 1.89 - 1.97 (m, 1H), 1.76 - 1.81 (m, 1H). **HRMS** $C_{24}H_{24}N_2O_3S$; calc. for (M+H$^+$): 421.1508, found: 421.15.

34b2:
^{1}H NMR (600 MHz, $CDCl_3$) δ (ppm) 8.09 (d, J = 5.3 Hz, 1H), 7.91 (d, J = 8.4 Hz, 2H), 7.40 - 7.44 (m, 2H), 7.28 - 7.29 (m, 2H), 7.17 - 7.21 (m, 3H), 7.02 (dd, J = 5.3, 0.7 Hz, 1H), 3.86 - 3.92 (m, 1H), 3.35 (s, 3H), 2.42 (s, 3H), 2.21 - 2.24 (m, 2H), 2.02 - 2.07 (m, 1H), 1.83 - 1.87 (m, 1H). **HRMS** $C_{24}H_{24}N_2O_3S$; calc. for (M+H$^+$): 421.1508, found: 421.15.

2-[(5-methyl-2-pyridyl)methylthio]-4-tetrahydropyran-4-yl-3H-benzo[f]benzimidazole (39u)

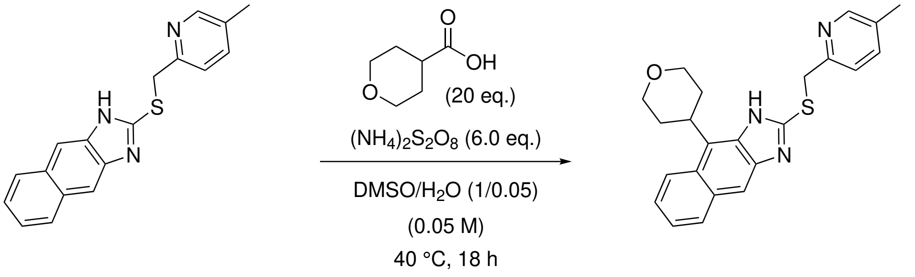

Figure S26: Alkylation of Fragment **39**.

To a solution of 2-[(5-methyl-2-pyridyl)methylthio]-1H-benzo[f]benzimidazole (**39**, 45.8 mg, 0.15 mmol, 1.00 eq.) in 3 mL degassed DMSO and 5 uL H_2O, tetrahydropyran-4-carboxylic acid (**u**, (390.4 mg, 3.0 mmol, 20.0 eq.) and ammonium persulfate (205.4 mg, 0.9 mmol, 6.0 eq.) were added. The reaction mixture was degassed while bubbling nitrogen through it. The reaction mixture was stirred at 40 °C for 18 hr. The reaction mixture was quenched with $NaHCO_3$ solution and extracted with DCM. The combined organic layers were washed with water, and brine, dried over Na_2SO_4, filtered and concentrated to dryness. The crude material was purified by reversed-phase HPLC (Gemini NX, 12 nm, 5 um, 100 x 30 mm) using a MeCN gradient (20-50-55-100%) in H_2O + 0.1% HCOOH. The solvent was removed from product containing fractions. Evaporation of solvents gave the title compound 2-[(5-methyl-2-pyridyl)methylthio]-4-tetrahydropyran-4-yl-3H-benzo[f]benzimidazole (**39u**, 2.0 mg, 3%) as an off-white powder.

^{1}H NMR (600 MHz, $CDCl_3$) δ (ppm) 8.83 (s, 1H), 8.30 - 8.31 (m, 1H), 7.99 - 8.03 (m, 2H), 7.61 - 7.63 (m, 1H), 7.46 - 7.48 (m, 1H), 7.39 - 7.41 (m, 1H), 7.30 - 7.31 (m, 1H), 4.40 (s, 2H), 4.32 - 4.31 (m, 2H), 4.04 - 4.08 (m, 1H), 3.81 - 3.85 (m, 2H), 2.68 - 2.74 (m, 2H), 2.41 - 2.43 (m, 3H), 1.89 - 1.91 (m, 2H). **^{13}C NMR (151 MHz, $CDCl_3$)** δ (ppm) 138.8, 130.9, 129.5, 127.4, 124.1, 123.1, 123.0, 114.1, 60.0, 37.4, 31.0, 18.5. **HRMS** $C_{23}H_{23}N_3OS$; calc. for $(M+H^+)$: 390.1562, found: 390.16.

2-[6-(4-chlorophenoxy)-2-(tetrahydrofuryl)-4-(trifluoromethyl)-3-pyridyl]-5-(4-chlorophenyl)oxazole (35m)

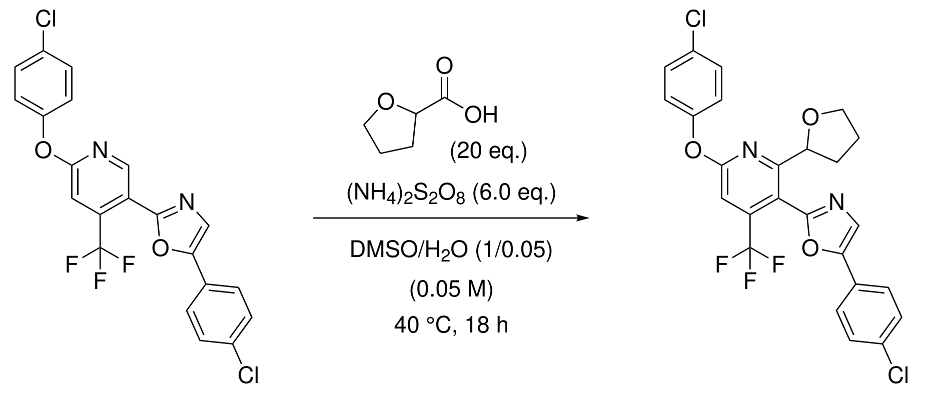

35

35m
(3%)

Figure S27: Alkylation of Fragment **35**.

To a solution of 2-[6-(4-chlorophenoxy)-4-(trifluoromethyl)-3-pyridyl]-5-(4-chlorophenyl)oxazole (**35**, 67.7 mg, 0.15 mmol, 1.00 eq.) in 3 mL degassed DMSO and 5 uL H_2O, tetrahydrofuran-2-carboxylic acid (**m**, (348.5 mg, 3.0 mmol, 20.0 eq.) and ammonium persulfate (205.4 mg, 0.9 mmol, 6.0 eq.) were added. The reaction mixture was degassed while bubbling nitrogen through it. The reaction mixture was stirred at 40 °C for 18 hr. The reaction mixture was quenched with $NaHCO_3$ solution and extracted with DCM. The combined organic layers were washed with water, and brine, dried over Na_2SO_4, filtered and concentrated to dryness. The crude material was purified by reversed-phase HPLC (Gemini NX, 12 nm, 5 um, 100 x 30 mm) using a MeCN gradient (60-80-95-100%) in H_2O + 0.1% HCOOH. The solvent was removed from product containing fractions. Evaporation of solvents gave the title compound 2-[6-(4-chlorophenoxy)-2-(tetrahydrofuryl)-4-(trifluoromethyl)-3-pyridyl]-5–(4-chlorophenyl)oxazole (**35m**, 2.0 mg, 3%) as an off-white powder.

^{1}H NMR (600 MHz, $CDCl_3$) δ (ppm) 7.60 (d, J = 8.1 Hz, 2H), 7.50 (s, 1H), 7.40 - 7.43 (m, 4H), 7.24 (s, 1H), 7.16 (d, J = 8.4 Hz, 2H), 4.99 (dd, J = 7.6, 5.5 Hz, 1H), 3.76 (td, J = 7.6, 5.7 Hz, 1H), 3.68 (q, J = 7.3 Hz, 1H), 2.02 - 2.08 (m, 1H), 2.01 - 2.18 (m, 1H), 1.79 - 1.87 (m, 2H). **^{13}C NMR (151 MHz, $CDCl_3$)** δ (ppm) 163.9, 163.8, 155.3, 151.6, 151.4, 134.7, 130.7, 129.6, 129.3, 126.1, 125.6, 123.3, 123.0, 114.1, 107.6, 78.0, 69.7, 34.3, 32.3, 30.3, 29.7, 26.0 . **HRMS** $C_{25}H_{17}Cl_2F_3N_2O_3$; calc. for ($M+H^+$): 521.0568, found: 521.06.

Benzoic acid (4-cyclohexyl-8-quinolyl) ester (**41e**)

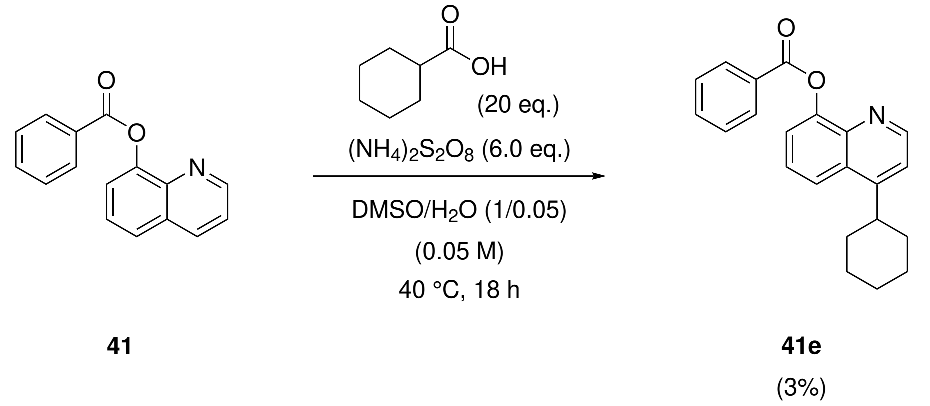

Figure S28: Alkylation of Fragment **41**.

To a solution of Benzoic acid 8-quinolyl ester (**41**, 67.7 mg, 0.15 mmol, 1.00 eq.) in 3 mL degassed DMSO and 5 uL H_2O, cyclohexanecarboxylic acid (**e**, (384.5 mg, 3.0 mmol, 20.0 eq.) and ammonium persulfate (205.4 mg, 0.9 mmol, 6.0 eq.) were added. The reaction mixture was degassed while bubbling nitrogen through it. The reaction mixture was stirred at 40 °C for 18 hr. The reaction mixture was quenched with $NaHCO_3$ solution and extracted with DCM. The combined organic layers were washed with water, and brine, dried over Na_2SO_4, filtered and concentrated to dryness. The crude material was purified by reversed-phase HPLC (Gemini NX, 12 nm, 5 um, 100 x 30 mm) using a MeCN gradient (60-80-95-100%) in H_2O + 0.1% HCOOH. The solvent was removed from product containing fractions. Evaporation of solvents gave the title compound Benzoic acid (4-cyclohexyl-8-quinolyl) ester (**41e**, 1.5 mg, 3%) as an off-white powder.

^{1}H NMR (600 MHz, $CDCl_3$) δ (ppm) 8.81 (d, J = 4.5 Hz, 1H), 8.36 (dd, J = 8.4, 1.3 Hz, 2H), 8.04 - 8.06 (m, 1H), 7.65 - 7.68 (m, 1H), 7.58 - 7.61 (m, 1H), 7.54 - 7.56 (m, 1H), 7.53 - 7.57 (m, 2H), 7.30 - 7.31 (m, 1H), 3.31 - 3.39 (m, 1H), 2.02 - 2.07 (m, 2H), 1.93 - 1.99 (m, 2H), 1.84 - 1.90 (m, 1H), 1.54 - 1.59 (m, 6H). **^{13}C NMR (151 MHz, $CDCl_3$)** δ (ppm) 165.6, 153.4, 150.6, 148.4, 141.6, 133.5, 130.6, 129.7, 128.5, 125.6, 121.4, 120.9, 118.1, 39.2, 33.7, 27.0, 26.4. **HRMS** $C_{22}H_{21}NO_2$; calc. for $(M+H^+)$: 332.1572, found: 332.16.

7-chloro-4-(4-chloro-3-methyl-phenoxy)-2-cyclohexyl-quinoline (26e)

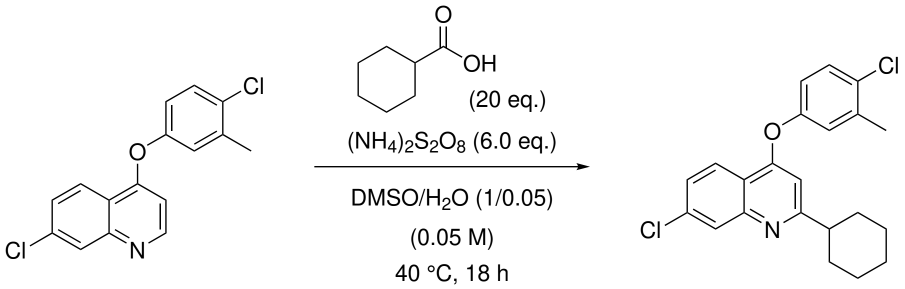

26 **26e** (22%)

Figure S29: Alkylation of Fragment **26**.

To a solution of 7-chloro-4-(4-chloro-3-methyl-phenoxy)quinoline (**26**, 45.6 mg, 0.15 mmol, 1.00 eq.) in 3 mL degassed DMSO and 5 uL H_2O, cyclohexanecarboxylic acid (**e**, 384.5 mg, 3.0 mmol, 20.0 eq.) and ammonium persulfate (205.4 mg, 0.9 mmol, 6.0 eq.) were added. The reaction mixture was degassed while bubbling nitrogen through it. The reaction mixture was stirred at 40 °C for 18 hr. The reaction mixture was quenched with $NaHCO_3$ solution and extracted with DCM. The combined organic layers were washed with water, and brine, dried over Na_2SO_4, filtered and concentrated to dryness. The crude material was purified by reversed-phase HPLC (Gemini NX, 12 nm, 5 um, 100 x 30 mm) using a MeCN gradient (60-80-95-100%) in H_2O + 0.1% HCOOH. The solvent was removed from product containing fractions. Evaporation of solvents gave the title compound 7-chloro-4-(4-chloro-3-methyl-phenoxy)-2-cyclohexyl-quinoline (**26e**, 12.9 mg, 22%) as an off-white powder.

^{1}H NMR (600 MHz, $CDCl_3$) δ (ppm) 8.18 (d, J = 8.9 Hz, 1H), 8.05 (d, J = 2.1 Hz, 1H), 7.45 (dd, J = 8.9, 2.1 Hz, 1 H), 7.44 (d, J = 8.6 Hz, 1 H), 7.07 - 7.08 (m, 1 H), 6.94 - 6.96 (m, 1 H), 6.46 (s, 1 H), 2.72 - 2.76 (m, 1 H), 2.43 (s, 3 H), 1.86 - 1.92 (m, 2 H), 1.83 - 1.85 (m, 2 H), 1.73 - 1.75 (m, 1 H), 1.46 - 1.50 (m, 2 H), 1.38 - 1.41 (m, 2 H), 1.25 - 1.31 (m, 1 H). **HRMS** $C_{22}H_{21}Cl_2NO$; calc. for $(M+H^+)$: 386.1000, found: 386.10.

Supplementary References

1. Fey, M. & Lenssen, J. E. *Fast Graph Representation Learning with PyTorch Geometric* in *ICLR Workshop on Representation Learning on Graphs and Manifolds* (2019).
2. Paszke, A. *et al.* Pytorch: An imperative style, high-performance deep learning library. *Advances in neural information processing systems* **32,** 8026–8037 (2019).
3. Kingma, D. P. & Ba, J. Adam: A method for stochastic optimization. *arXiv:1412.6980* (2014).
4. Rogers, D. & Hahn, M. Extended-connectivity fingerprints. *J. Chem. Inf. Model.* **50,** 742–754 (2010).
5. Nippa, D. F. *et al.* Enabling late-stage drug diversification by high-throughput experimentation with geometric deep learning (2022).
6. Atz, K., Isert, C., Böcker, M. N., Jiménez-Luna, J. & Schneider, G. Δ-Quantum machine-learning for medicinal chemistry. *Phys. Chem. Chem. Phys.* **24,** 10775–10783 (2022).
7. Isert, C., Atz, K., Jiménez-Luna, J. & Schneider, G. QMugs, quantum mechanical properties of drug-like molecules. *Sci. Data* **9,** 1–11 (2022).
8. Isert, C., Atz, K., Jiménez-Luna, J. & Schneider, G. *QMugs: Quantum Mechanical Properties of Drug-like Molecules* en. 2021.
9. Neeser, R., Isert, C., Stuyver, T., Schneider, G. & Coley, C. Qmugs 1.1: Quantum Mechanical Properties of Organic Compounds Commonly Encountered in Reactivity Datasets. *SSRN 4363768* (2023).
10. Proctor, R., Chuentragool, P., Colgan, A. & Phipps, R. Hydrogen Atom Transfer-Driven Enantioselective Minisci Reaction of Amides. *J. Am. Chem. Soc.* **143,** 4928–4934 (2021).
11. Reid, J., Proctor, R., Sigman, M. & Phipps, R. Predictive Multivariate Linear Regression Analysis Guides Successful Catalytic Enantioselective Minisci Reactions of Diazines. *J. Am. Chem. Soc.* **141,** 19178–19185 (2019).
12. Bieszczad, B., Perego, L. & Melchiorre, P. Photochemical C-H Hydroxyalkylation of Quinolines and Isoquinolines. *Angew. Chem. Int. Ed.* **58,** 16878–16883 (2019).
13. Chen, X. *et al.* Histidine-Specific Peptide Modification via Visible-Light-Promoted C-H Alkylation. *J. Am. Chem. Soc.* **141,** 18230–18237 (2019).
14. Fu, M.-C., Shang, R., Zhao, B., Wang, B. & Fu, Y. Photocatalytic decarboxylative alkylations mediated by triphenylphosphine and sodium iodide. *Science* **363,** 1429–1434 (2019).
15. Wang, Z., Ji, X., Zhao, J. & Huang, H. Visible-light-mediated photoredox decarbonylative Minisci-type alkylation with aldehydes under ambient air conditions. *Green Chem.* **21,** 5512–5516 (2019).
16. Dong, J. *et al.* Visible-light-mediated Minisci C-H alkylation of heteroarenes with unactivated alkyl halides using O2 as an oxidant. *Chem. Sci.* **10,** 976–982 (2019).
17. Li, G.-X., Hu, X., He, G. & Chen, G. Photoredox-Mediated Minisci-type Alkylation of N-Heteroarenes with Alkanes with High Methylene Selectivity. *ACS Catalysis* **8,** 11847–11853 (2018).
18. Proctor, R., Davis, H. & Phipps, R. Catalytic enantioselective Minisci-type addition to heteroarenes. *Science* **360,** 419–422 (2018).
19. Nuhant, P. *et al.* Visible-Light-Initiated Manganese Catalysis for C-H Alkylation of Heteroarenes: Applications and Mechanistic Studies. *Angew. Chem. Int. Ed.* **56,** 15309–15313 (2017).
20. Liu, P., Liu, W. & Li, C.-J. Catalyst-Free and Redox-Neutral Innate Trifluoromethylation and Alkylation of Aromatics Enabled by Light. *J. Am. Chem. Soc.* **139,** 14315–14321 (2017).
21. Ermanis, K. *et al.* A Computational and Experimental Investigation of the Origin of Selectivity in the Chiral Phosphoric Acid Catalyzed Enantioselective Minisci Reaction. *J. Am. Chem. Soc.* **142,** 21091–21101 (2020).
22. Matsui, J., Primer, D. & Molander, G. Metal-free C-H alkylation of heteroarenes with alkyltrifluoroborates: A general protocol for 1°, 2° and 3° alkylation. *Chem. Sci.* **8,** 3512–3522 (2017).
23. Li, G.-X. *et al.* Photoredox-mediated Minisci C-H alkylation of N-heteroarenes using boronic acids and hypervalent iodine. *Chem. Sci.* **7,** 6407–6412 (2016).
24. Jin, J. & MacMillan, D. Direct α-arylation of ethers through the combination of photoredox-mediated C-H functionalization and the minisci reaction. *Angew. Chem. Int. Ed.* **54,** 1565–1569 (2015).
25. Graham, M. *et al.* Development and Proof of Concept for a Large-Scale Photoredox Additive-Free Minisci Reaction. *Organic Process Research and Development* **25,** 57–67 (2021).
26. Dong, J., Yue, F., Song, H., Liu, Y. & Wang, Q. Visible-light-mediated photoredox minisci C-H alkylation with alkyl boronic acids using molecular oxygen as an oxidant. *Chem. Commun.* **56,** 12652–12655 (2020).

27. Rammal, F. *et al.* Visible-Light-Mediated C-H Alkylation of Pyridine Derivatives. *Org. Lett.* **22,** 7671–7675 (2020).

28. Ikarashi, G., Morofuji, T. & Kano, N. Terminal-oxidant-free photocatalytic C-H alkylations of heteroarenes with alkylsilicates as alkyl radical precursors. *Chem. Commun.* **56,** 10006–10009 (2020).

29. Dong, J., Wang, X., Song, H., Liu, Y. & Wang, Q. Photoredox-Catalyzed Redox-Neutral Minisci C-H Formylation of N-Heteroarenes. *Advanced Synthesis and Catalysis* **362,** 2155–2159 (2020).

30. Li, T. *et al.* Three-Component Minisci Reaction with 1,3-Dicarbonyl Compounds Induced by Visible Light. *Org. Lett.* **22,** 2386–2390 (2020).

31. Zidan, M., Morris, A., McCallum, T. & Barriault, L. The Alkylation and Reduction of Heteroarenes with Alcohols Using Photoredox Catalyzed Hydrogen Atom Transfer via Chlorine Atom Generation. *Eur. J. Org. Chem.* **2020,** 1453–1458 (2020).

32. Ji, X. *et al.* LiBr-promoted photoredox neutral Minisci hydroxyalkylations of quinolines with aldehydes. *Green Chem.* **22,** 8233–8237 (2020).

33. Perkins, J., Schubert, J., Streckfuss, E., Balsells, J. & ElMarrouni, A. Photoredox Catalysis for Silyl-Mediated C–H Alkylation of Heterocycles with Non-Activated Alkyl Bromides. *Eur. J. Org. Chem.* **2020,** 1515–1522 (2020).

34. Jian, Y., Chen, M., Yang, C. & Xia, W.-J. Minisci-Type C–H Cyanoalkylation of Heteroarenes Through N–O/C–C Bonds Cleavage. *Eur. J. Org. Chem.* **2020,** 1439–1442 (2020).

35. Li, X. *et al.* Complementary oxidative generation of iminyl radicals from α-imino-oxy acids: Silver-catalyzed c-h cyanoalkylation of heterocycles and quinones. *Journal of Organic Chemistry* **85,** 2504–2511 (2020).

36. Laha, J., Kaur Hunjan, M., Hegde, S. & Gupta, A. Aroylation of Electron-Rich Pyrroles under Minisci Reaction Conditions. *Org. Lett.* **22,** 1442–1447 (2020).

37. Xie, X., Zhang, Y., Hao, J. & Wan, W. Ag-Catalyzed minisci C-H difluoromethylarylation of N-heteroarenes. *Org. Biomol. Chem.* **18,** 400–404 (2020).

38. Wang, Z., Ji, X., Han, T., Deng, G.-J. & Huang, H. LiBr-Promoted Photoredox Minisci-Type Alkylations of Quinolines with Ethers. *Advanced Synthesis and Catalysis* **361,** 5643–5647 (2019).

39. Vijeta, A. & Reisner, E. Carbon nitride as a heterogeneous visible-light photocatalyst for the Minisci reaction and coupling to H2 production. *Chem. Commun.* **55,** 14007–14010 (2019).

40. Dou, G.-Y., Jiang, Y.-Y., Xu, K. & Zeng, C.-C. Electrochemical Minisci-type trifluoromethylation of electron-deficient heterocycles mediated by bromide ions. *Organic Chemistry Frontiers* **6,** 2392–2397 (2019).

41. Sutherland, D. R., Veguillas, M., Oates, C. L. & Lee, A.-L. Metal-, photocatalyst-, and light-free, late-stage C–H alkylation of heteroarenes and 1, 4-quinones using carboxylic acids. *Org. Lett.* **20,** 6863–6867 (2018).

42. Bosset, C. *et al.* Minisci-Photoredox-Mediated α-Heteroarylation of N-Protected Secondary Amines: Remarkable Selectivity of Azetidines. *Org. Lett.* **20,** 6003–6006 (2018).

43. Fuse, H. *et al.* Photocatalytic redox-neutral hydroxyalkylation of: N -heteroaromatics with aldehydes. *Chem. Sci.* **11,** 12206–12211 (2020).

44. Sherwood, T., Li, N., Yazdani, A. & Dhar, T. Organocatalyzed, Visible-Light Photoredox-Mediated, One-Pot Minisci Reaction Using Carboxylic Acids via N-(Acyloxy)phthalimides. *Journal of Organic Chemistry* **83,** 3000–3012 (2018).

45. Zhang, L. & Liu, Z.-Q. Molecular Oxygen-Mediated Minisci-Type Radical Alkylation of Heteroarenes with Boronic Acids. *Org. Lett.* **19,** 6594–6597 (2017).

46. Galloway, J., Mai, D. & Baxter, R. Silver-Catalyzed Minisci Reactions Using Selectfluor as a Mild Oxidant. *Org. Lett.* **19,** 5772–5775 (2017).

47. Wang, Q.-Q. *et al.* Electrocatalytic Minisci Acylation Reaction of N-Heteroarenes Mediated by NH4I. *Org. Lett.* **19,** 5517–5520 (2017).

48. Tang, R.-J., Kang, L. & Yang, L. Metal-Free Oxidative Decarbonylative Coupling of Aliphatic Aldehydes with Azaarenes: Successful Minisci-Type Alkylation of Various Heterocycles. *Advanced Synthesis and Catalysis* **357,** 2055–2060 (2015).

49. Siddaraju, Y., Lamani, M. & Prabhu, K. A transition metal-free Minisci reaction: Acylation of isoquinolines, quinolines, and quinoxaline. *Journal of Organic Chemistry* **79,** 3856–3865 (2014).

50. Stephenson, C., McClain, E., Monos, T., Mori, M. & Beatty, J. Design and implementation of a catalytic electron donor-acceptor complex platform for radical trifluoromethylation and alkylation. *ACS Catalysis* **10,** 12636–12641 (2020).

51. Wang, Q., Duan, J., Tang, P., Chen, G. & He, G. Synthesis of non-classical heteroaryl C-glycosides via Minisci-type alkylation of N-heteroarenes with 4-glycosyl-dihydropyridines. *Science China Chemistry* **63,** 1613–1618 (2020).

52. Dong, J. *et al.* Visible-light-mediated minisci C-H alkylation of heteroarenes with 4-alkyl-1,4-dihydropyridines using O2as an oxidant. *Green Chem.* **22,** 5599–5604 (2020).

53. Wang, Z., Liu, Q., Ji, X., Deng, G.-J. & Huang, H. Bromide-promoted visible-light-induced reductive minisci reaction with aldehydes. *ACS Catalysis* **10,** 154–159 (2020).

54. Ding, H., Xu, K. & Zeng, C.-C. Nickel-catalyzed electrochemical Minisci acylation of aromatic N-heterocycles with α-keto acids via ligand-to-metal electron transfer pathway. *Journal of Catalysis* **381,** 38–43 (2020).

55. Miyaura, N. & Suzuki, A. Palladium-catalyzed cross-coupling reactions of organoboron compounds. *Chem. Rev.* **95,** 2457–2483 (1995).

56. Nicolaou, K., Bulger, P. G. & Sarlah, D. Palladium-catalyzed cross-coupling reactions in total synthesis. *Angew. Chem. Int. Ed.* **44,** 4442–4489 (2005).
