# Supplementary material for: Identifying opportunities for late-stage C-H alkylation with high-throughput experimentation and in silico reaction screening
Source: Commun Chem. 2023 Nov 20;6:256. doi: 10.1038/s42004-023-01047-5 (PMC10661846; doi:10.1038/s42004-023-01047-5)
Supplement: Supplementary file 2 — Description of Additional Supplementary Files [file 42004_2023_1047_MOESM2_ESM.pdf]

Description of Additional Supplementary Files

File name: Supplementary Data 1

Description: pdf file containing NMR spectra

File name: Supplementary Data 2

Description: tsv file containing all reactions i.e., literature, decoy and experimental data

File name: Supplementary Data 3

Description: tsv file containing reactions from literature.

File name: Supplementary Data 4

Description: .tsv file containing experimental reaction data

File name: Supplementary Data 5

Description: .tsv file containing reactions conducted to validate the literature data. These reactions were excluded in machine learning model training.

File name: Supplementary Data 6

Description: .tsv file containing decoy reactions

File name: Supplementary Data 7

Description: .tsv file containing all investigated carboxylic acids

File name: Supplementary Data 8

Description: .tsv file containing all investigated N -hetero arenes.
