# Supplementary material for: Identifying opportunities for late-stage C-H alkylation with high-throughput experimentation and in silico reaction screening
Source: Commun Chem. 2023 Nov 20;6:256. doi: 10.1038/s42004-023-01047-5 (PMC10661846; doi:10.1038/s42004-023-01047-5)
Supplement: Supplementary file 3 — Supplementary Data 1 [file 42004_2023_1047_MOESM3_ESM.pdf]

Supplementary Data:
Identifying opportunities for late-stage C-H alkylation with *in silico* reaction screening and high-throughput experimentation

David F. Nippa[1,2,†], Kenneth Atz[1,†], Alex T. Müller[1], Jens Wolfard[1], Clemens Isert[3], Martin Binder[1], Oliver Scheidegger[1], David B. Konrad[2,*], Uwe Grether[1,*], Rainer E. Martin[1,*] & Gisbert Schneider[3,*]

[1]Roche Pharma Research and Early Development (pRED), Roche Innovation Center Basel, F. Hoffmann-La Roche Ltd., Grenzacherstrasse 124, 4070 Basel, Switzerland.
[2]Department of Pharmacy, Ludwig-Maximilians-Universität München, Butenandtstrasse 5, 81377 Munich, Germany.
[3]ETH Zurich, Department of Chemistry and Applied Biosciences, Vladimir-Prelog-Weg 4, 8093 Zurich, Switzerland.
[4]ETH Singapore SEC Ltd, 1 CREATE Way, #06-01 CREATE Tower, Singapore, Singapore.
† These authors contributed equally to this work.
∗ To whom correspondence should be addressed.
E-mail: david.konrad@cup.lmu.de, uwe.grether@roche.com, rainer_e.martin@roche.com, gisbert@ethz.ch

1 NMR spectra

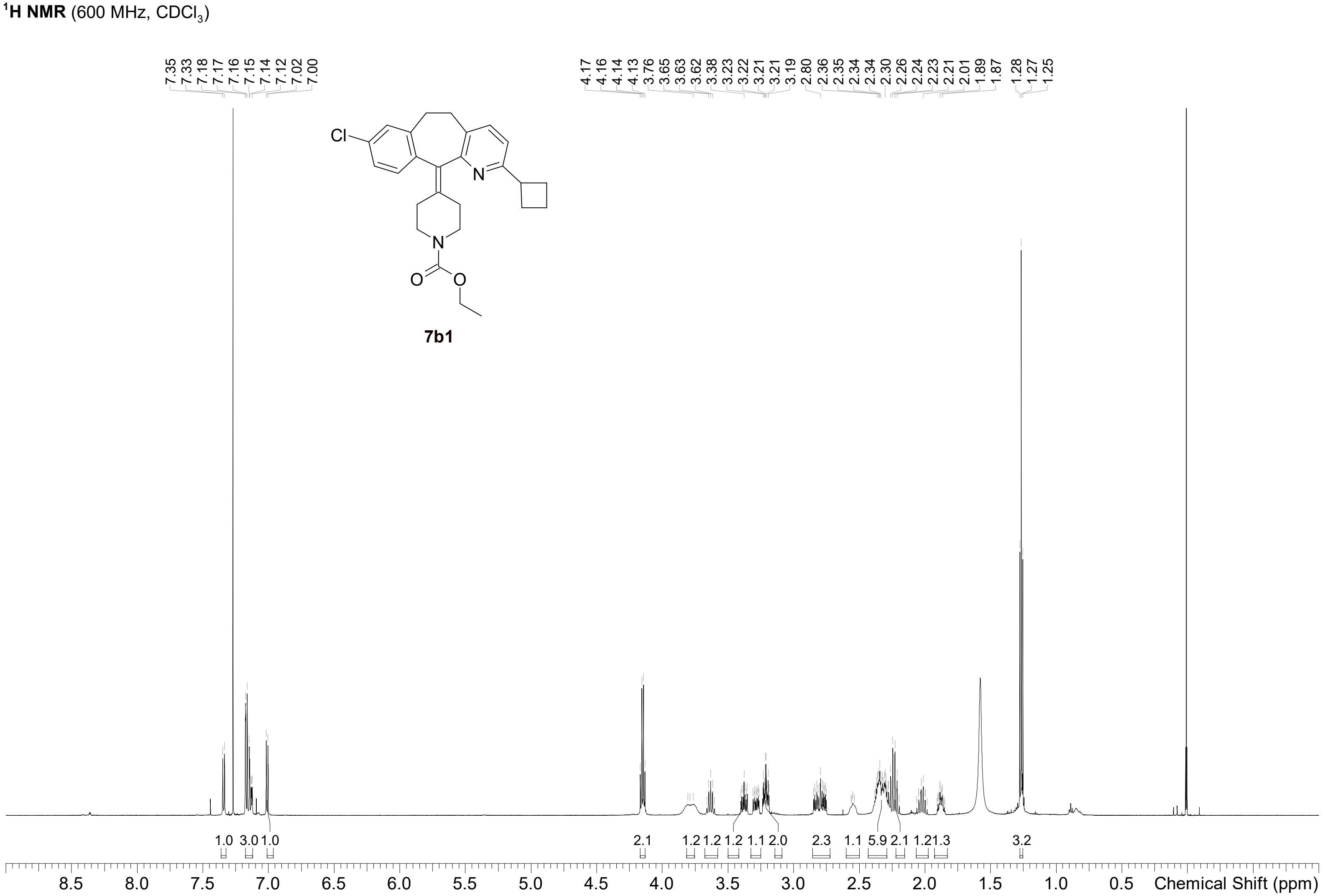

Figure 1: **7b1**, ^{1}H-NMR spectrum.

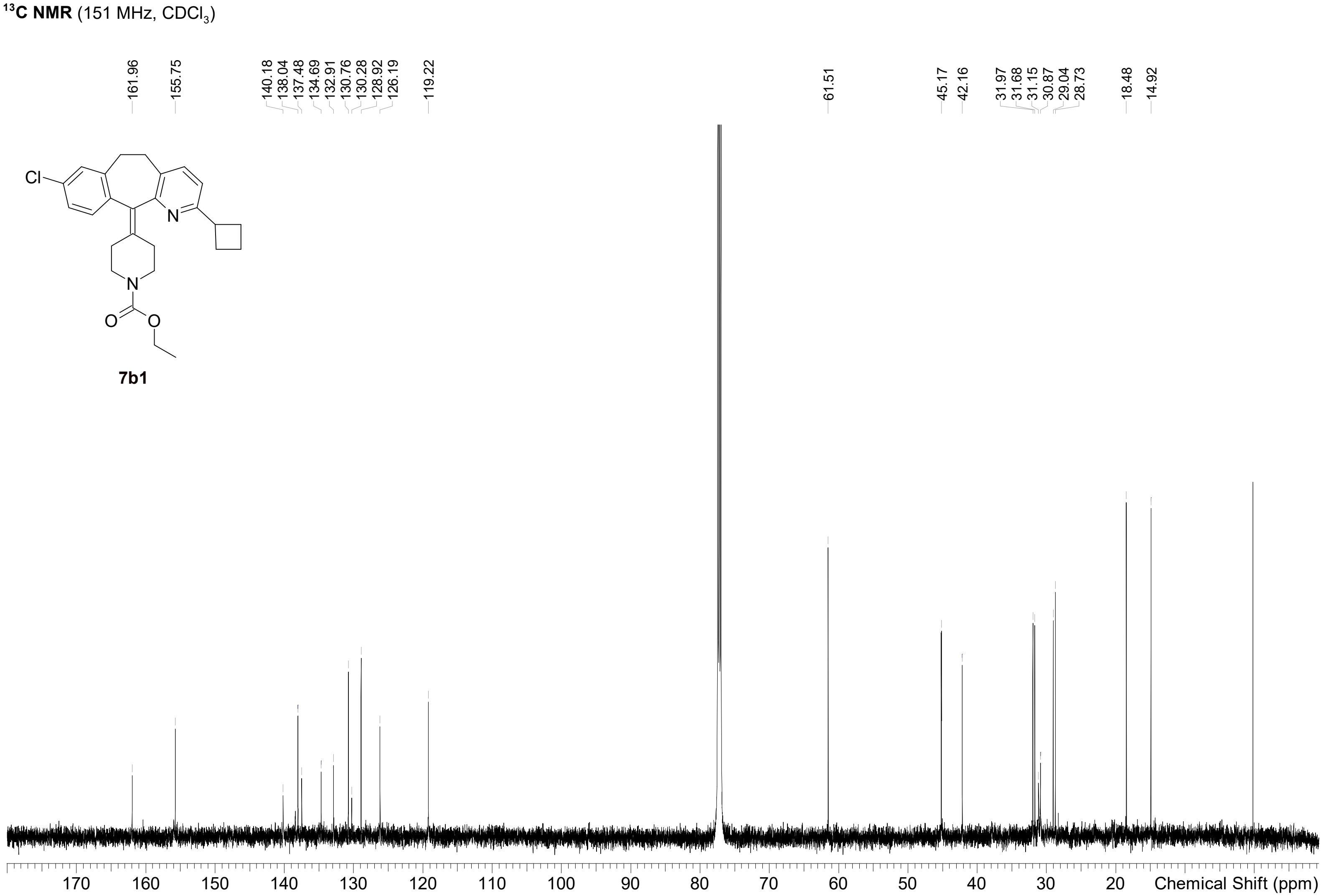

Figure 2: **7b1**, ^{13}C-NMR spectrum.

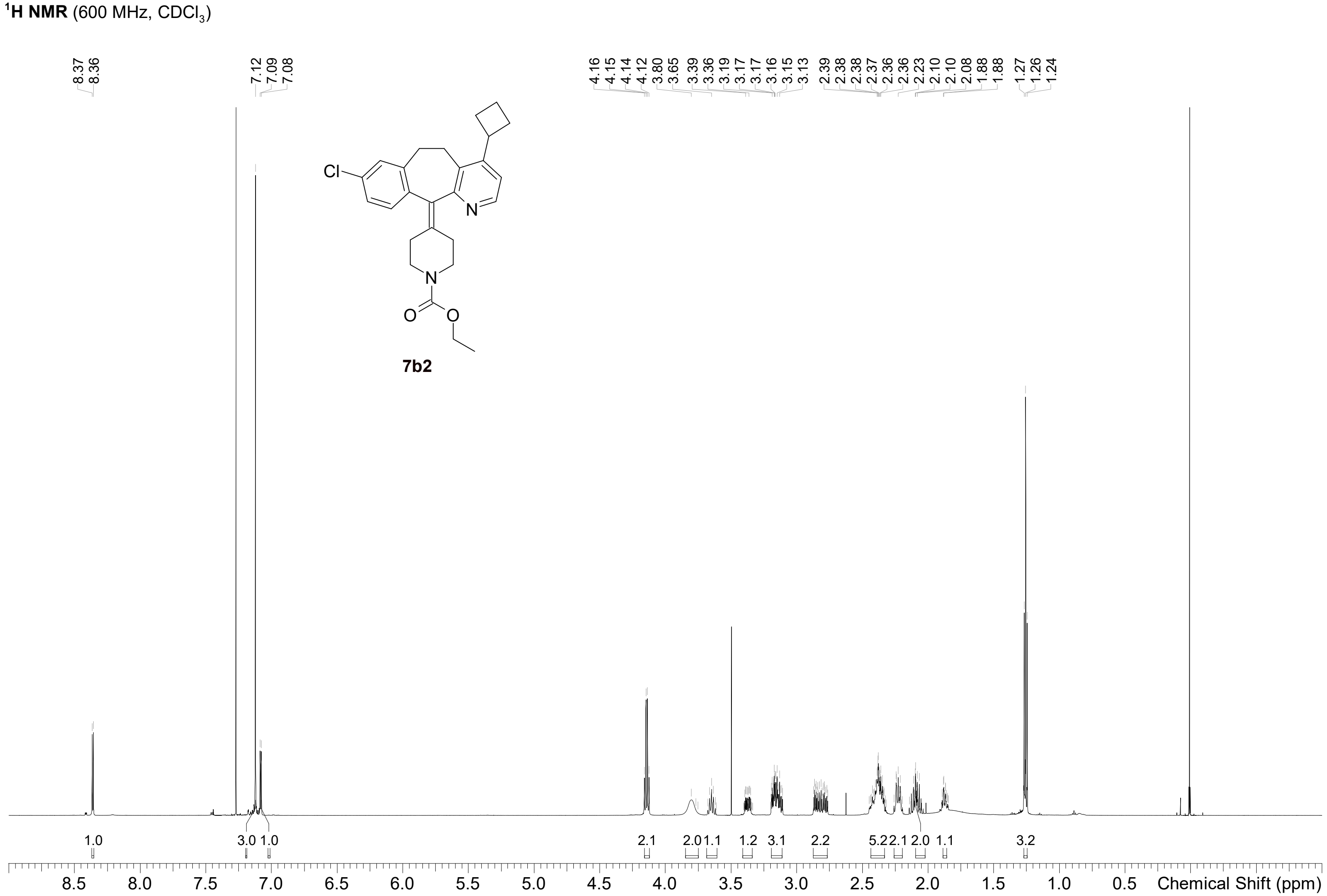

Figure 3: **7b2**, ^{1}H-NMR spectrum.

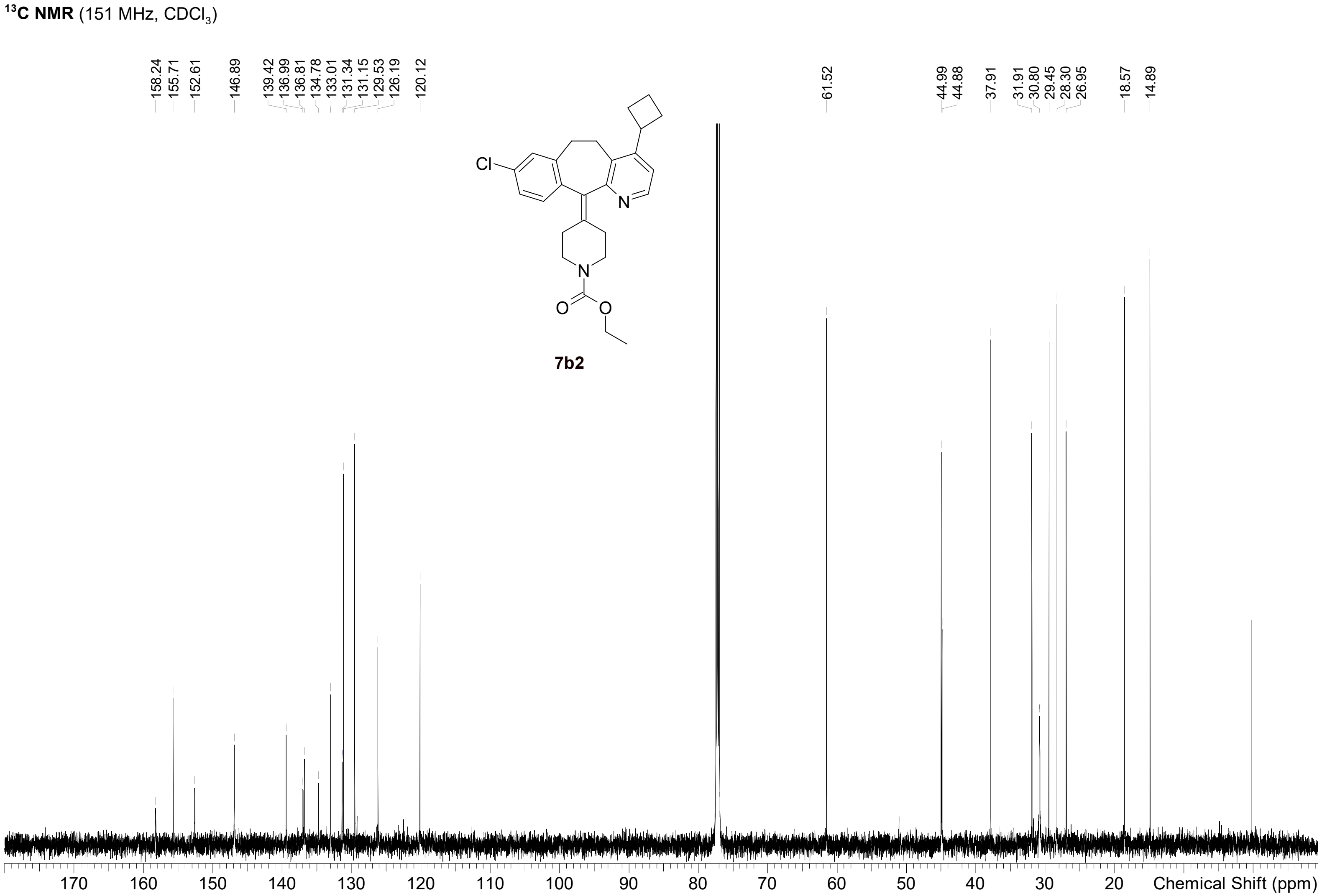

Figure 4: **7b2**, ^{13}C-NMR spectrum.

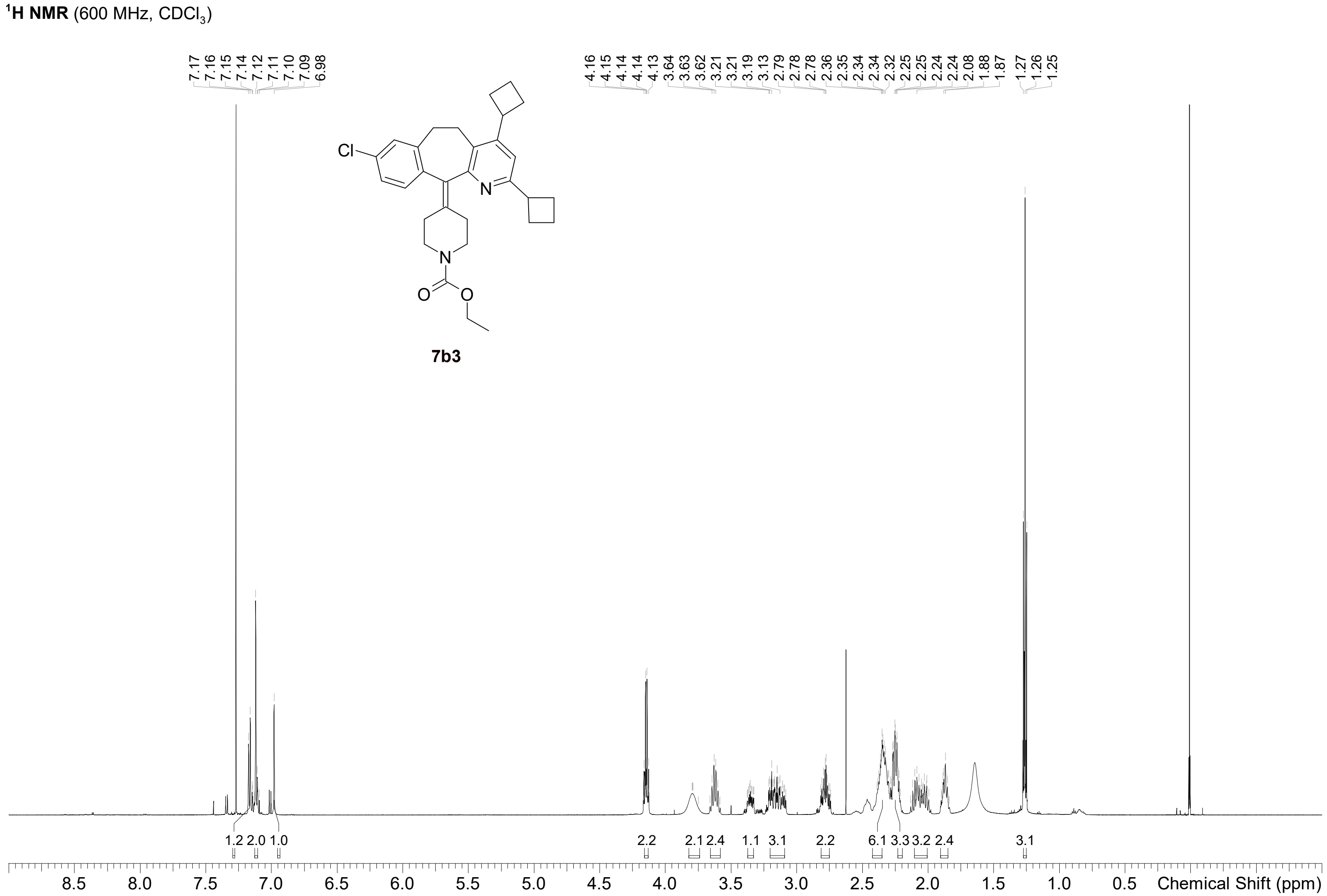

Figure 5: **7b3**, ^{1}H-NMR spectrum.

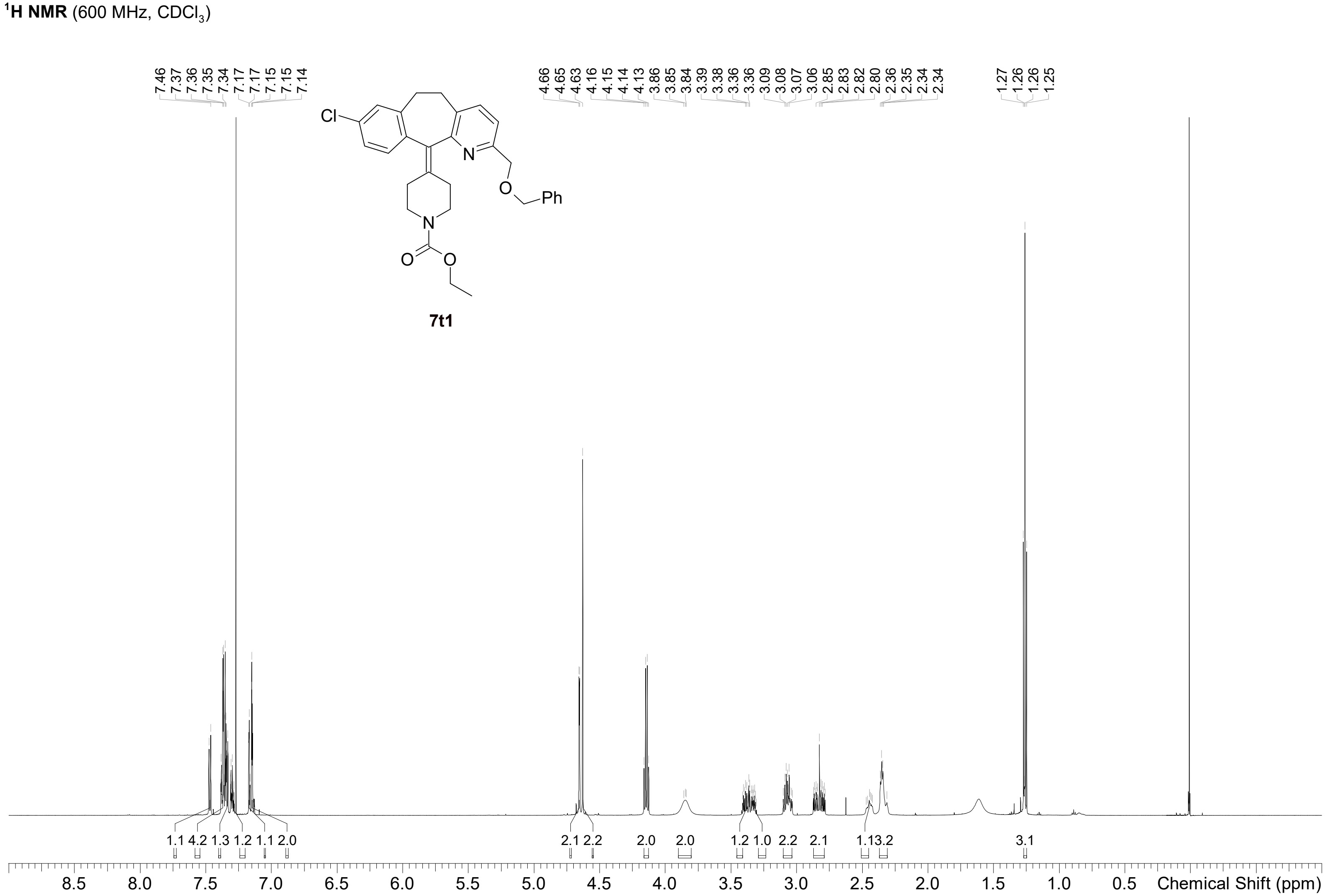

Figure 6: **7t1**, ^{1}H-NMR spectrum.

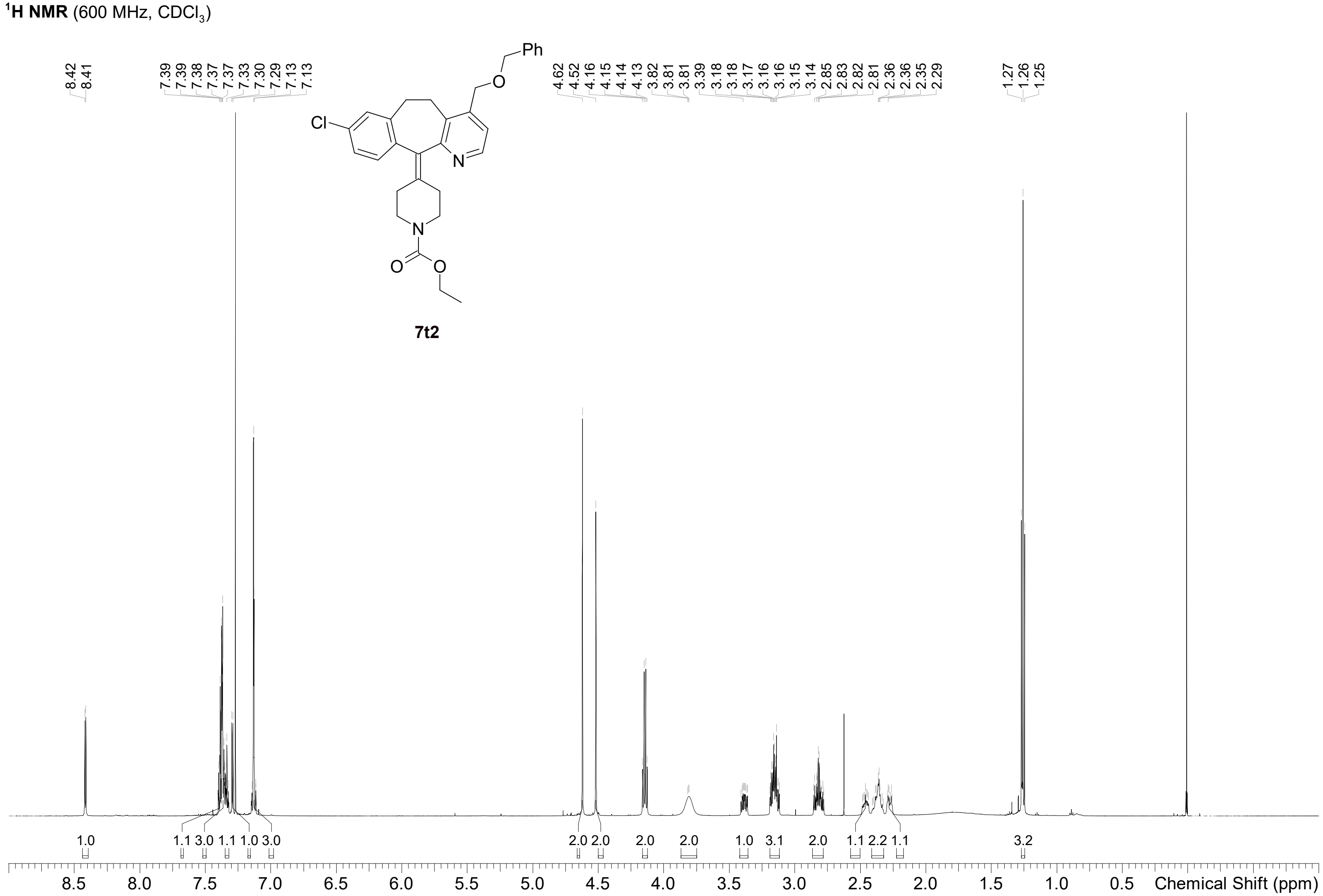

Figure 7: **7t2**, ^{1}H-NMR spectrum.

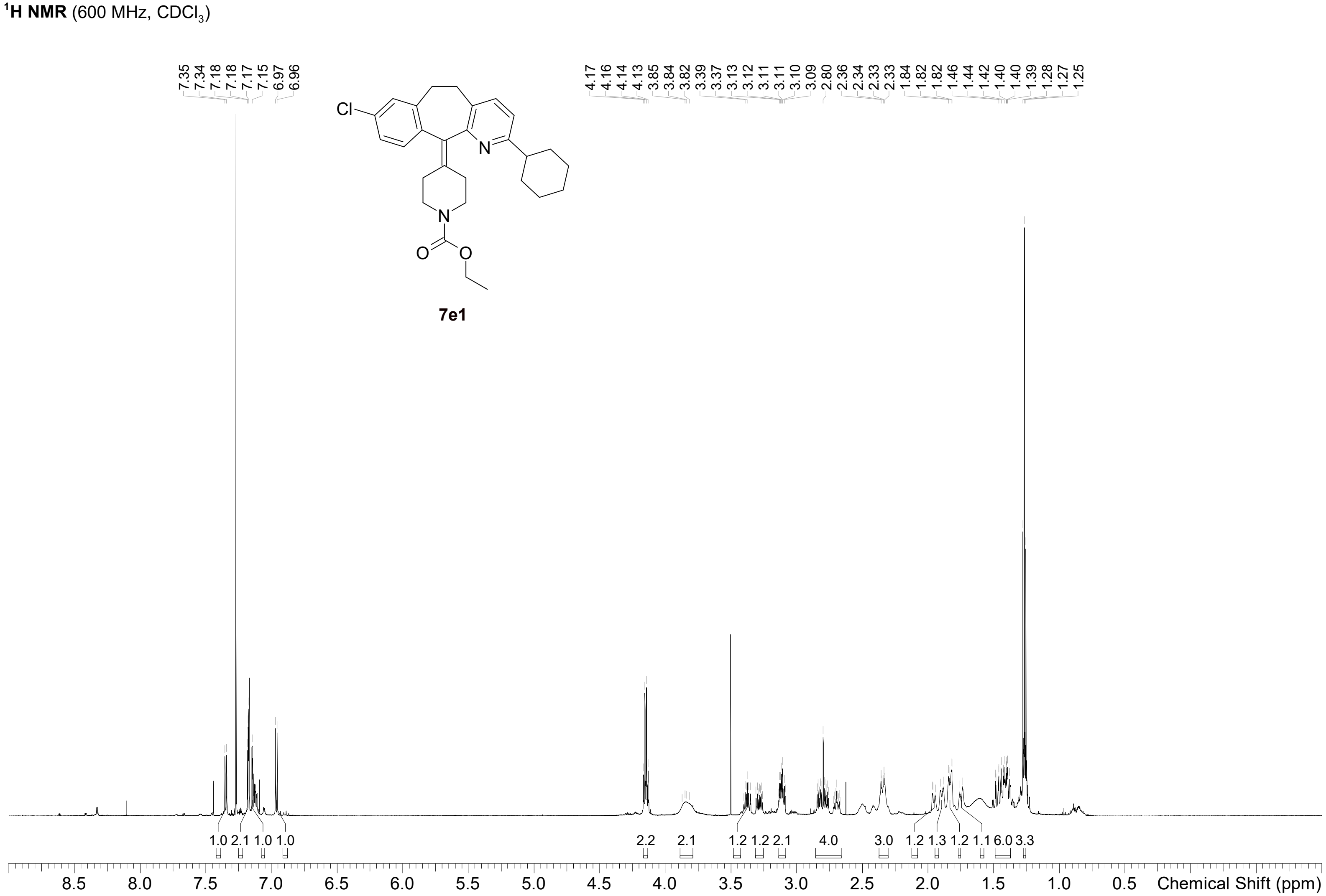

Figure 8: **7e1**, ^{1}H-NMR spectrum.

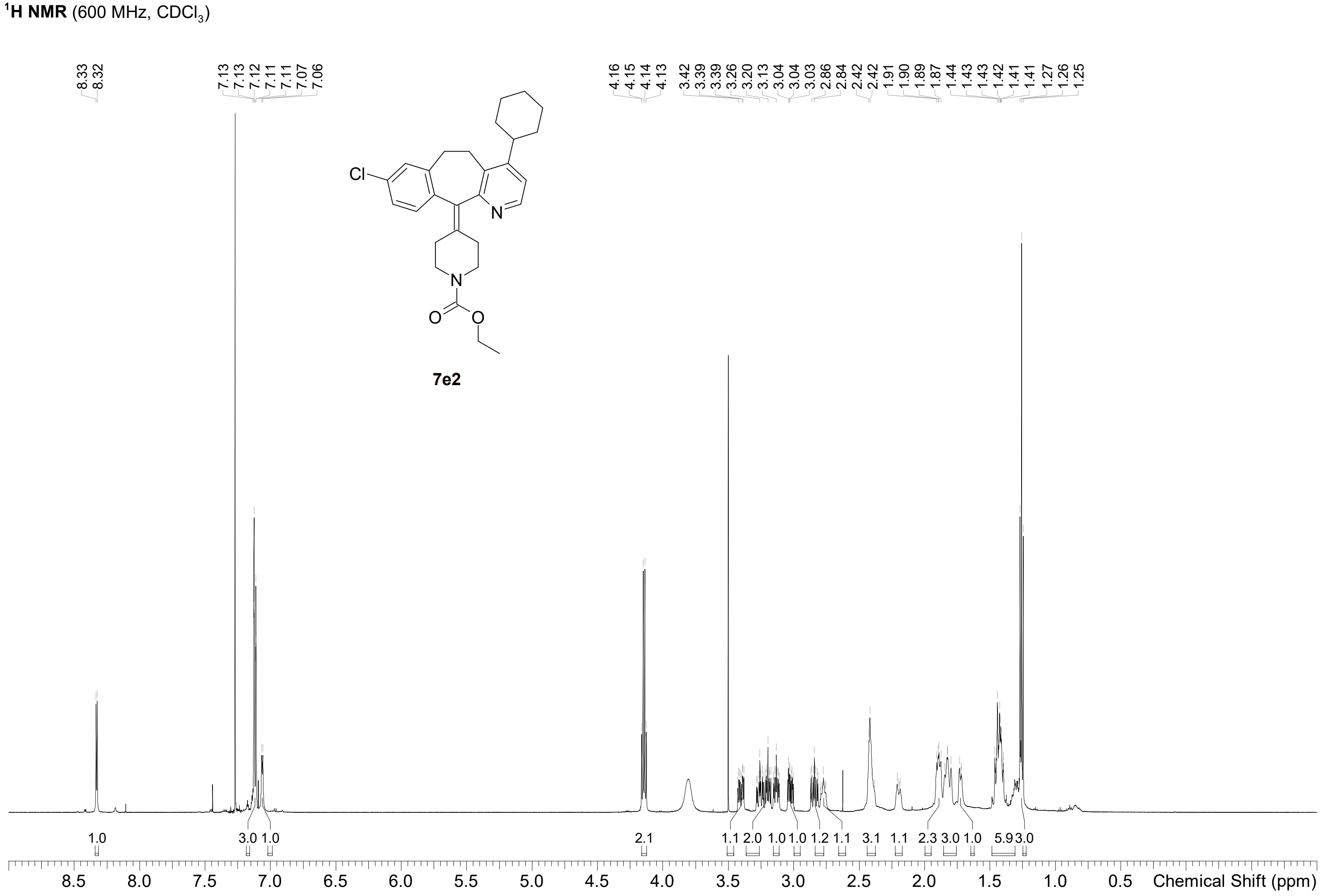

Figure 9: **7e2**, ^{1}H-NMR spectrum.

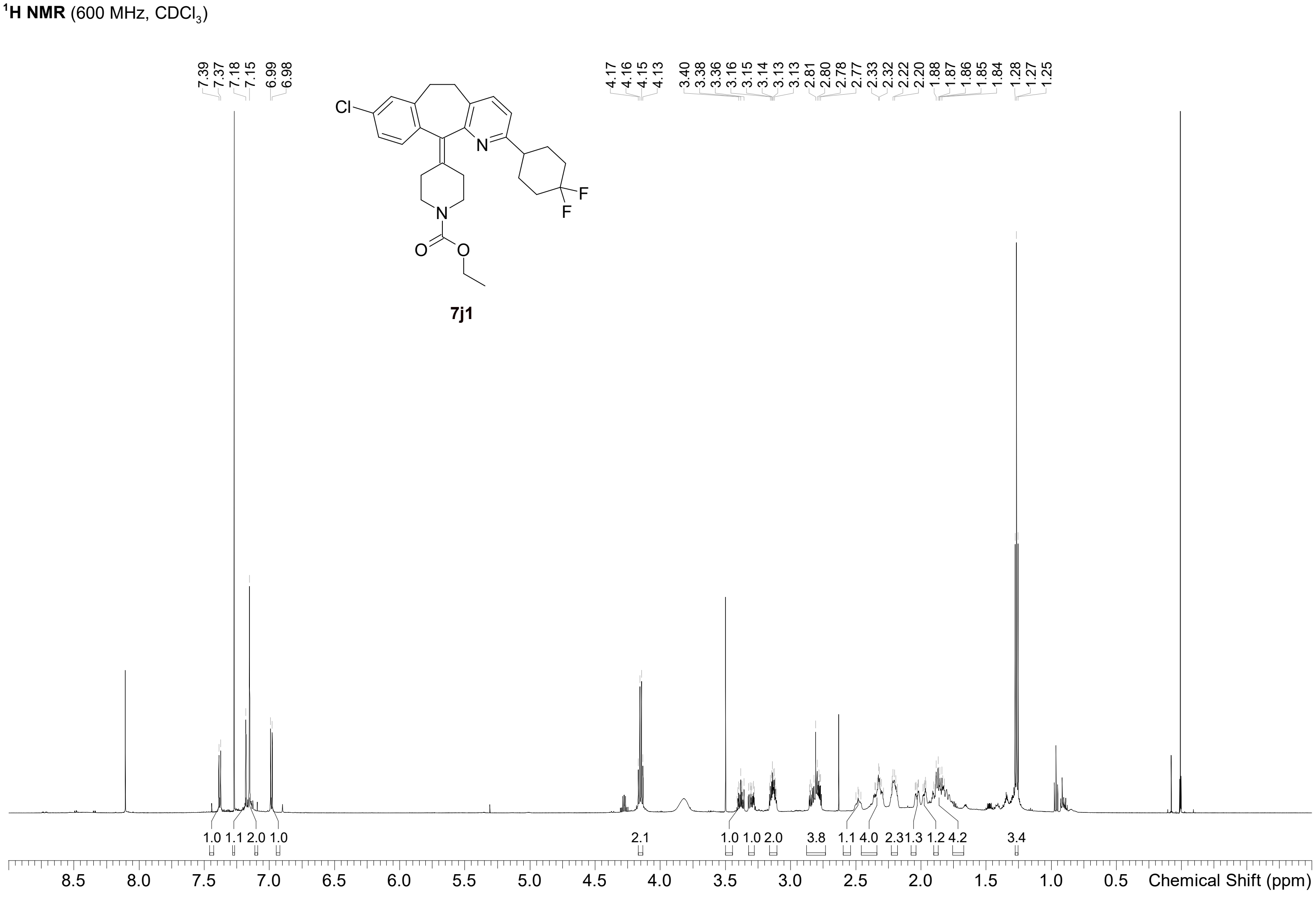

Figure 10: **7j1**, ^{1}H-NMR spectrum.

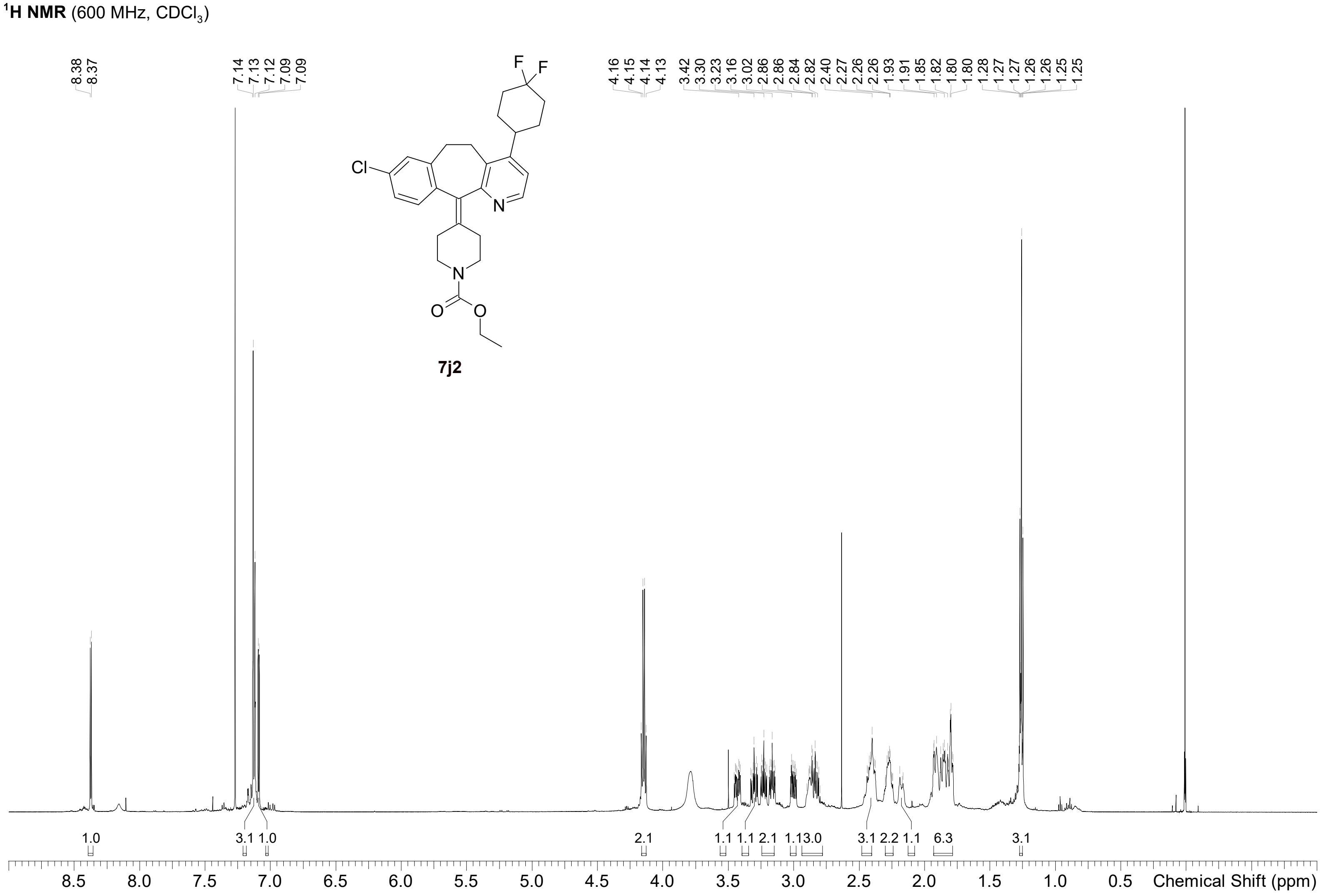

Figure 11: **7j2**, ^{1}H-NMR spectrum.

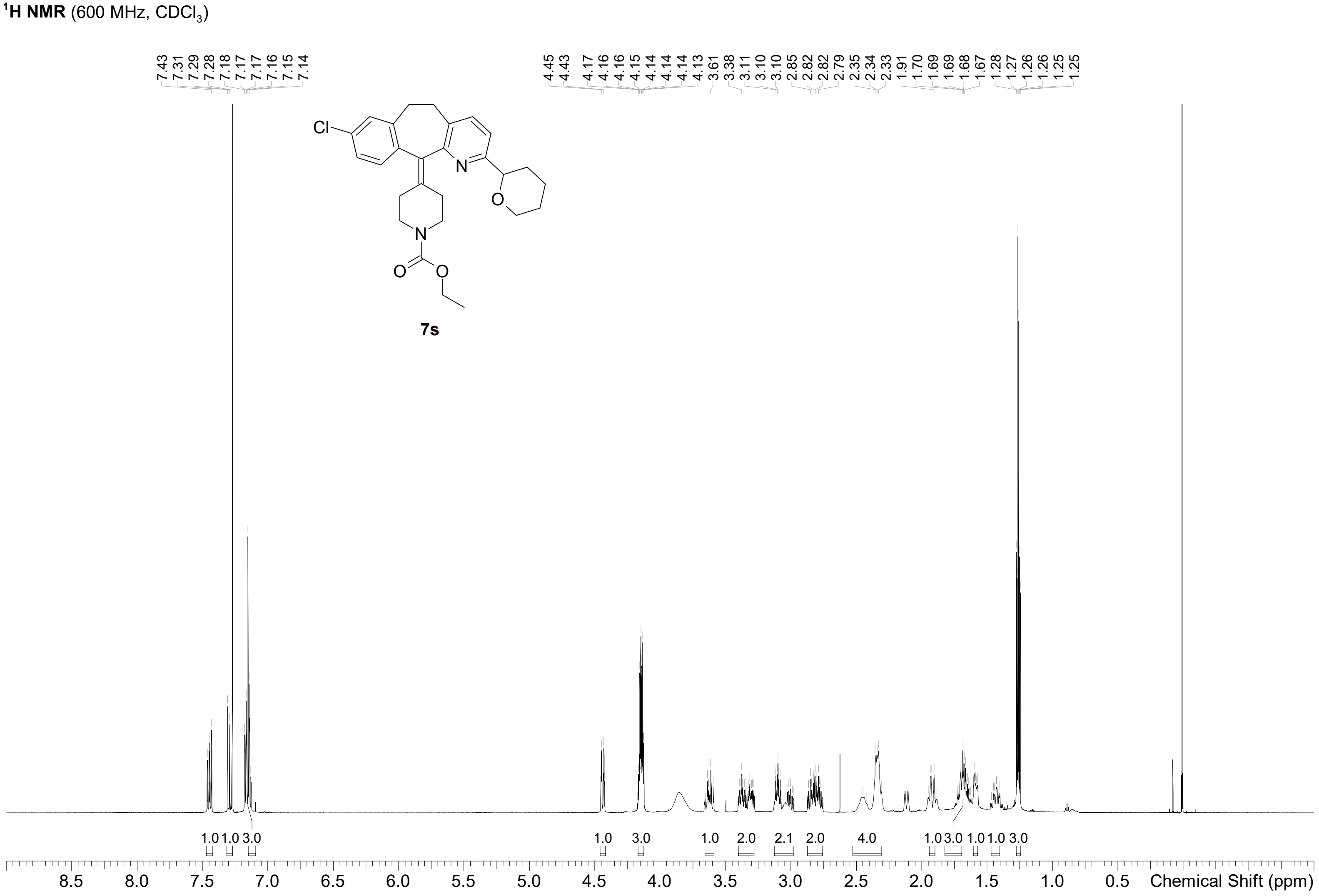

Figure 12: **7s**, ^{1}H-NMR spectrum.

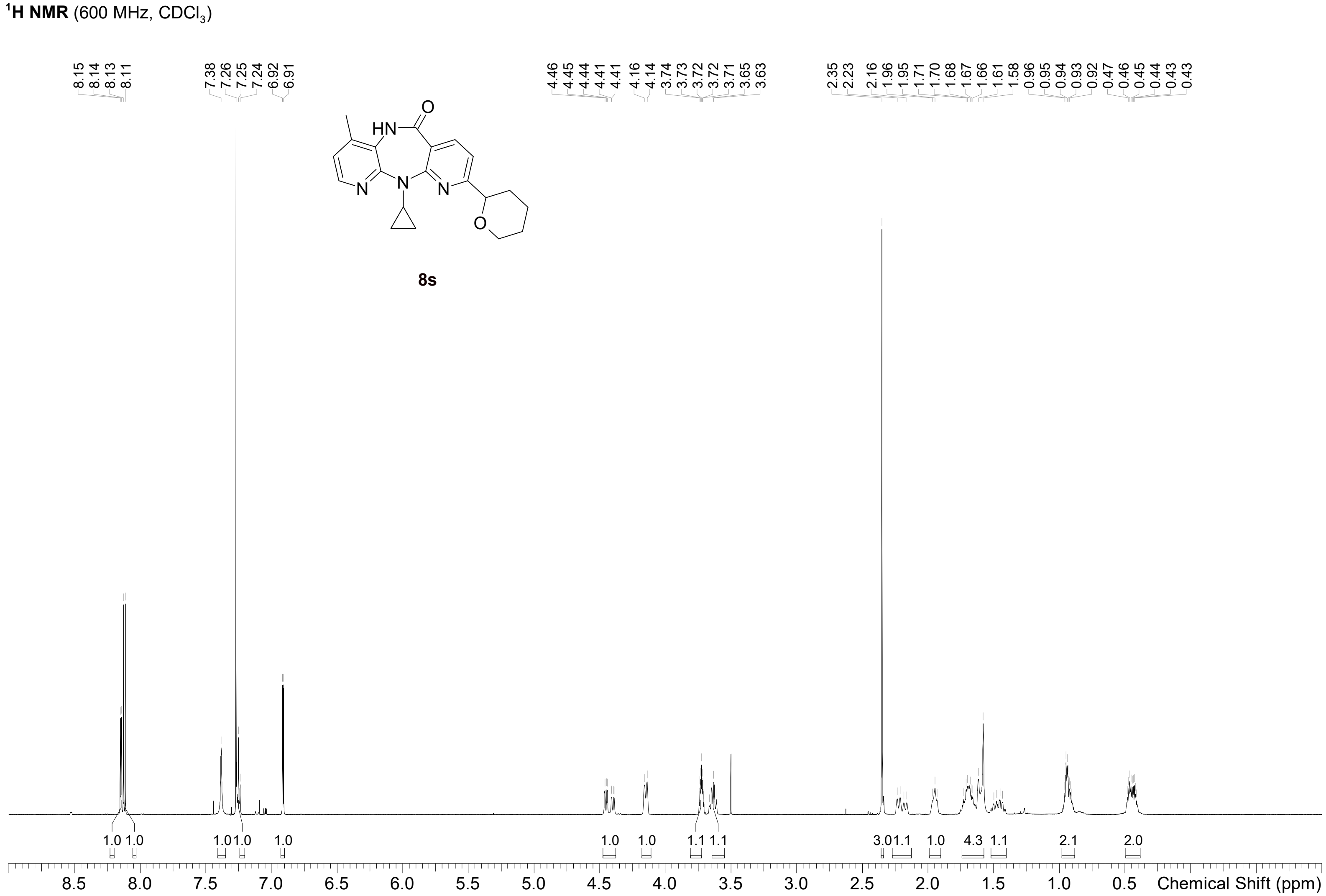

Figure 13: **8s**, ^{1}H-NMR spectrum.

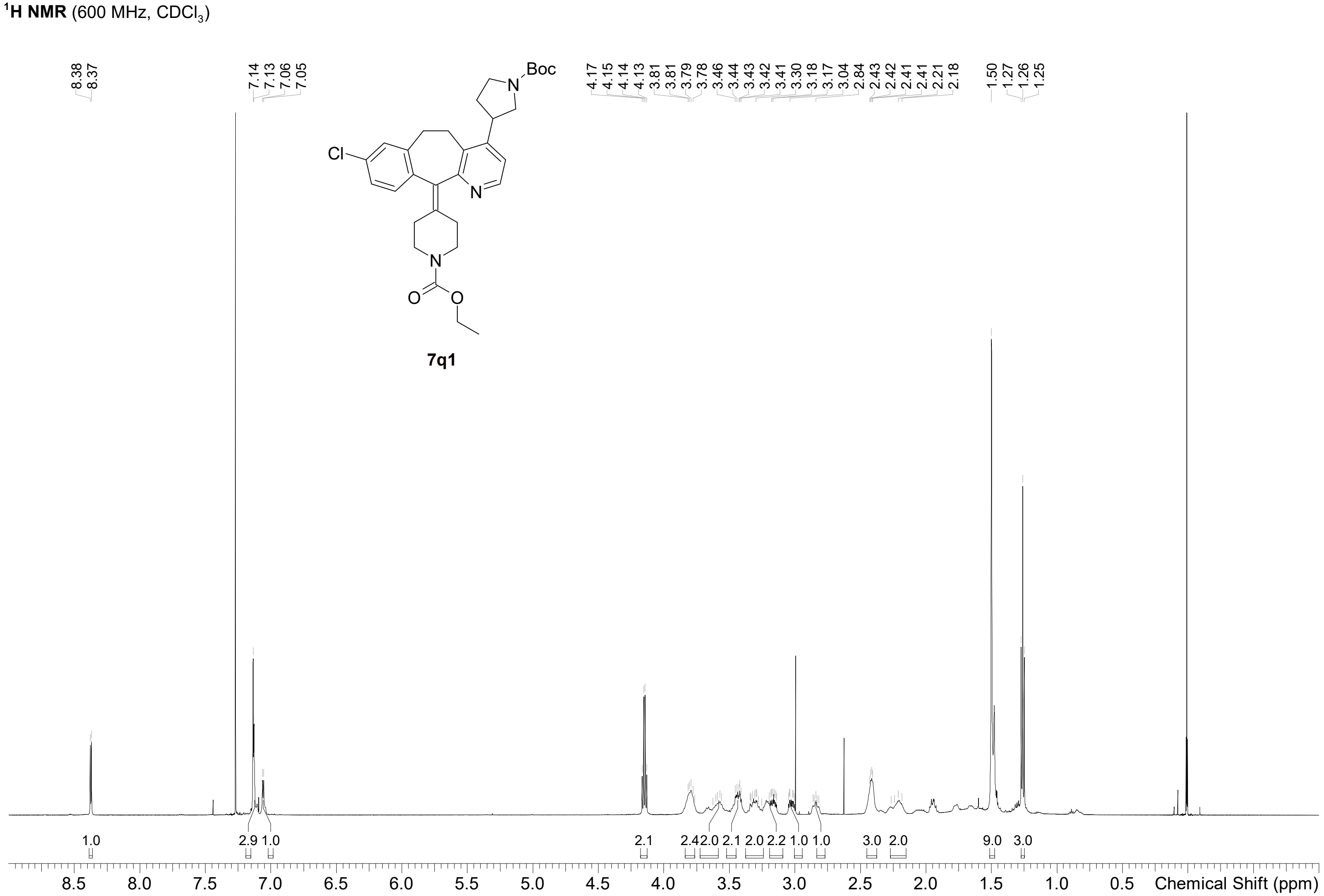

Figure 14: **7q1**, ^{1}H-NMR spectrum.

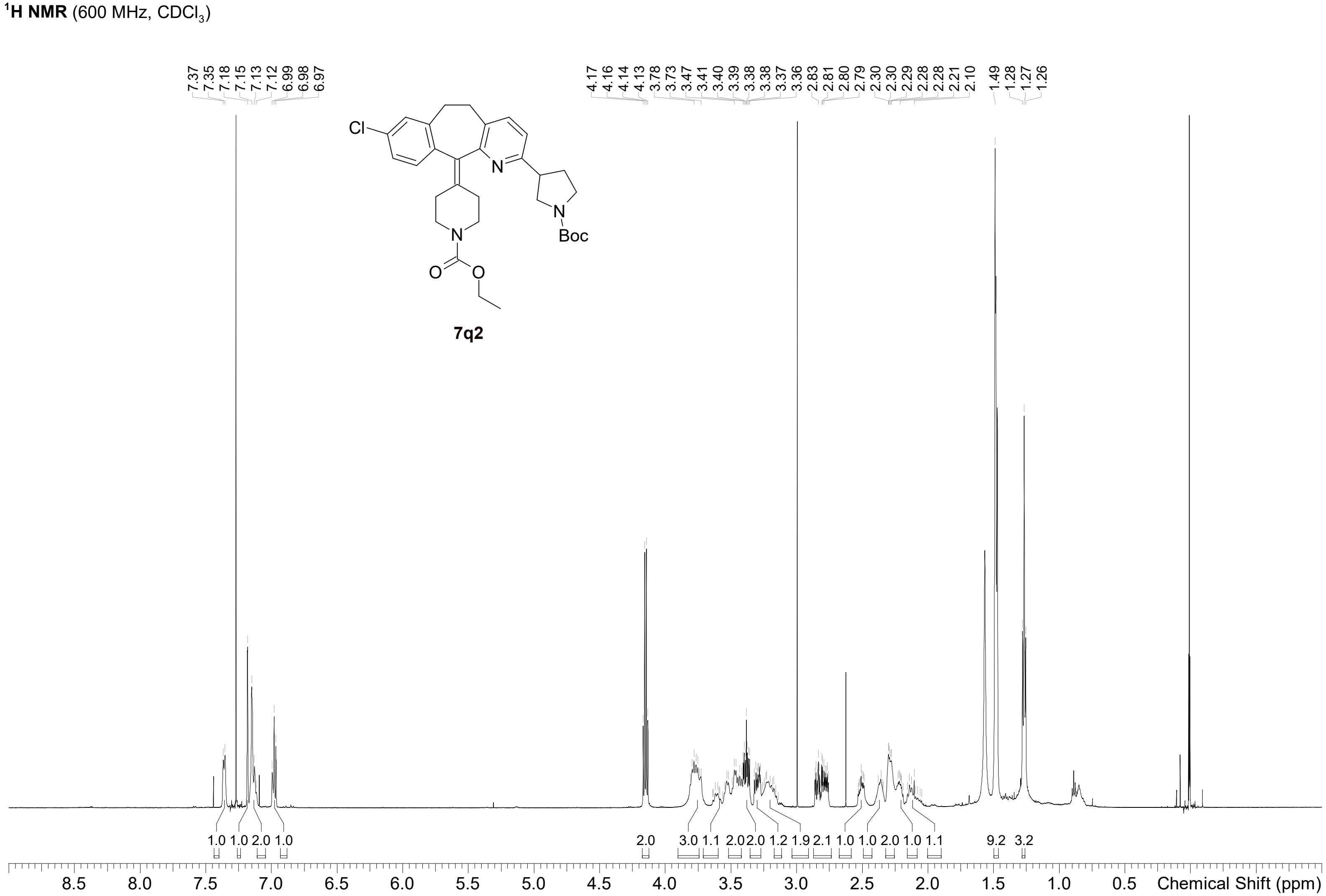

Figure 15: **7q2**, ^{1}H-NMR spectrum.

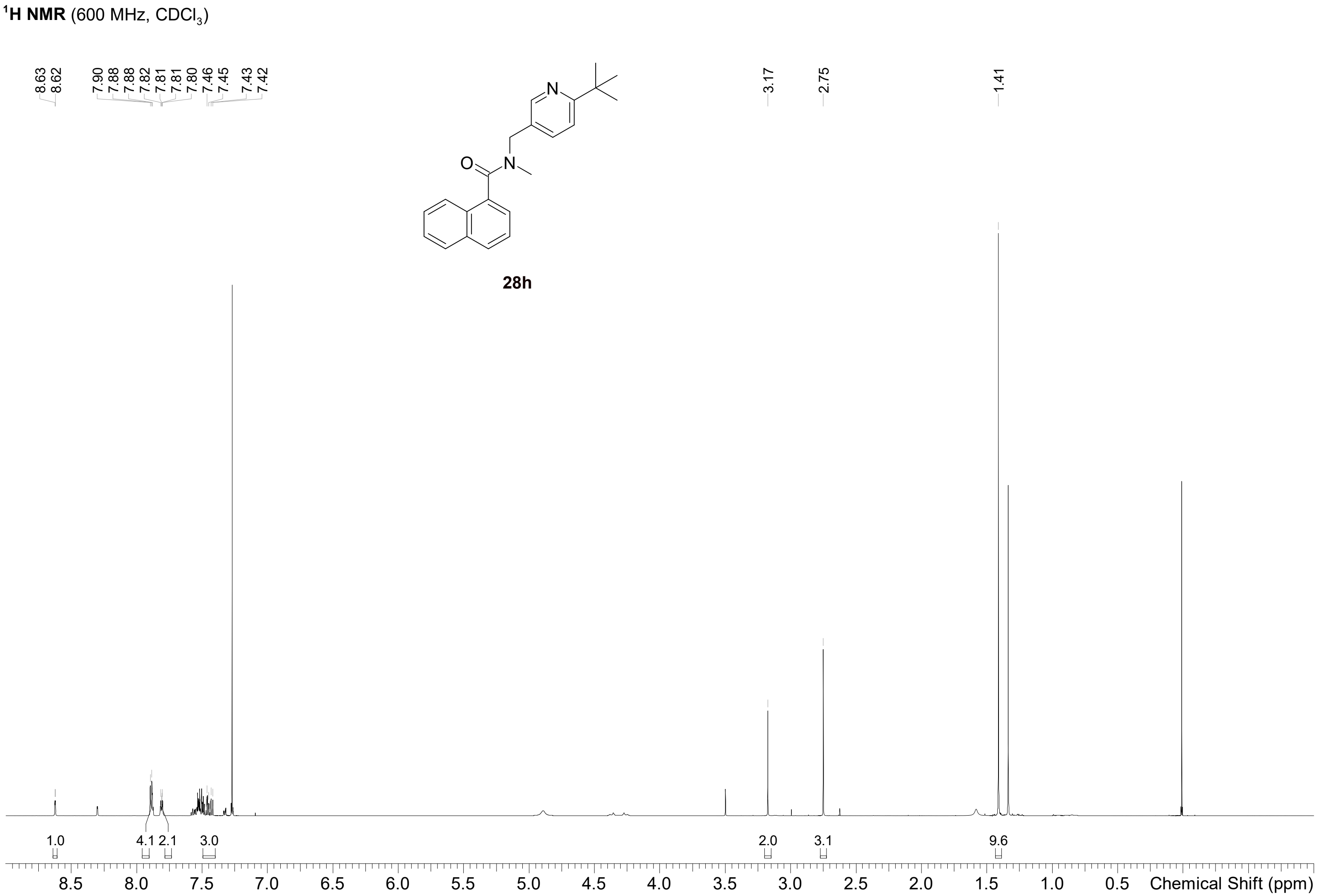

Figure 16: **28h**, ^{1}H-NMR spectrum.

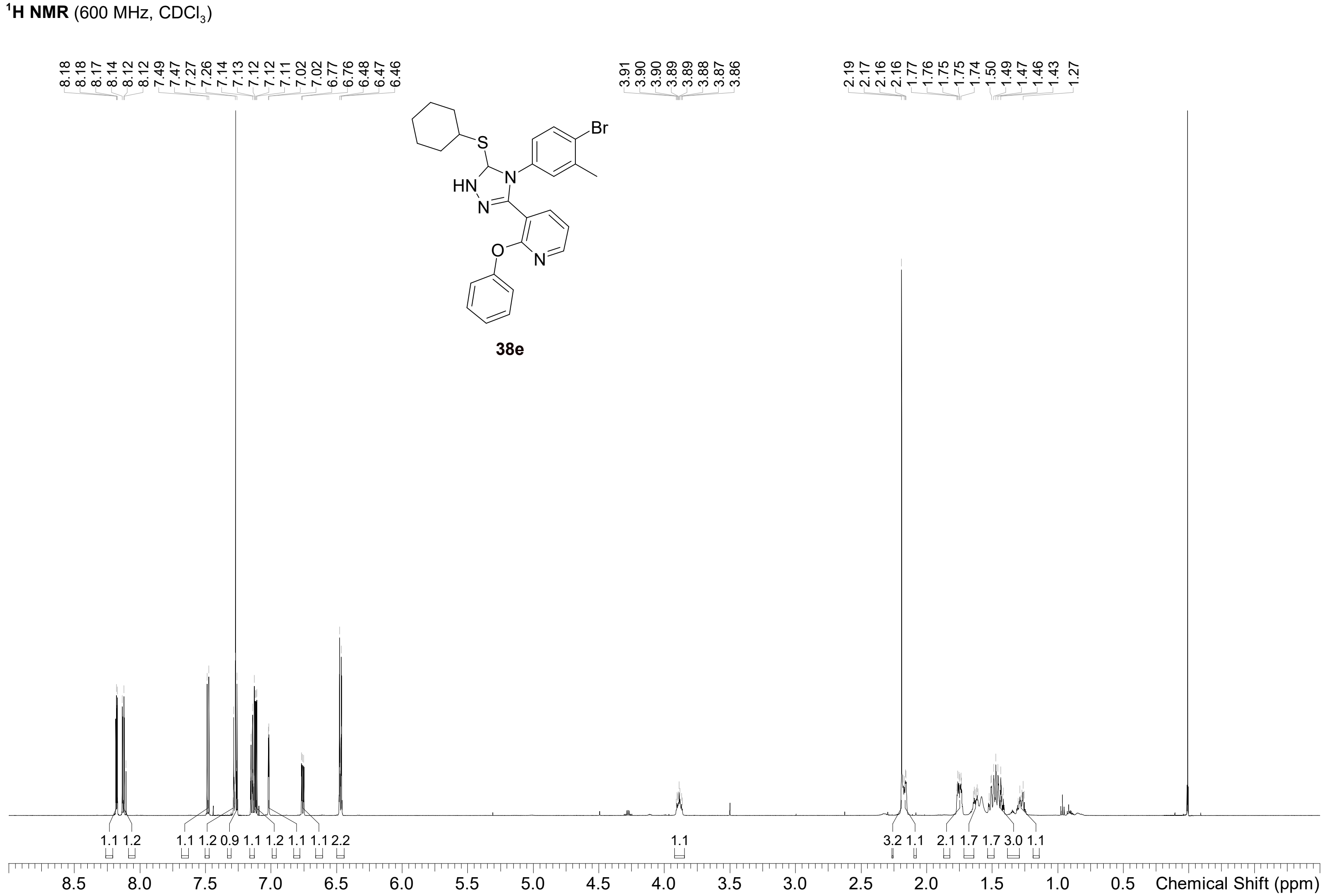

Figure 17: **38e**, ^{1}H-NMR spectrum.

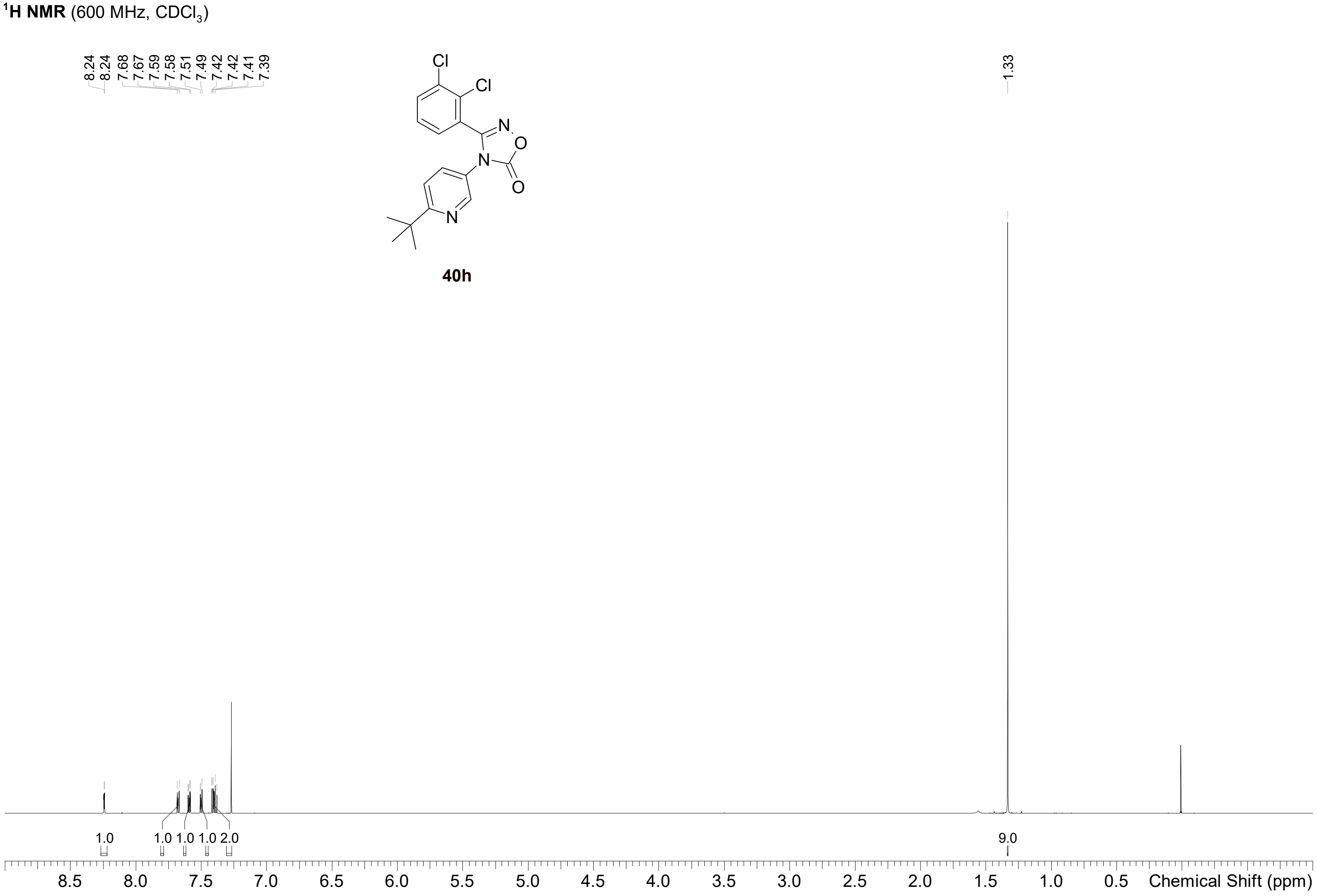

Figure 18: **40h**, ^{1}H-NMR spectrum.

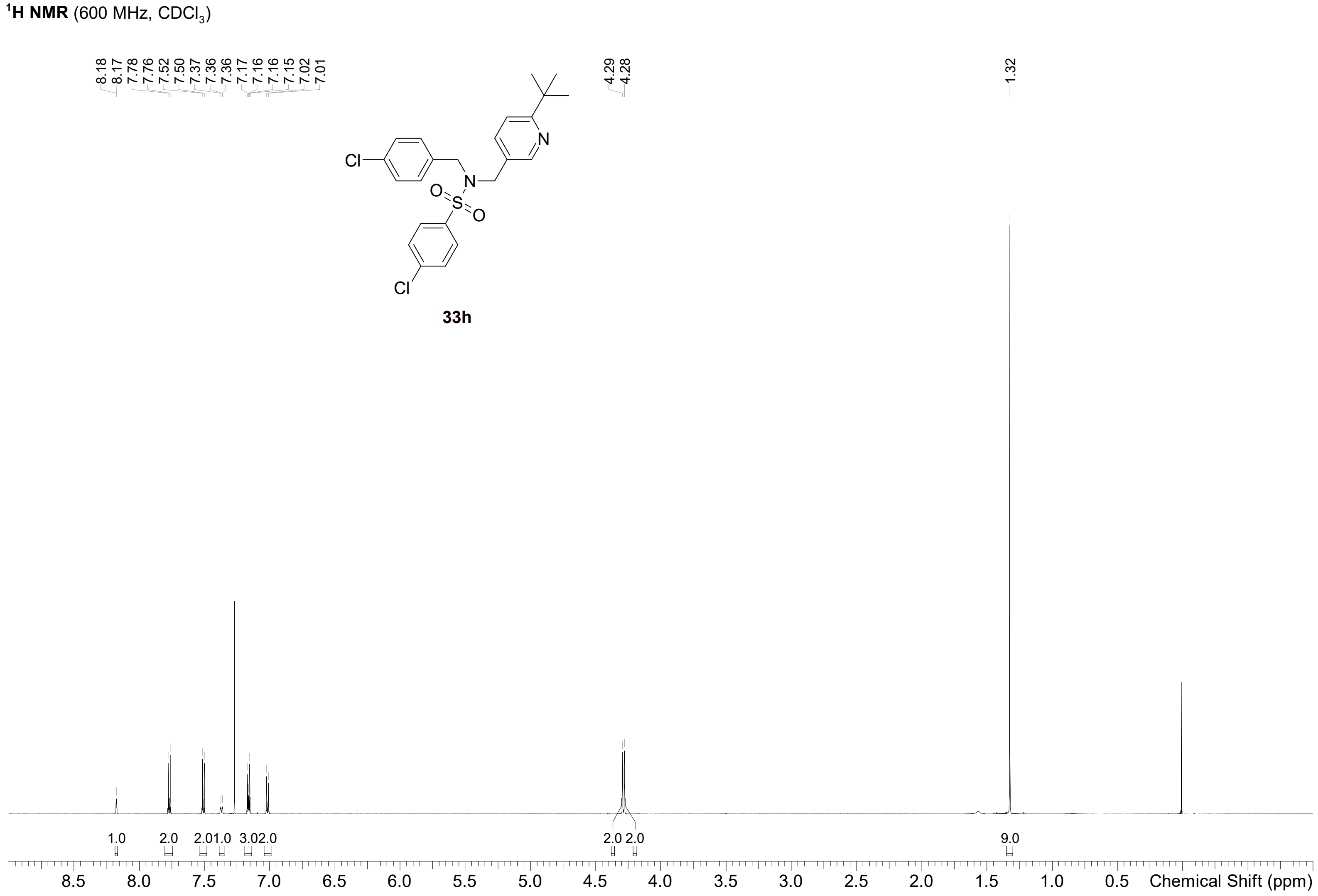

Figure 19: **33h**, ^{1}H-NMR spectrum.

^{1}H NMR (600 MHz, $CDCl_3$)

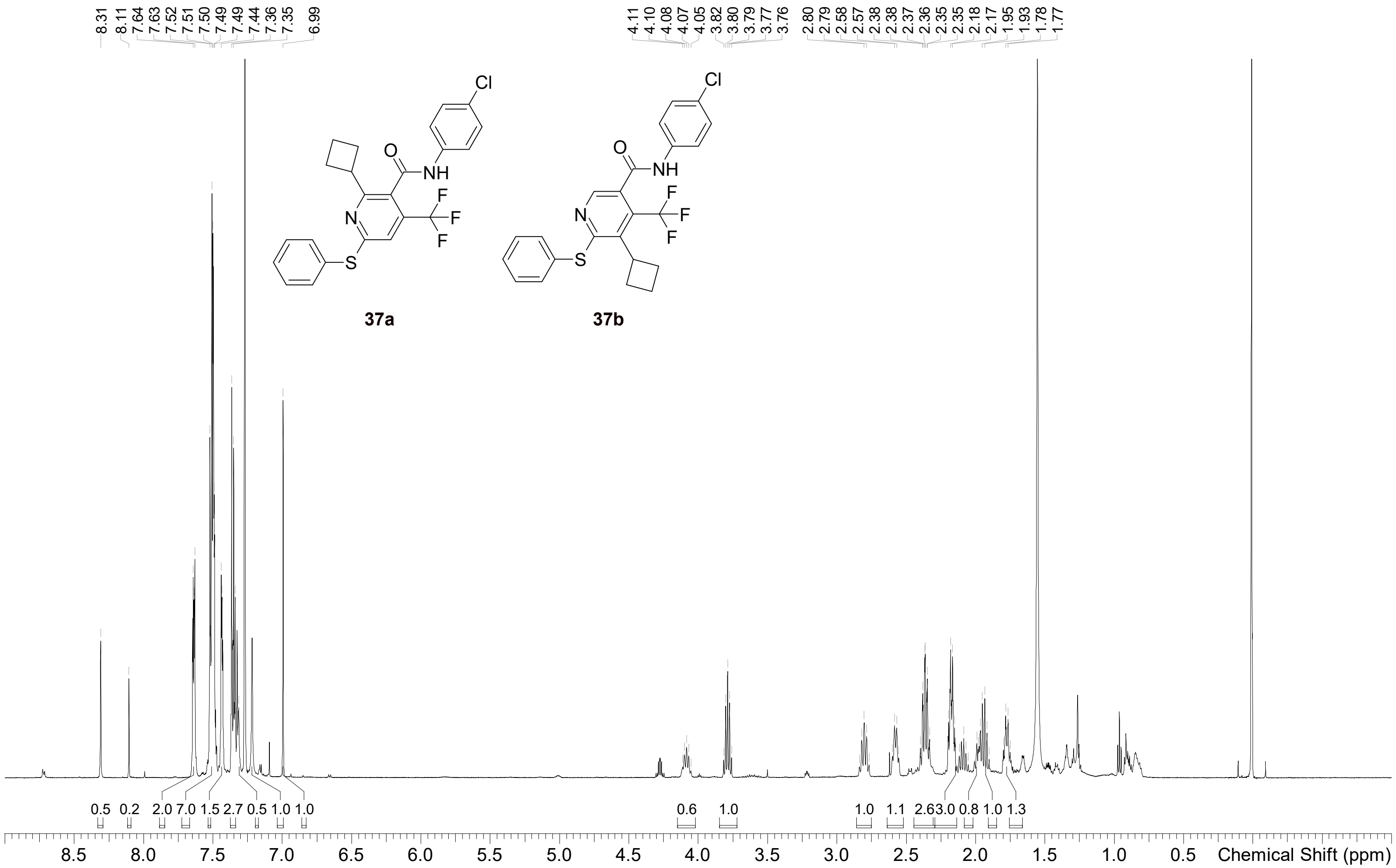

Figure 20: **37b1** & **37b2**, ^{1}H-NMR spectrum.

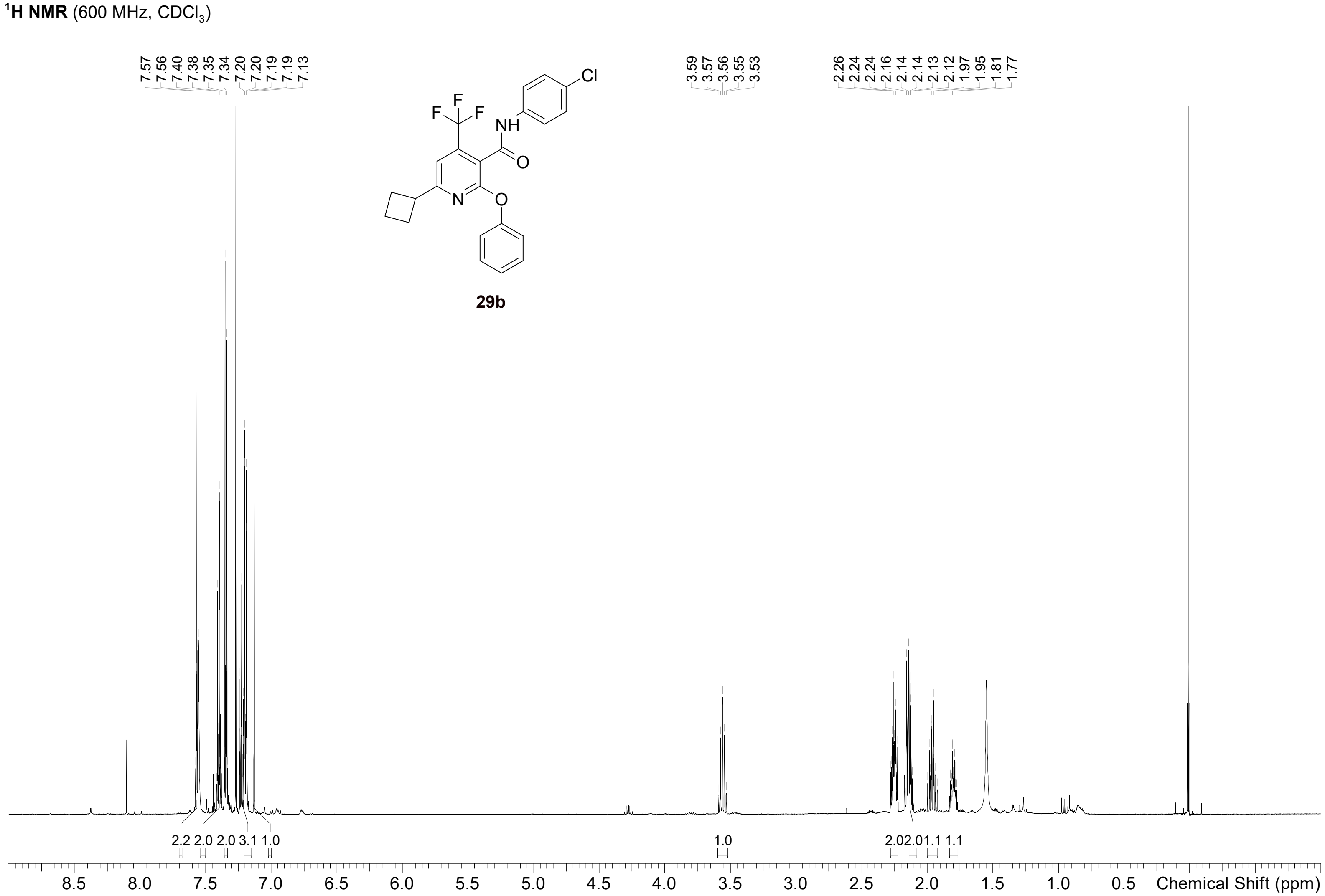

Figure 21: **29b**, ^{1}H-NMR spectrum.

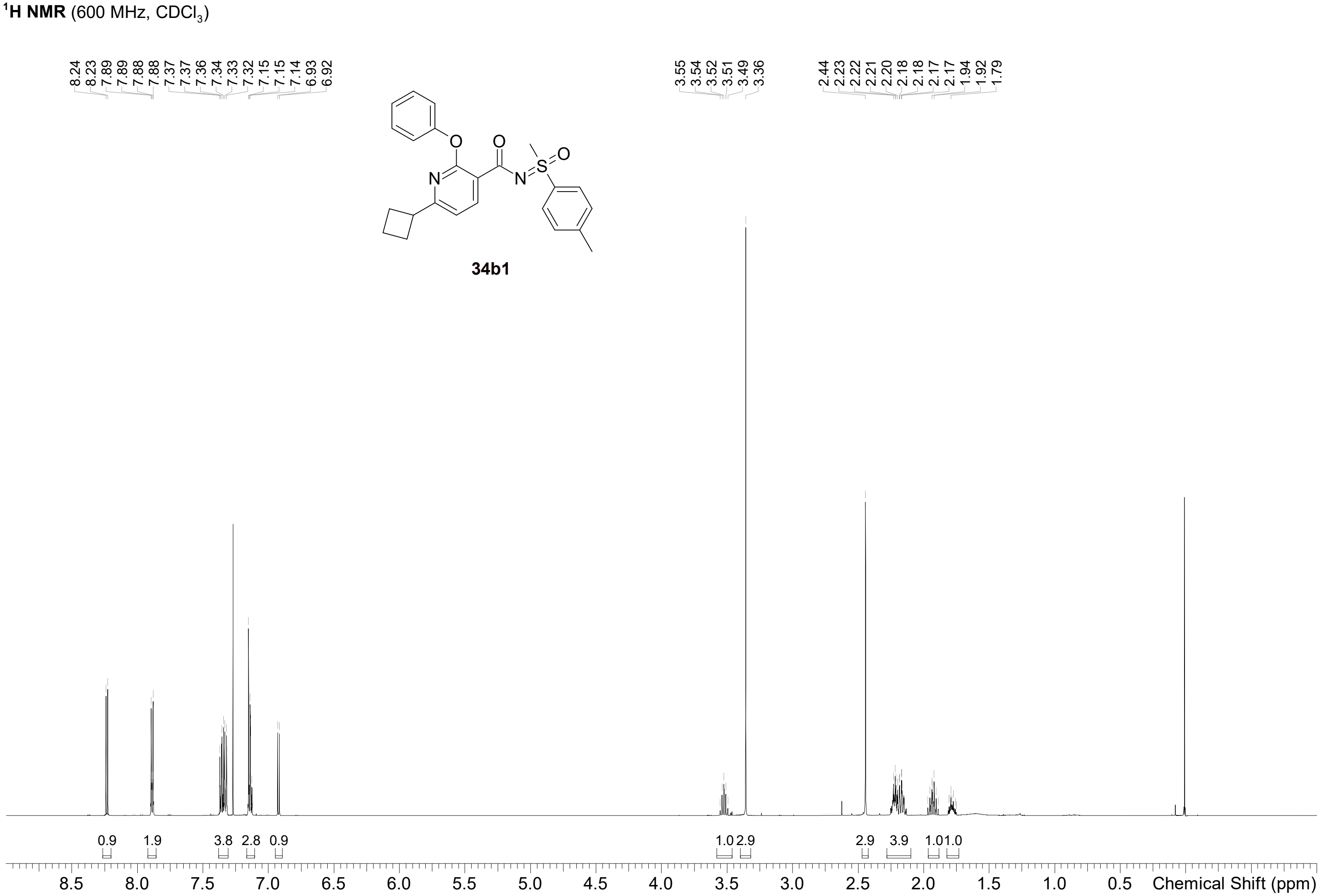

Figure 22: **34b1**, ^{1}H-NMR spectrum.

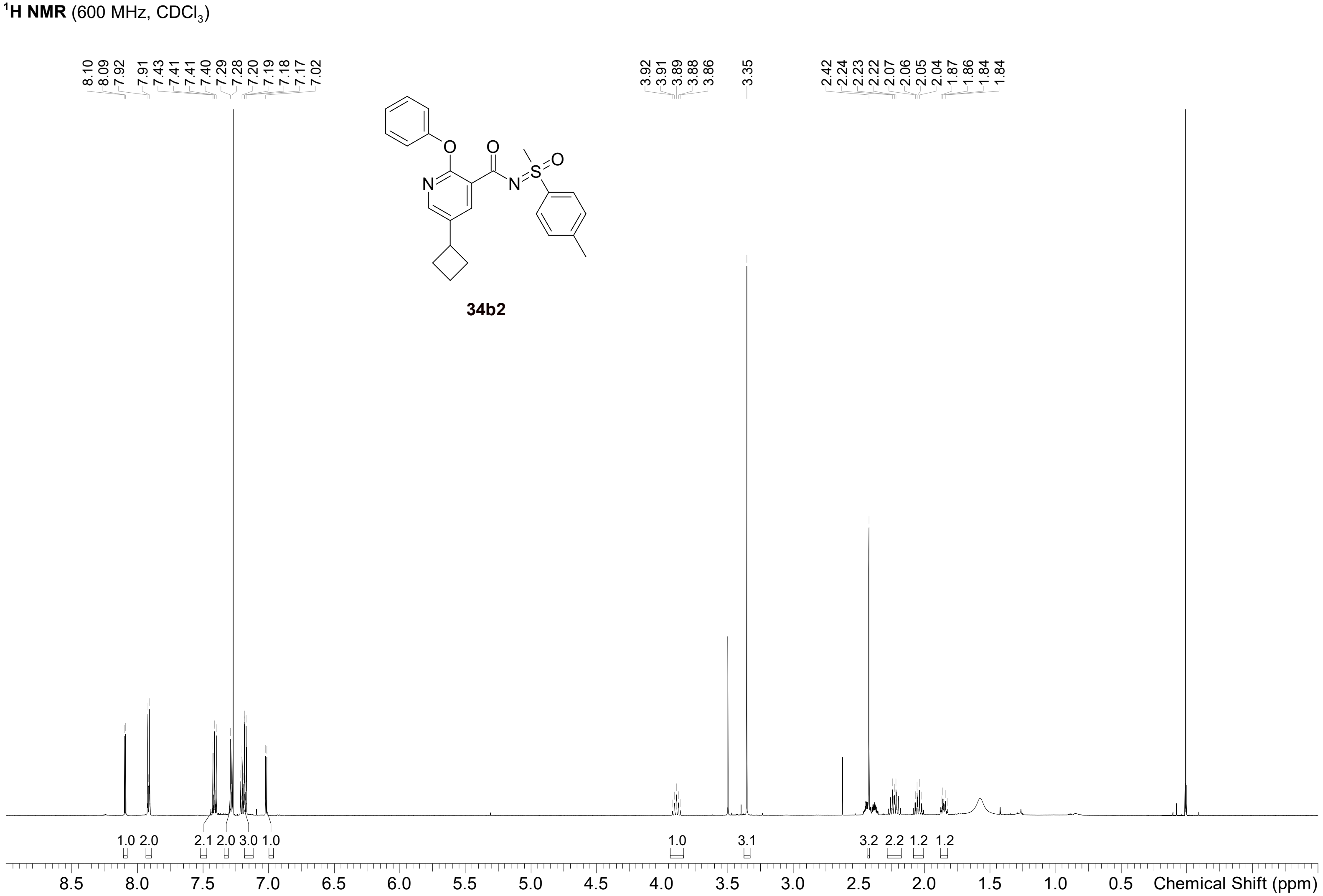

Figure 23: **34b2**, ^{1}H-NMR spectrum.

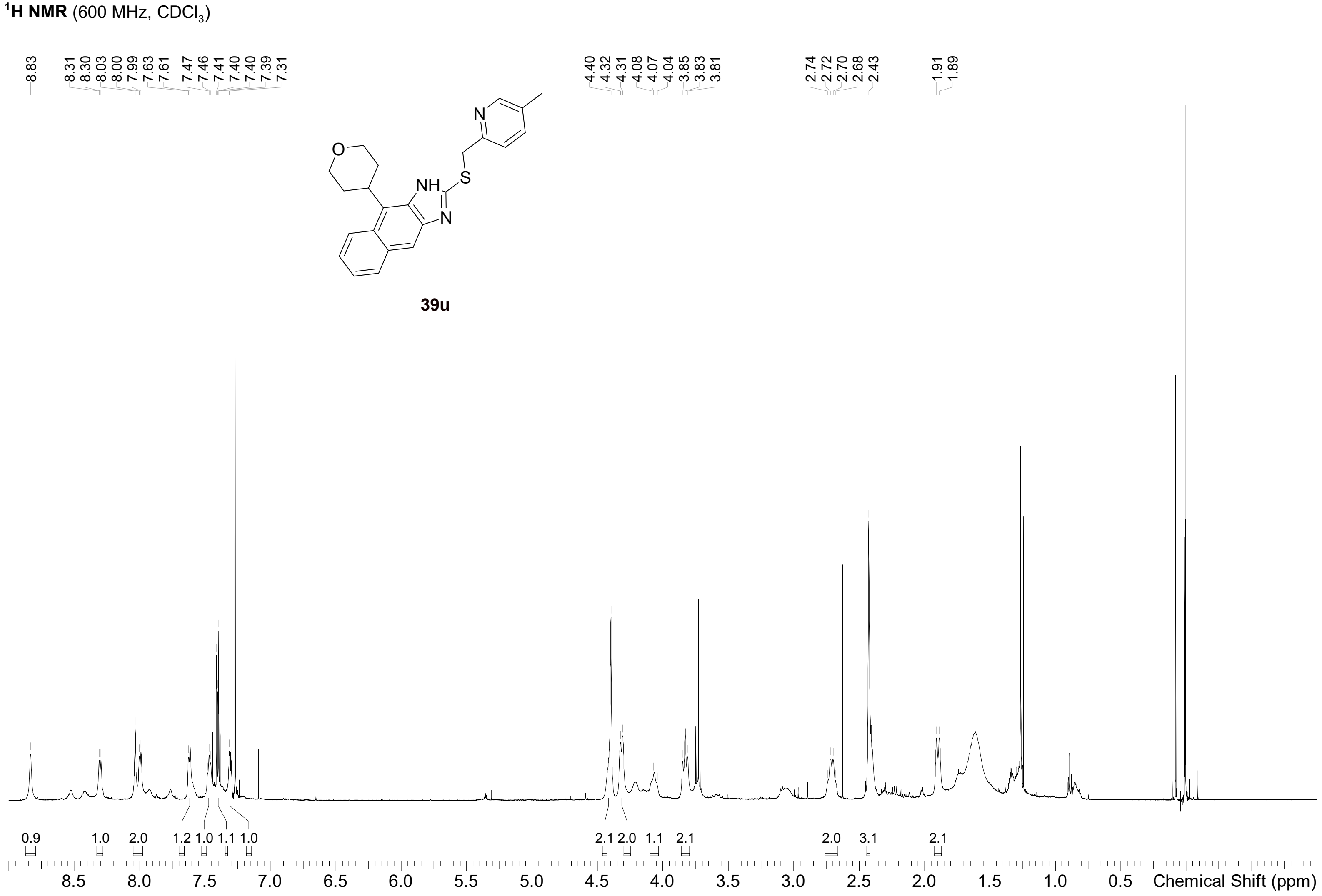

Figure 24: **39u**, ^{1}H-NMR spectrum.

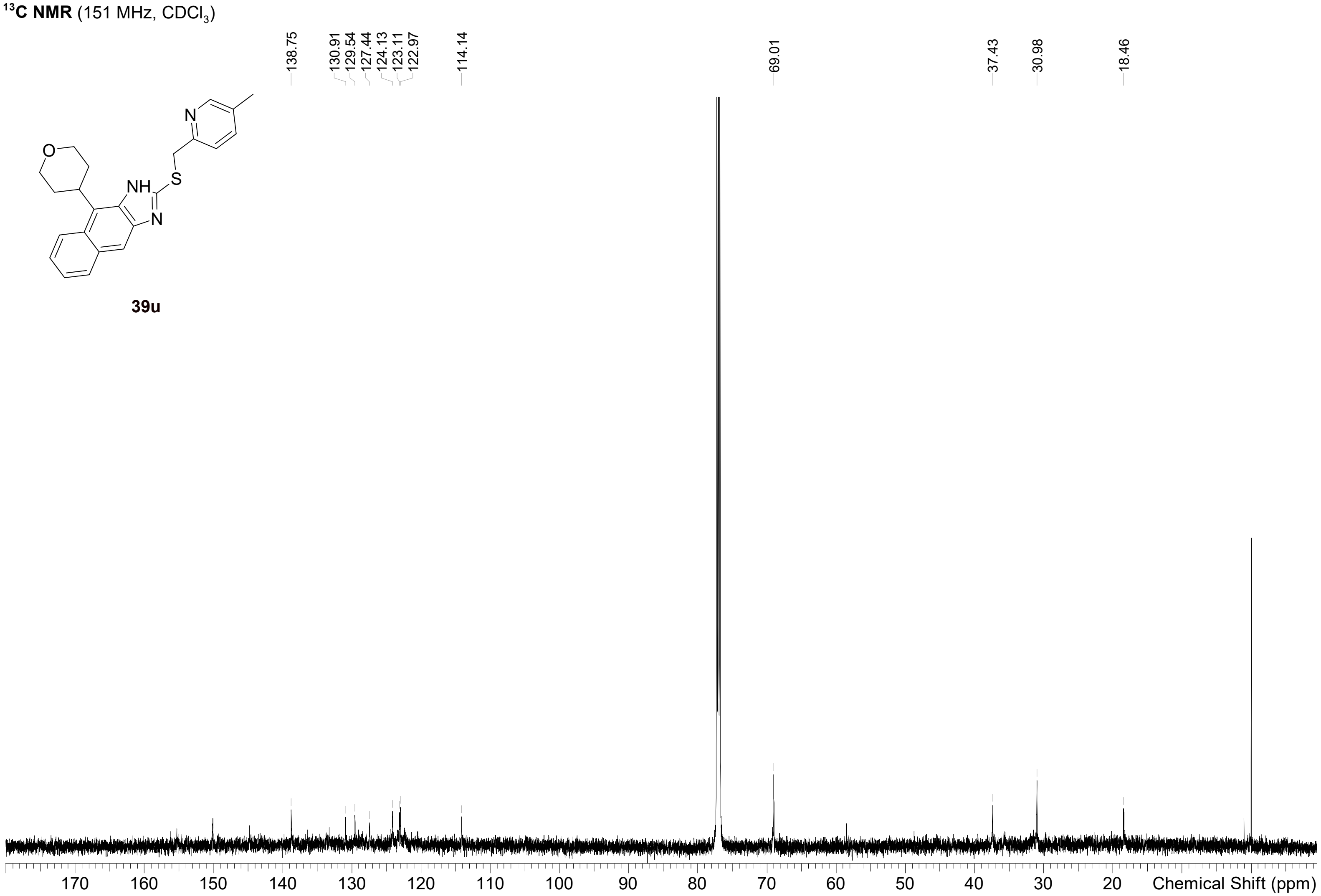

Figure 25: **39u**, ^{13}C-NMR spectrum.

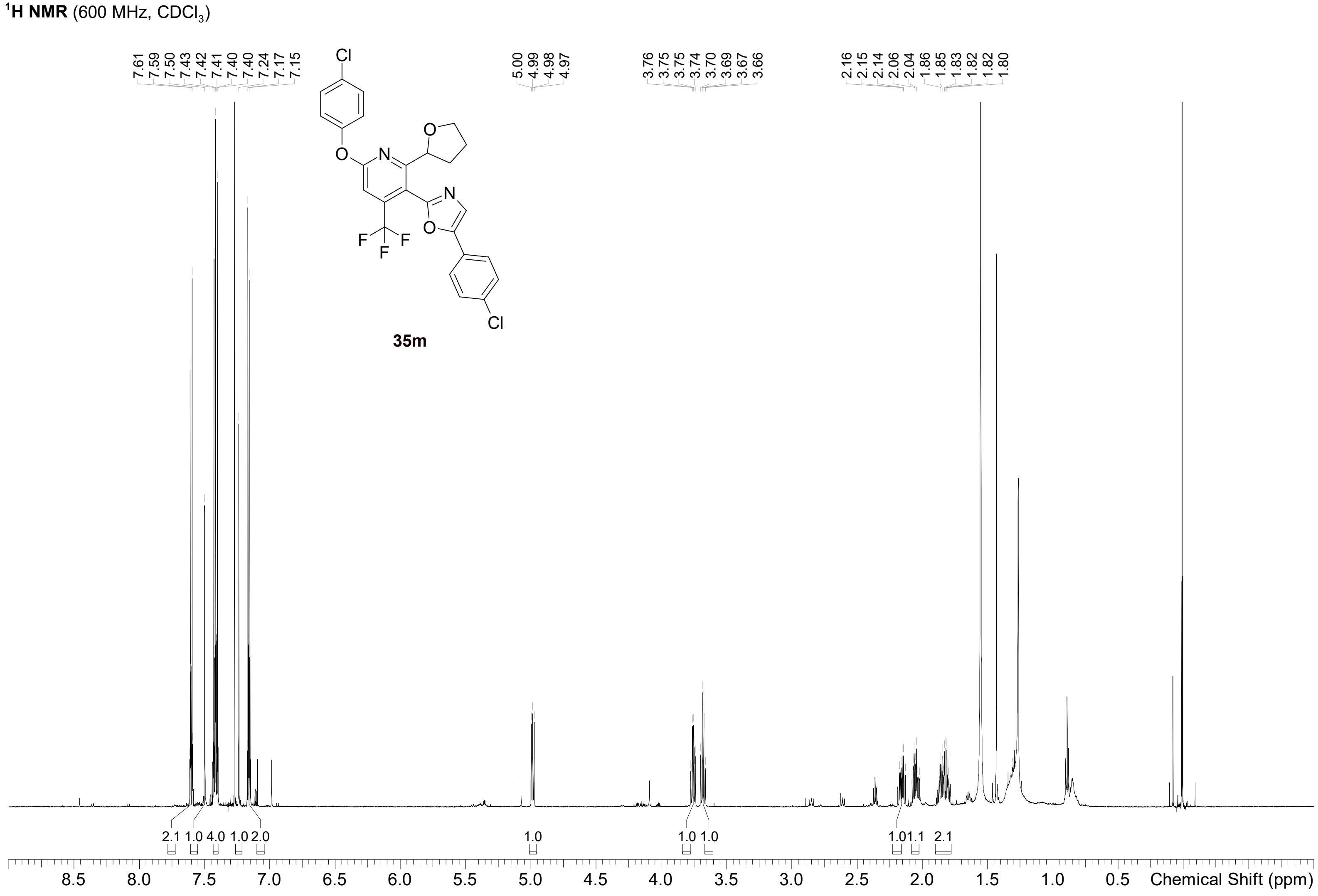

Figure 26: **35m**, ^{1}H-NMR spectrum.

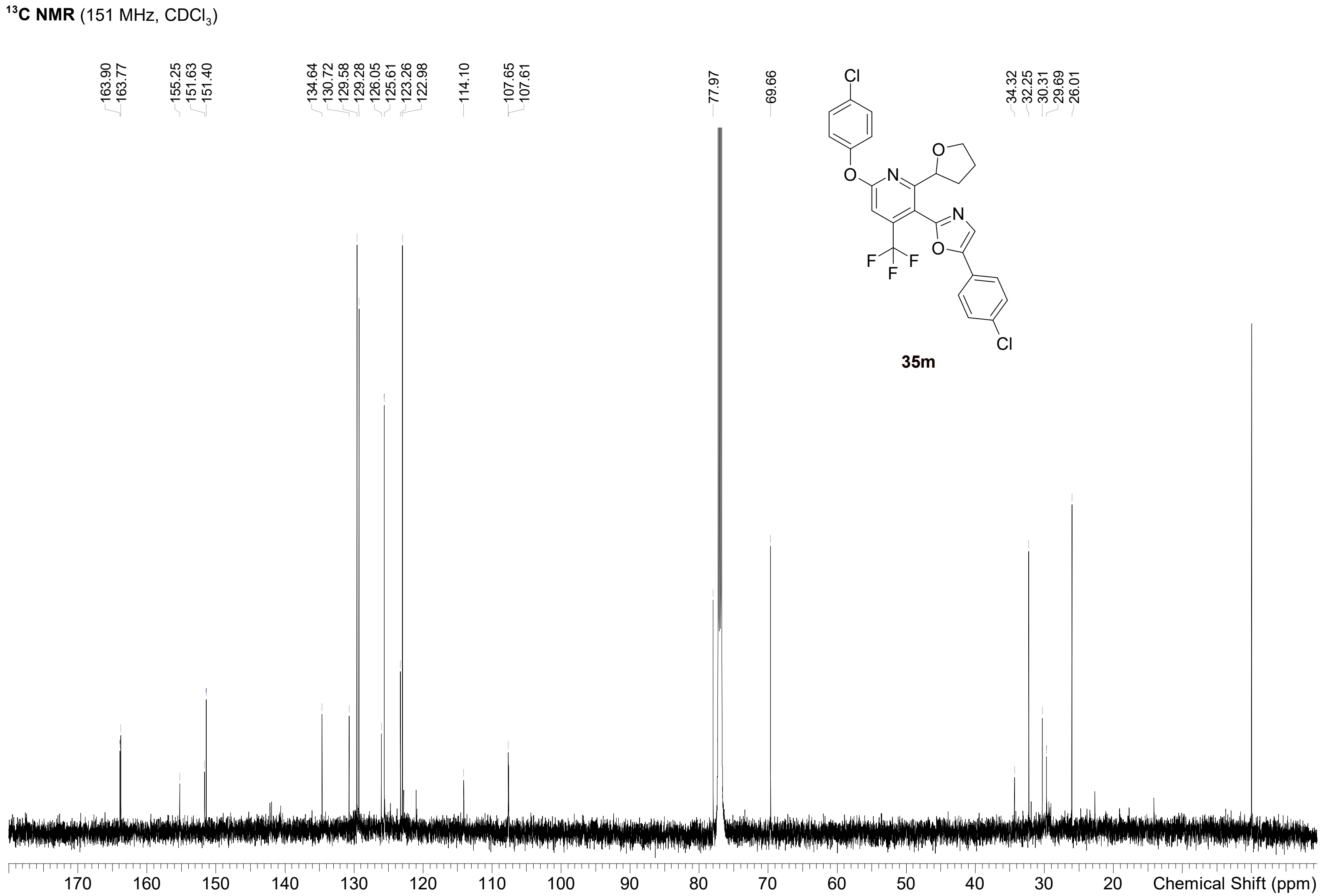

Figure 27: **35m**, ^{13}C-NMR spectrum.

^{1}**H NMR** (600 MHz, $CDCl_3$)

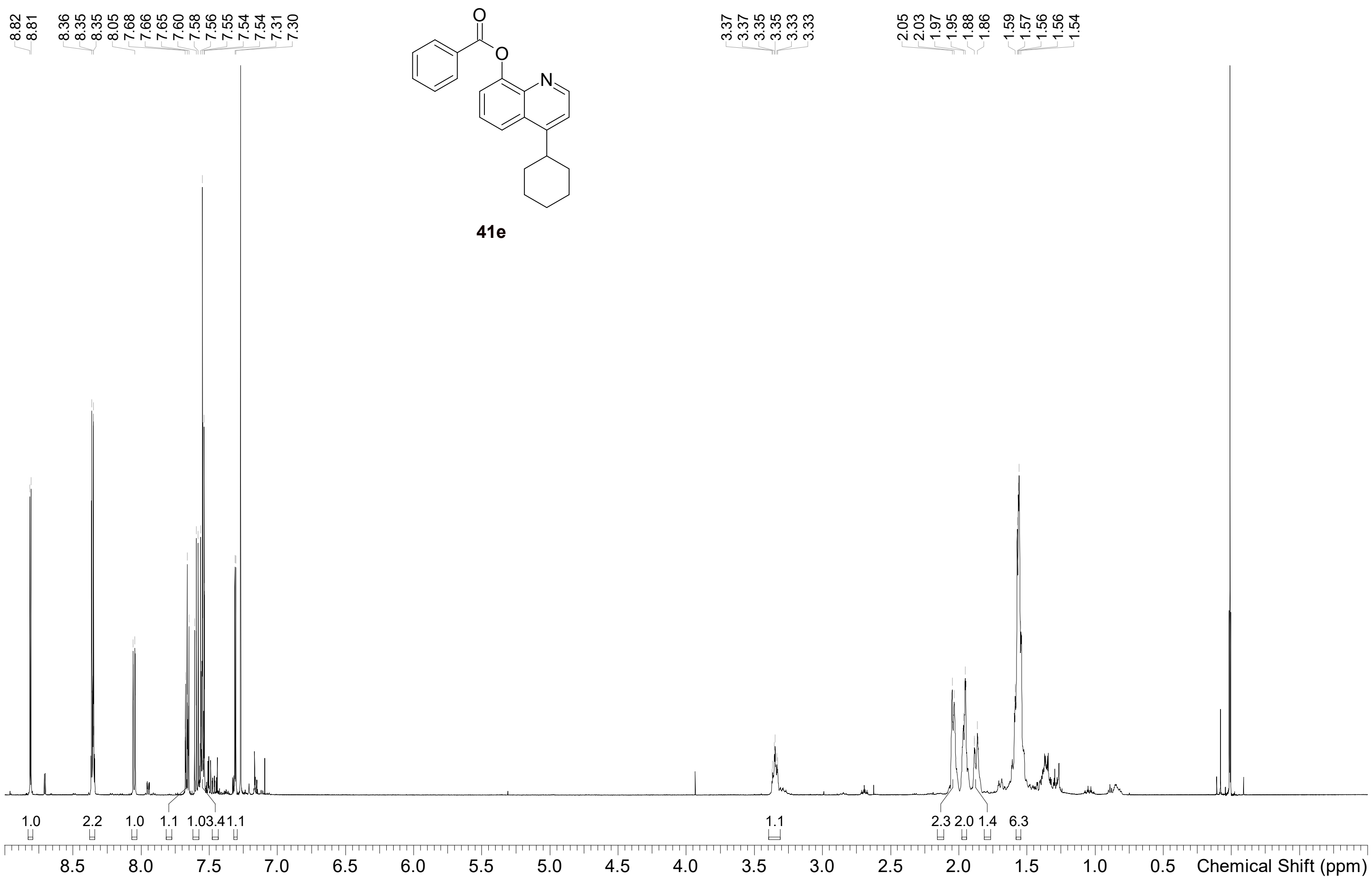

Figure 28: **41e**, ^{1}H-NMR spectrum.

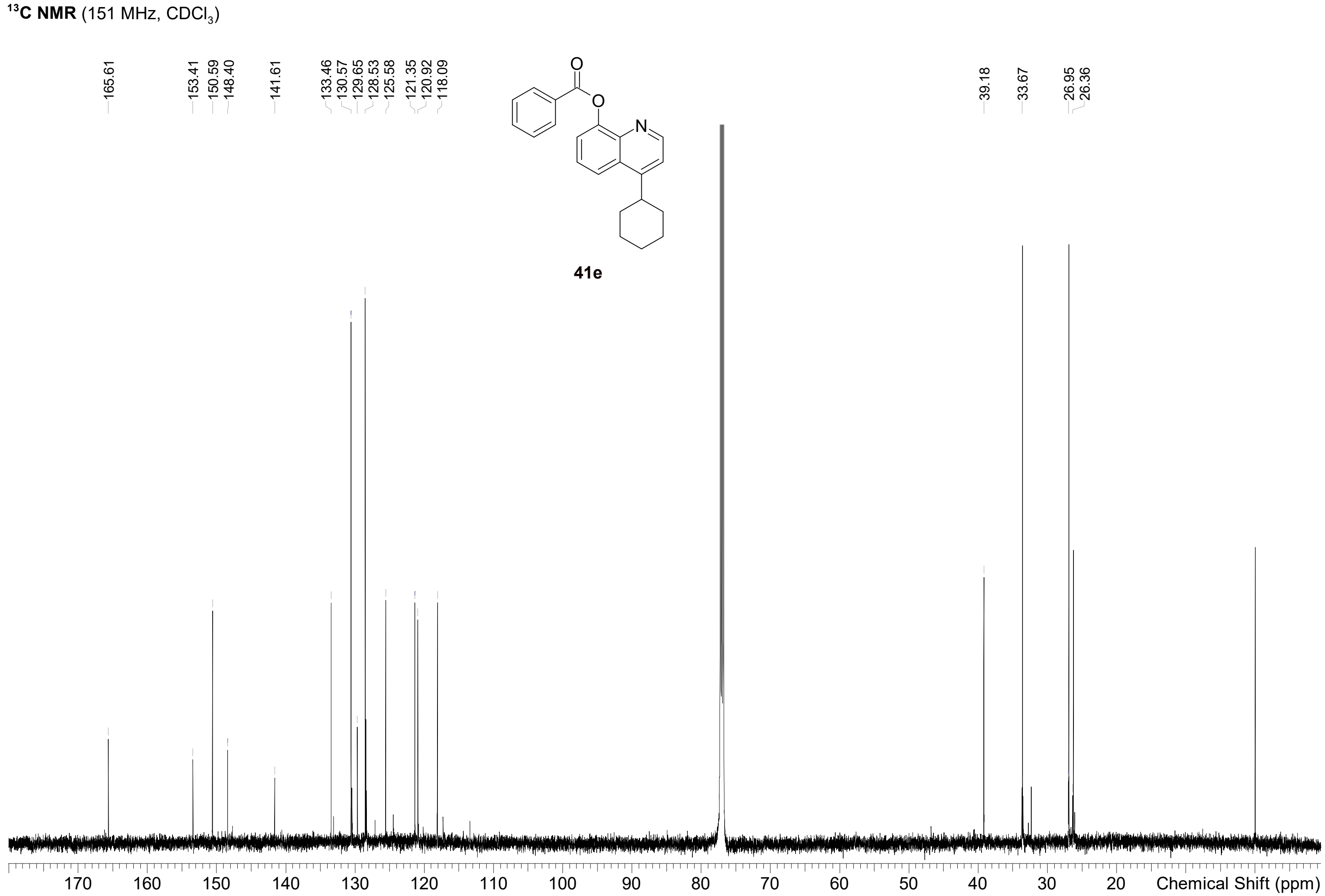

Figure 29: **41e**, ^{13}C-NMR spectrum.

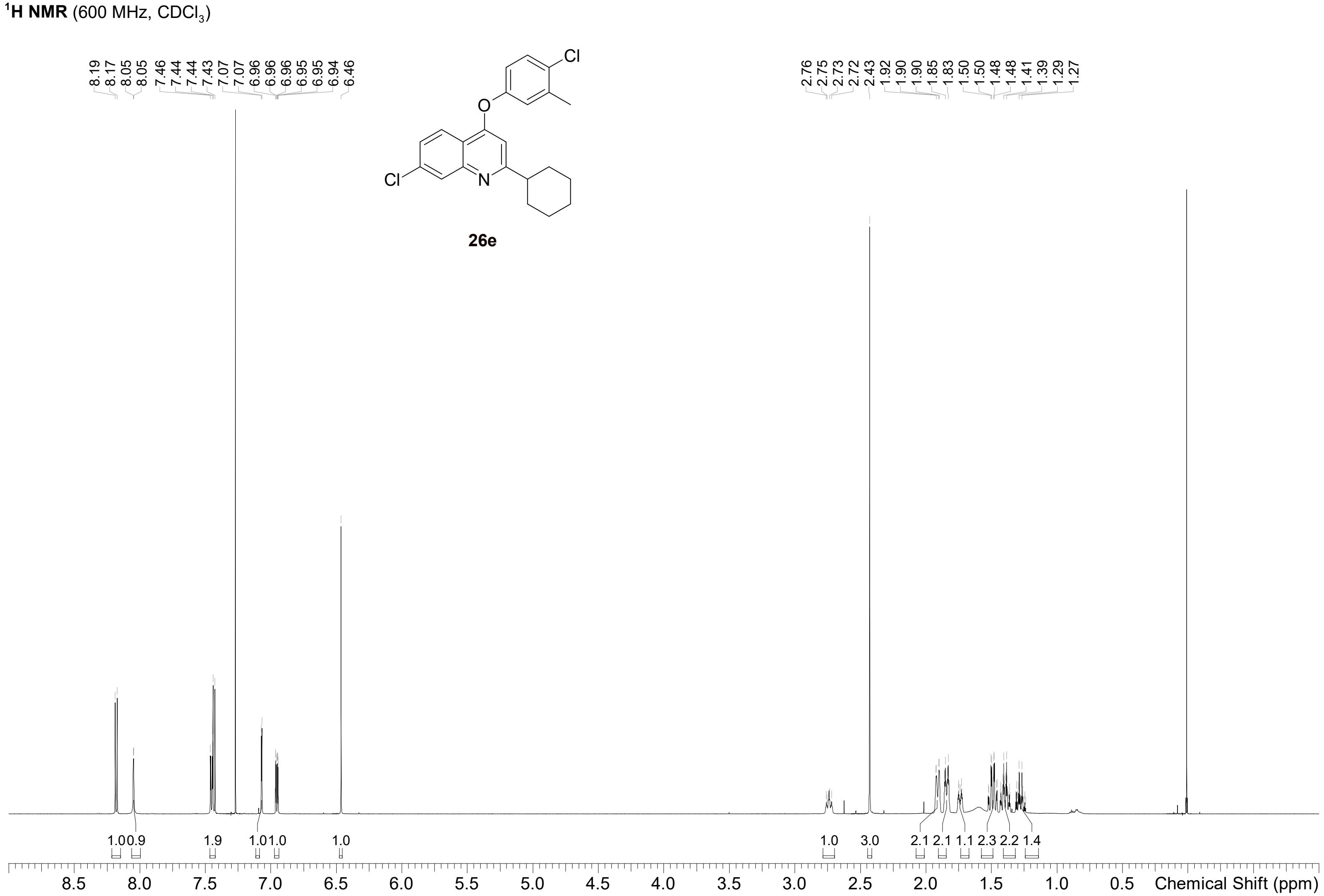

Figure 30: **26e**, ¹H-NMR spectrum.
